# Endothelin

**DOI:** 10.1124/pr.115.011833

**Published:** 2016-04

**Authors:** Anthony P. Davenport, Kelly A. Hyndman, Neeraj Dhaun, Christopher Southan, Donald E. Kohan, Jennifer S. Pollock, David M. Pollock, David J. Webb, Janet J. Maguire

**Affiliations:** Experimental Medicine and Immunotherapeutics, University of Cambridge, Cambridge, United Kingdom (A.P.D., J.J.M.); IUPHAR/BPS Guide to PHARMACOLOGY, Centre for Integrative Physiology, University of Edinburgh, Hugh Robson Building, Edinburgh, United Kingdom (C.S.); Division of Nephrology, University of Utah Health Sciences Center, Salt Lake City, Utah (D.E.K.); Cardio-Renal Physiology & Medicine, Division of Nephrology, Department of Medicine, University of Alabama at Birmingham, Birmingham, Alabama (K.A.H., J.S.P., D.M.P.); and Department of Renal Medicine, Royal Infirmary of Edinburgh (N.D.) and University/British Heart Foundation Centre for Cardiovascular Science, University of Edinburgh, Queen's Medical Research Institute (D.J.W.N.D.), Edinburgh, Scotland, United Kingdom

## Abstract

The endothelins comprise three structurally similar 21-amino acid peptides. Endothelin-1 and -2 activate two G-protein coupled receptors, ET_A_ and ET_B_, with equal affinity, whereas endothelin-3 has a lower affinity for the ET_A_ subtype. Genes encoding the peptides are present only among vertebrates. The ligand-receptor signaling pathway is a vertebrate innovation and may reflect the evolution of endothelin-1 as the most potent vasoconstrictor in the human cardiovascular system with remarkably long lasting action. Highly selective peptide ET_A_ and ET_B_ antagonists and ET_B_ agonists together with radiolabeled analogs have accurately delineated endothelin pharmacology in humans and animal models, although surprisingly no ET_A_ agonist has been discovered. ET antagonists (bosentan, ambrisentan) have revolutionized the treatment of pulmonary arterial hypertension, with the next generation of antagonists exhibiting improved efficacy (macitentan). Clinical trials continue to explore new applications, particularly in renal failure and for reducing proteinuria in diabetic nephropathy. Translational studies suggest a potential benefit of ET_B_ agonists in chemotherapy and neuroprotection. However, demonstrating clinical efficacy of combined inhibitors of the endothelin converting enzyme and neutral endopeptidase has proved elusive. Over 28 genetic modifications have been made to the ET system in mice through global or cell-specific knockouts, knock ins, or alterations in gene expression of endothelin ligands or their target receptors. These studies have identified key roles for the endothelin isoforms and new therapeutic targets in development, fluid-electrolyte homeostasis, and cardiovascular and neuronal function. For the future, novel pharmacological strategies are emerging via small molecule epigenetic modulators, biologicals such as ET_B_ monoclonal antibodies and the potential of signaling pathway biased agonists and antagonists.

## I. Historical Introduction

**Fig. 1.**
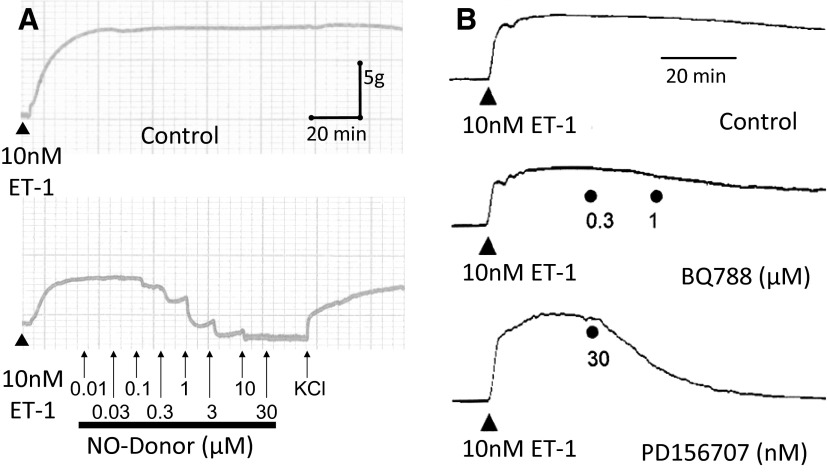
Long lasting vasoconstrictor response to 10 nM ET-1 in human mammary artery is maintained for over 2 hours but can be reversed by the physiologic antagonist nitric oxide derived from a nitric oxide donor (A) or by the ET_A_ antagonist PD156707 but not the ET_B_ antagonist BQ788 (B).

The vasoconstrictor actions of a factor obtained from the culture media of bovine aortic endothelial cells was first characterized in 1985 by Hickey et al. (1985) and was proposed to have the chemical composition of a peptide, because trypsin abolished the observed activity. The structure of this endothelium-derived constricting factor was identified in 1988 by Yanagisawa et al. (1988) from the supernatant of porcine aortic endothelial cells and named endothelin (now called endothelin-1 or ET-1). This remarkable paper ignited worldwide interest in both academia and the pharmaceutical industry by showing that ET-1 was the most potent vasoconstrictor identified to date, producing extremely powerful contraction of a range of mammalian blood vessels in vitro, including human arteries and veins. The response was unusually long lasting and difficult to wash out (Fig. 1). In the anesthetized denervated rat, in vivo ET-1 caused a rise in arterial pressure, and this pressor response was typically sustained for more than 1 hour. In the same year, the sarafotoxins, a family of peptides with high degree of sequence similarity to ET-1, were identified from the venom of a snake *Atractaspis engaddensis* or burrowing asp (Kloog et al., 1988; Takasaki et al., 1988). In accord with the actions of ET-1 in vivo, the symptoms of envenomation included very powerful contraction of the coronary arteries sufficient to cause the heart to stop. In humans, two further peptides, endothelin-2 (ET-2) and endothelin-3 (ET-3), were identified (Inoue et al., 1989) to complete the family of endogenous endothelin agonists. Pharmacological preparations such as rat aorta and rabbit pulmonary artery were initially identified that exhibited differences in the rank order of affinities for the three endogenous ET isoforms, suggesting the presence of two receptor subtypes. A year later, two novel G protein-coupled receptors (GPCRs) were identified: ET_A_ (Arai et al., 1990) where ET-1 and ET-2 were more potent than ET-3 (ET-1 = ET-2 > ET-3), and ET_B_ (Sakurai et al., 1990), where all three isoforms were equally effective (ET-1 = ET-2 = ET-3).

**Fig. 2.**
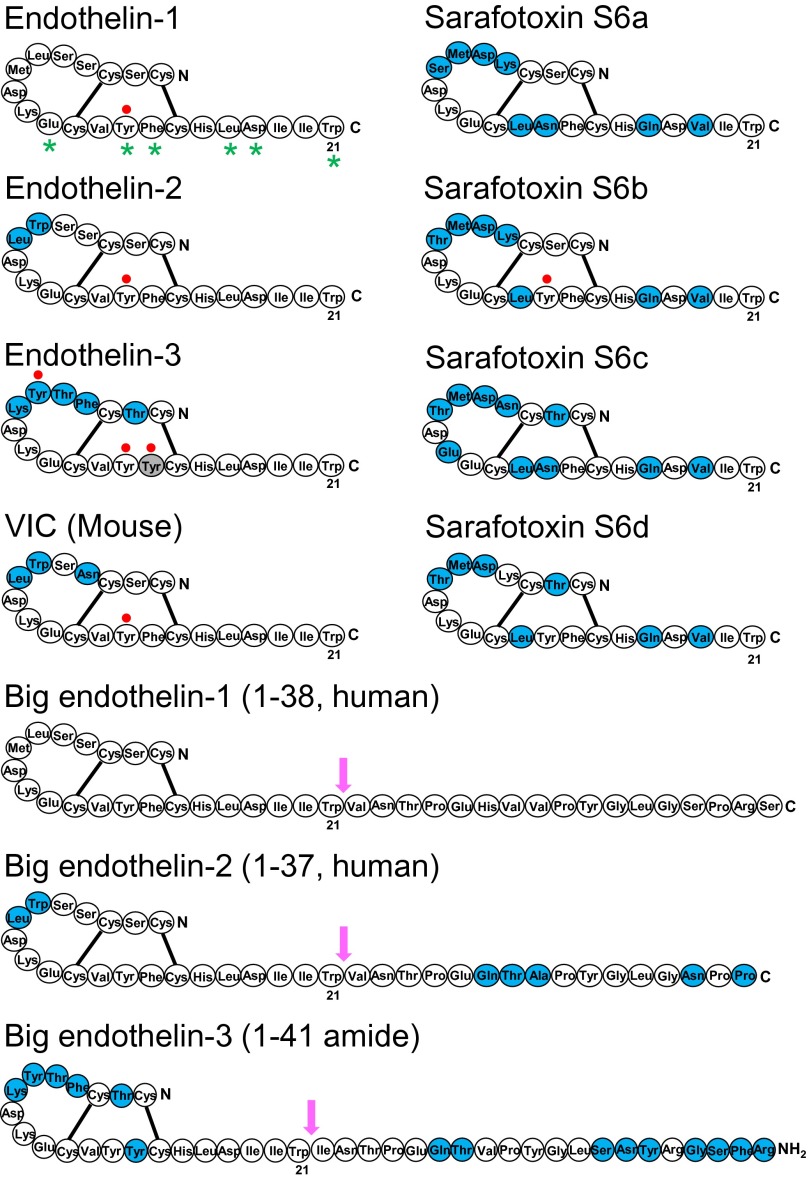
Schematic diagram showing the sequences of ET, Big ET precursors and sarafotoxin peptides. Amino acids that differ between ET-1 and mature peptides or between Big ET-1 and precursors are shown in blue. Labeling of [^125^I] radioligands at Tyr residues are shown by a filled red circle. Residues that are thought from X-ray crystallography to be aligned as a stripe as a result of a secondary helical structure secondary helical structure are indicated with a star (Janes et al., 1994; Orry and Wallace, 2000). These residues have also been shown experimentally to be crucial for binding (Huggins et al., 1993). Arrows indicate the site for cleavage of Big ETs to mature peptides: between Trp^21^-Val^22^ amino acids for Big ET-1 and ET-2 and between Trp^21^-Ile^22^ for ET-1 and for Big ET-3.

**Fig. 3.**
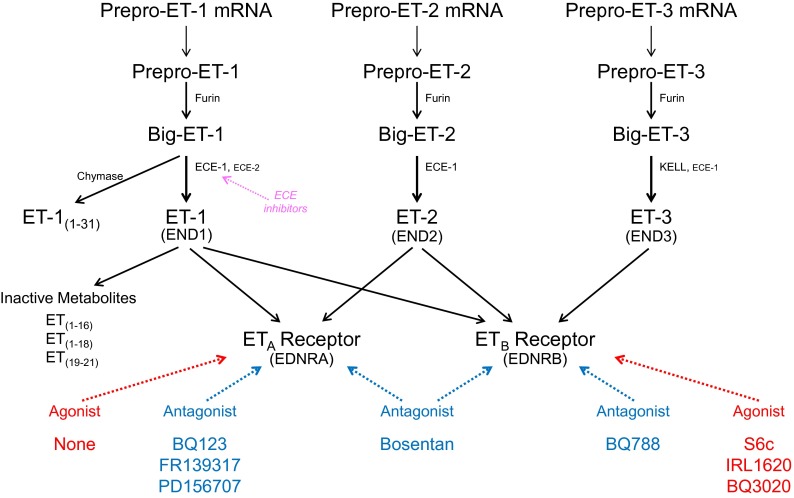
Schematic diagram illustrating synthesis of ET peptides and interaction with receptors. ET isoforms are synthesized by a three-step process: Messenger RNA encodes a prepro-peptide that after a proteolytic cleavage initial of the signal peptidase to yield the propeptide is further cleaved by furin to Big ET precursors. Synthesis of ET-1 has been studied in the most detail. Transformation to the mature, biologically active ET-1 is mainly by the action of ECE-1 at pH 7 but also by ECE-2 at pH 5.5 within endothelial cells. On release from endothelial cells, about one in five molecules of Big ET-1 escape conversion but further processing to ET-1 may occur by smooth muscle ECE or via alternative pathways catalyzed by chymase for ET-1. It is inferred that ET-2 from endothelial cells is synthesized by a similar pathway. ET-3 is not released from human endothelial cells but is also synthesized in other cells by ECE-1 with evidence for an additional pathway, KELL. ET-1 is metabolized by NEP to inactive metabolites. ETs mediate their actions via two GPCRs ET_A_ or ET_B_ and the recommended agonists and antagonists that are most widely used are indicated together with ECE inhibitors.

Yanagisawa et al. (1988) correctly predicted the biosynthesis of a 39-amino acid intermediate "Big endothelin" from proendothelin by proteolytic cleavage at paired basic residues and the subsequent production of the mature 21-amino acid peptide by a previously unknown processing pathway involving a putative "endothelin converting enzyme." The predicted endothelin converting enzyme-1 (ECE-1) was discovered (Takahashi et al., 1993, Xu et al., 1994). A second enzyme, ECE-2 (Emoto and Yanagisawa, 1995), was also identified. Thus within 7 years, all the essential components of the ET pathway had been revealed (Figs. 2 and 3).

Key milestones in the development of pharmacological agents were the first ET_A_-selective peptide antagonists, BQ123 (Ihara et al., 1992a) and FR139317 (Aramori et al., 1993). ET_B_ agonists were identified, including the linear or modified ET-1 sequences [Ala^1,3,11,15^]-ET-1 (Saeki et al., 1991), BQ3020; (Ihara et al., 1992b), and IRL 1620 (Takai et al., 1992) and the snake venom toxin sarafotoxin 6c (Williams et al., 1991). The first selective peptide ET_B_ antagonist was BQ788 (Ishikawa et al., 1994). It was very clear that the clinical goal was the discovery of orally active, small molecule ET receptor antagonists that would block the potent and long lasting vasoconstrictor actions of ET in the human cardiovascular system. Within 5 years of the discovery of ET-1, the first ET_A_/ET_B_ antagonist with oral bioavailability was identified by Clozel et al., (1993). Its successor bosentan (Tracleer; Actelion, Allschwil, Switzerland), also a mixed ET_A_/ET_B_ antagonist, was the first in class to be introduced into the clinic, initially for the treatment of pulmonary arterial hypertension (PAH) (Rubin et al., 2002).

An ET_A_-selective antagonist ambrisentan (Letairis, Volibris; Gilead, Foster City, California) was approved for clinical use in PAH in 2007 (Vatter and Seifert, 2006), followed by the more ET_A_-selective antagonist sitaxentan (Thelin; Pfizer, Groton, Connecticut) (Benza et al., 2007; Galie et al., 2009). However, in 2010 sitaxentan was voluntarily withdrawn by Pfizer (Groton, Connecticut) owing to cases of idiosyncratic hepatitis, resulting in mortality from acute liver failure (Don et al., 2012). Bosentan was the structural basis for the development of macitentan, approved for clinical use in 2013 and representing the next generation of antagonists, being more potent, with longer receptor occupancy as well as undergoing conversion to an active metabolite, properties contributing to greater pharmacodynamic and pharmacokinetic efficacy (Patel and McKeage, 2014). Despite the success of both mixed receptor and ET_A_-selective antagonists, the second pharmacological strategy of reducing ET-1 levels by inhibiting ECE has yet to be proven successful in the clinic.

The aim of this review is to focus on key concepts in the pharmacology of ETs, their receptors, and the interaction of the signaling pathway with agonists, antagonists, and synthetic enzyme inhibitors, particularly in humans, in vitro and in vivo. Remarkably all ET peptides, receptors, and synthetic enzymes have been deleted in mice with many components selectively deleted or overexpressed in specific cell types. The resulting phenotypes have provided insights into their role in the ET signaling pathway, which are evaluated in this review. The third aim is to summarize advances in the clinical pharmacology of ET agents. To date nearly 28,000 endothelin-related papers and reviews have been published. The following should be consulted for more detailed information on ET pharmacology, physiology, and pathology: antagonists (Kohan et al., 2012; Clozel et al., 2013; Maguire and Davenport, 2015), the heart (Drawnel et al., 2013; Chester and Yacoub, 2014; Nasser and El-Mas 2014), kidney (Kohan et al., 2011a,b; Hyndman and Pollock, 2013; Boesen, 2015; Culshaw et al., 2015), vasculature (Sandoval et al., 2014), hypertension (Schiffrin, 2001; Rautureau and Schiffrin, 2012; Speed and Pollock, 2013; Laffin and Bakris, 2015), atherosclerosis and diabetes (Pernow et al., 2012), cancer (Rosanò et al., 2013a; Irani et al., 2014), and PAH (Moorhouse et al., 2013; Miyagawa and Emoto 2014). The International Union of Pharmacology/British Pharmacological Society (IUPHAR/BPS) curated database, Guide to PHARMACOLOGY, provides detailed information on pharmacological parameters for ET ligands (Davenport et al., 2015).

## II. Endothelins and Sarafotoxins

### A. Endothelin-1 and Big Endothelin-1

The structure of ET-1, considered the parent compound of the family, is shown in Fig. 2 comprising 21 amino acids with a free amino terminus and C-terminal carboxylic acid. Within the endothelins and sarafotoxins residues 1, 3, 8, 10, 11, 15, 16, 18, 20, and 21 are always conserved (Huggins et al., 1993). The ETs are unusual compared with other bioactive peptides because the amino acid residues are linked by two disulfide bonds. A number of three-dimensional nuclear magnetic resonance (NMR) structures have been proposed for ET-1 but these have not produced a consensus for its conformation and are difficult to interpret (Wallace and Janes, 1995). ET-1 is one of a very few vasoactive peptides that have been successfully crystalized and the X-ray structure has been solved (Janes et al., 1994). This differs from the various NMR models, particularly in the C-terminal helical tail that is crucial for receptor interaction, and the proposed structure is supported by binding data. A major reason for the variability in NMR is that these use ET-1 in organic solvents, whereas X-ray studies were carried out on crystals from an aqueous solution that better reflects the in vivo environment.

The structure of ET-1 is unusual among the mammalian bioactive peptides in possessing two intramolecular disulfide bonds between Cys residues crosslinked at positions 1 and 15 and 3 and 11 (Fig. 2). Experimentally, residues at positions 10, 13, 14, 17, 18, and 21 are crucial for binding (Huggins et al., 1993; Janes et al., 1994; Orry and Wallace, 2000), with loss of Trp^21^ for example completely abolishing activity. These residues are located consecutively, separated by two amino acids, which is consistent with a secondary helical structure. X-ray crystallography also suggests there is a helical structure, and these residues are aligned as a stripe (Fig. 2).

Radioligand binding studies using [^125^I]-Big ET-1 demonstrated unequivocally that at physiologic concentrations, despite the presence of the mature sequence within the molecule, Big ET-1 does not bind to either receptor subtype (Russell et al., 1998). Molecular modeling shows that the accessibility for binding of amino acids 1–5 are greatly reduced in Big ET-1 compared with ET-1 and that residues 16–21 are also affected as a result of the folding back of the C terminus of Big ET-1. In addition, Big ET-1 is resistant to proteolytic cleavage by enzymes that are able to metabolize the mature peptide (Peto et al., 1996). As a result, inhibition of the processing of Big ET-1 to ET-1 is an attractive target for lowering endogenous levels of the mature peptides through selective ECE inhibition.

#### 1. Endothelin-1 Synthesis within Endothelial Cells.

ET-1 is the most abundant isoform in the human cardiovascular system and the primary source is thought to be vascular endothelial cells, although the peptide is produced by other cell types, including epithelial cells, for example in the lungs, kidney, and colon; macrophages and monocytes, enteric glia cells in the periphery, as well as choroid plexus and certain neurons and reactive glial cells in the central nervous system. However, most of the information about ET-1 synthesis has been determined from studying endothelial cells that form a single layer of cells lining every blood vessel in the cardiovascular system that have a mass comparable with other endocrine glands. ET-1 has been detected in endothelial cells in all types of vessel, from large conduit arteries, resistance arteries (contributing to the maintenance of blood pressure), large veins, and venules. ET-1 is likely to be present and have a role in controlling perfusion in every organ in the body.

ET-1 is synthesized and released continuously from endothelial cells, and levels of pre-proendothelin (preproET-1) are modulated predominantly at the level of transcription, with evidence implicating numerous transcription including, activator proton 1 (AP-1), nuclear factor kappa B, FOXO1, VezF1, HIF-1, and GATA2. Both physical and chemical stimuli contribute to alterations in levels of preproET-1 mRNA in physiologic and pathophysiological conditions [e.g., shear stress, hypoxia, thrombin, and vasoactive factors such as angiotensin II (ANGII)] (for a detailed review of ET-1 gene regulation, see Stow et al., 2011). In the vasculature, shear stress is critical in determining the balance between ET-1 and NO production, and, at least in mice in vivo, shear stress alteration in endothelial gene expression appears to involve AMP-activated protein kinase stimulation of the anti-inflammatory transcription factor Krüppel-like factor 2 (Young et al., 2009). Hypoxia, for example in tumors, also has an important role in increasing expression of endothelial genes including ET-1 that possess hypoxic responsive elements in their promotors, contributing to disease progression (Spinella et al., 2014). One of the most important regulators of ET-1 production in endothelial cells is transforming growth factor (TGF) *β*. It has been demonstrated in bovine aortic endothelial cells in culture that TGF*β* signaling via the ALK5/Smad 3 pathway results in an increase in preproET-1 via an AP-1 site and/or smad binding element (Castañares et al., 2007), resulting in a mature cell phenotype.

#### 2. ECE-1 Synthetic Pathway.

**Fig. 4.**
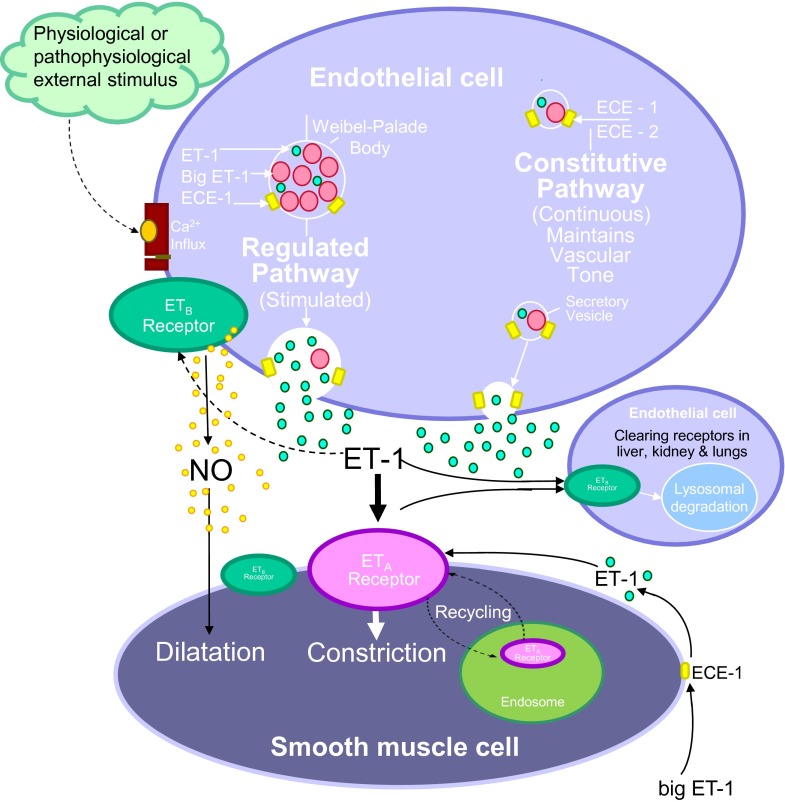
ET-1 is synthesized in human endothelial cells by a dual secretory pathway. ET-1 is continuously released from the small vesicles of the constitutive pathway to interact with ET receptors to contribute to vasomotor tone. ET-1 is also released from the regulated pathway in response to external stimuli from Weibel-Palade bodies that are unique to endothelial cells. In the human vasculature, ET-1 released abluminally from endothelial cells, interacts mainly with ET_A_ receptors on the underlying smooth muscle, with a small population of ET_B_ receptors also mediating constriction in some but not all vessels. In animals, vasoconstriction can be via ET_B_ or a mixture of both depending on the vascular bed. The ET-1/ET_A_ complex undergoes internalization to the endosome before recycling of the receptor to the cell surface and provides a mechanism whereby ET_A_ antagonist can reverse an ET-1 response. Some ET-1 may also interact with endothelial ET_B_ receptors in an autocrine manner and limit the constrictor response by the release of vasodilators such as nitric oxide. ET-1/ET_B_ complex is internalized and degraded to the lysosome; as a result ET_B_ antagonists are unable to displace receptor bound ligand. Low levels of ET-1 and Big ET-1 that has escaped conversion can also be detected in the plasma. Big ET-1 can also undergo further conversion by smooth muscle ECE to the mature peptide.

ET-1 is synthesized in a three-step process (Figs. 3 and 4). The ET-1 gene encodes a 212-amino acid precursor, preproET-1. Removal of the 17-amino acid signal by a signal peptidase generates proET-1, which in turn is cleaved at both the C and N terminals by furin enzymes, which remove 35 and 122 amino acids, respectively, to yield Big ET-1 (Xu et al., 1994; Turner and Murphy, 1996). PC7 has also been proposed as a second convertase (Blais et al., 2002); however, deleting furin in endothelial cells substantially reduced generation of ET-1 (Kim et al., 2012). Furins activate a wide range of proteins by cleaving basic amino pairs of Arg and Lys, and this stage is not tractable to selective inhibition of the ET pathway.

In contrast, the final conversion is affected by an unusual but selective hydrolysis of Trp^21^-Val^22^ by activity of ECE-1. ECE-1 belongs to the type II membrane-bound zinc metalloprotease family and exhibits maximal activity at pH 7.0 for the cleavage of Big ET-1. ECE-1 comprises a short cytoplasmic N-terminal tail, a membrane-spanning region, and a large extracellular domain containing a zinc-binding motif essential for enzymatic activity. Four isoforms, ECE-1a–d (Schweizer et al., 1997; Valdenaire et al., 1999), derived from a single gene by differential splicing of mRNA transcripts, have been identified in humans. The enzymes differ only in the amino acid sequence of the extreme N terminal and show comparable efficiency of conversion of Big ET-1 (Jeng et al., 2002). They do differ in their subcellular localization, although the relative levels of the isoform mRNA species vary between human tissues: ECE-1c mRNA is generally the predominant isoform. ECE-1a and ECE-1c were localized at the cell surface; ECE-1b was intracellular and showed significant colocalization with a marker protein for the trans-Golgi network (Schweizer et al., 1997). The fourth isoform (ECE-1d) was also identified, with converting activity comparable with that of the other three isoenzymes. In contrast to ECE-1b, ECE-1d is expressed at the cell surface, although less strongly than ECE-1a (Valdenaire et al., 1999).

ECE-1 is widely expressed in human tissues (Rossi et al., 1995; Davenport et al., 1998; Davenport and Kuc, 2000) and intriguingly is upregulated in a number of cancers (Smollich et al., 2007; Whyteside et al., 2014). Term embryos of ECE-1 knockout (KO) mice (ECE-1−/−) (section V.B.1) exhibited similar craniofacial and cardiac abnormalities to those seen in ET-1- and ET_A_-deficient embryos. Unexpectedly, in view of the lowered efficiency of ECE-1 in generating ET-3 from Big ET-3, epidermal melanocytes and enteric neurons of the distal gut were also absent in these animals, similar to the developmental phenotype seen in ET-3−/− and ET_B_ KO mice (Yanagisawa et al., 1998b).

ET-1 is unusual for vasoactive peptides in being synthesized by a dual pathway (Fig. 4). ECE-1 has been visualized in small secretory vesicles of the constitutive pathway, from where ET-1 is thought to be continuously released to maintain normal vascular tone (Haynes and Webb, 1994). ET-1 has also been localized together with ECE-1 and ECE-2 in specialized Weibel-Palade storage granules that are unique to endothelial cells and that are an integral component of the regulated pathway. These structures degranulate after external physiologic or pathophysiological stimulus (chemical or mechanical), releasing ET-1 to produce further vasoconstriction (Russell et al., 1998; Russell and Davenport, 1999a). Big ET-1 that has escaped conversion to ET-1 within endothelial cells can be detected in the plasma. ECE-1 has also been localized to smooth muscle cells in human blood vessels and converts Big ET-1 in vitro and is upregulated in atherosclerosis (Maguire et al., 1997; Maguire and Davenport, 1998). Given the larger volume of the smooth muscle compared with the single layer of endothelium, smooth muscle ECE may be a more important source of ET-1 in pathophysiological conditions. Plasma levels of ET-1 are typically ∼5 pmol/l, suggestive of a locally released rather than a circulating peptide (Davenport et al., 1990). In comparison Big ET-1 is detected at ∼1 pmol/l in plasma (Suzuki et al., 1991).

#### 3. ECE-2 Synthetic Pathway.

ECE-2 also belongs to the type II membrane-bound zinc metalloprotease family and shares 59% sequence similarity to ECE-1 (Emoto and Yanagisawa, 1995; Yanagisawa et al., 2000; Lorenzo et al., 2001). In common with ECE-1, there are also four ECE-2 isoforms possessing a conserved catalytic unit and differing only in their N terminal. The isoforms differ in their localization, and this may be the reason for the variation in the N terminal; ECE-2b was found to be highly expressed in neuroendocrine tissues (brain, pituitary, and adrenal medulla), whereas the other isoforms were in peripheral tissues (Ikeda et al., 2002). In contrast to ECE-1, the optimum pH for ECE-2 activity is acidic (pH 5.5), which would favor an intracellular localization and a potential role under low pH conditions, for example in ischemia. This difference provides an experimental method for distinguishing between the activities of the two enzymes. In agreement, ECE-2 was found to be localized to the acidified environment of vesicles of the secretory pathway in human endothelial cells but was not detected in Weibel-Palade storage granules (Fig. 4; Russell and Davenport, 1999b).

Although ECE-2 efficiently converts Big ET-1, it is important to emphasize that the enzyme is able to process other biologically active peptides. In a study testing ECE-2 for activity against 42 peptides, 10 were processed including conversion of ANGI to ANG1–7 (a vasodilator) and the inactivation of bradykinin. Selective pharmacological inhibition of ECE-2 activity may therefore be difficult to interpret because other vasoactive pathways could be altered in addition to ET-1 (Mzhavia et al., 2003). ECEs have also been reported to beneficially degrade intracellular beta-amyloid produced within the endosomal/lysosomal pathway and autophagosomes (Pacheco-Quinto and Eckman, 2013). Intriguingly, knocking out the ECE-2 gene in mice (section V.B.1) was without any alteration of the phenotype: mice show normal development, life span, and fertility (Yanagisawa et al., 2000). However, a combined double KO, ECE-1(−/−)/ECE-2(−/−) resulted in more severe or additional abnormalities than those in the ECE-1 single knockout embryos, suggesting a significant developmental role for ECE-2. Importantly, whereas levels of ET-1 were reduced, ET-1 was still detectable in the double KO animals, emphasizing the potential for contribution from other pathways to ET-1 synthesis.

#### 4. Peptidic Converting Enzyme/Neutral Endopeptidase Inhibitors: Phosphoramidon.

There are two important aspects of its properties that need to be considered in interpreting the action of phosphoramidon on the endothelin synthetic pathway. Phosphoramidon is not selective for ECE-1, because it was originally developed to inhibit a related enzyme neutral endopeptidase/neprilysin (NEP), and it will also increase levels of vasodilators such as atrial natriuretic peptide (ANP) and inactivate other peptides, including the enkephalins and tachykinins. To confirm ECE activity in a particular cell or tissue preparation in vitro, the action of another NEP inhibitor, thiorphan, must be compared with that of phosphoramidon. Thiorphan does not inhibit ECE-1, and the formation of ET-1 in the presence of this compound is generally accepted as providing evidence of ECE activity. Although using two compounds increases complexity, both reagents have been used in experimental medicine studies to characterize Big ET-1 conversion in vivo (see for example, Plumpton et al., 1995; Webb, 1995; Hand et al., 1999) as well as in animal models. A second complication in interpreting pharmacological assays is that NEP also metabolizes ET-1 to inactive fragments via a two-stage process, opening of the Ser^5^-Leu^6^ bond followed by cleavage at the amino side of Ile^19^ (Skolovsky et al., 1990), and inhibition in tissues derived from kidney, where NEP is particularly abundant, may therefore cause an unanticipated rise in ET-1 (Turner and Tanzawa, 1997).

Finally, phosphoramidon is a protein and does not cross the plasma membrane, therefore its use either in cell-based assays, in vitro or in vivo, will only report on extracellular ECE activity not intracellular ET-1 synthesis (Parnot et al., 1997). This is important because mature ET-1 can be detected together with ECE within Weibel-Palade bodies and secretory vesicles of endothelial cells and it is likely many inhibitors will not alter intracellular synthesis.

#### 5. Small Molecule, Selective Converting Enzyme-1 Inhibitor: PD159790.

Despite the identification of ECE-1 as a rate limiting enzyme in the synthesis of ET-1, there has been much less development of selective small molecule inhibitors that could potentially reduce levels of ET-1 in pathophysiological conditions compared with the considerable effort to discover receptor antagonists. PD159790 was developed to be selective for ECE-1 compared with NEP (Ahn et al., 1998). The compound has been validated experimentally by altering the pH of endothelial cells in culture: at the optimum for ECE-1 activity, pH 6.9, PD159790 inhibited Big-ET-1 conversion but not at the optimum for ECE-2, pH 5.4 (Russell and Davenport, 1999a). In addition, the compound had no effect on the alternative pathway for ET-1 metabolism via chymase generation of ET-1_(1-31)_ (Maguire et al., 2001).

#### 6. Small Molecule Converting Enzyme/Neutral Endopeptidase Inhibitors: Daglutril (SLV306) and SOL1.

No small molecule-selective ECE inhibitor has been tested in the clinic, but compounds have been developed to inhibit both ECE and NEP. Such compounds would be predicted to be antihypertensive by inhibiting the formation of the vasoconstrictor ET-1 as well as increasing vasodilators such as ANP that would normally be inactivated by NEP-mediated metabolism.

The ECE/NEP inhibitor SLV338 has been tested in a number of animal models characterized by elevated levels of ET and therefore potentially tractable to inhibition of ET synthesis, such as in ischemic areas of the brain after stroke. SLV338 was tested for 27 weeks in spontaneously hypertensive, stroke-prone rats. The compound significantly lowered the incidence of stroke and improved survival in a blood pressure-independent manner compared with untreated animals, suggesting a new target for primary stroke prevention (Wengenmayer et al., 2011). Sharkovska et al. (2011) showed that SLV338 was able to preserve kidney function and reduce mortality in a rat model of severe acute ischemic renal failure. In a chronic renal failure model using *N^ω^* -nitro-l-arginine methyl ester (l-NAME) to inhibit nitric oxide synthesis for 4 weeks, which increases levels of ET; SLV338 did not reduce blood pressure, but importantly abolished renal tissue damage (interstitial fibrosis, glomerulosclerosis, and renal arterial remodeling). In a rat model of experimental renovascular hypertension (2-kidney, 1-clip), SLV338 was equally effective as losartan at preventing cardiac remodeling (Kalk et al., 2011). These results are potentially important because there are currently few drugs for the treatment of chronic renal failure.

Daglutril (SLV306) was the first in class, orally active, dual ECE/NEP inhibitor to enter clinical trials (Tabrizchi, 2003; Dickstein et al., 2004; Parvanova et al., 2013), where it was reported to be well tolerated without the disadvantages of side effects of edema and liver toxicity observed with some ET receptor antagonists. SLV306 is a prodrug, hydrolyzed to an active metabolite, KC-12615. In proof of principle studies in volunteers, oral SLV306 significantly increased endogenous plasma ANP concentrations, while reducing the rise in blood pressure after infusion of Big ET-1 (Seed et al., 2012). Initial studies in animal models of diabetes showed that SLV306 was comparable to captopril, an ACE inhibitor, in reducing proteinuria and preventing nephrosclerosis (Thöne-Reinke et al., 2004). In this model, changes in the ET system in the kidney are thought to mediate renal damage by cell proliferation and interstitial inflammation. A limitation of inhibitors of the renin-angiotensin system for treating hypertensive patients with type 2 diabetes is that they become less effective as the condition advances. SLV306 was as effective as captopril in decreasing renal interstitial matrix content as well as protein and albumin excretion (Thöne-Reinke et al., 2004) and could have an additional benefit in targeting the ET pathway to improve renal function. However, in a small randomized, crossover, double-blind, placebo-controlled trial in patients with type 2 diabetic nephropathy, Daglutril on top of losartan did not alter albuminuria that had been predicted from animal studies or renal hemodynamics (Parvanova et al., 2013). This may have been the result of the comparatively short duration of the trial (8 weeks). Importantly, ambulatory blood pressure was reduced, particularly at night when there is a greater risk associated with high blood pressure in this patient group. There was the expected corresponding significant rise in levels of plasma Big ET-1, suggesting inhibition of ECE, but there was no change in proANP that would have been expected as a consequence of NEP inhibition. SLV306 has also been evaluated in a small study of patients with congestive heart failure who underwent right-sided heart catheterization. The results are difficult to interpret because, although pulmonary pressure and right atrial pressure decreased significantly with a maximum effect observed between 6 and 8 hours, there was no clear concentration response relationship between the three doses tested (Dickstein et al., 2004).

SOL1 has been described as a dual ECE/NEP nonpeptide inhibitor, although it is less effective at inhibiting ECE-1 compared with NEP at neutral pH in vitro. However, the compound was effective in inhibiting the Big-ET-1-induced rise in blood pressure in DOCA salt hypertensive rats (10 *μ*mol⋅kg^−1^) (Nelissen et al., 2012) and chronic treatment reduced macrophage infiltration (Lemkens et al., 2012a). SOL1 normalized impaired endothelium-derived hyperpolarizing factor responses in spontaneously hypertensive rats, although there was no effect on blood pressure (Lemkens et al., 2012b).

#### 7. Chymase—A Pathophysiological Synthetic Pathway?

A surprising finding in the double ECE-1/ECE-2 KO mice was the presence of mature ET peptides, suggesting other proteases could synthesize ETs (Yanagisawa et al., 2000). Chymase is a serine protease associated with inflammatory responses, including those in the vasculature, and has been implicated in a range of cardiovascular diseases, particularly after release from mast cells. The enzyme is an alternative synthetic pathway for angiotensin II as well as ET-1. Chymase converts Big ET-1 to an intermediate ET-1_(1–31)_, by cleaving the Tyr^31^–Gly ^32^ bond, which in turn is converted to the mature peptide by NEP (Fecteau et al., 2005; D'Orleans-Juste et al., 2008). Pharmacologically ET-1_(1–31)_ has no selectivity between ET_A_ and ET_B_ receptors in human heart, and vasoconstriction was fully blocked by an ET_A_-selective antagonist, reflecting the predominance of the ET_A_ receptor on vascular smooth muscle (Maguire et al., 2001; Maguire and Davenport, 2004).

In ligand binding assays in human heart, ET-1_(1–31)_ had a similar affinity for both receptor subtypes, but this affinity was approximately 100 times lower than for ET-1. In functional assays in human arteries, ET-1_(1–31)_ caused vasoconstriction (although again less effective than ET-1 itself) that was blocked by an ET_A_ antagonist. This action was unaffected by the ECE inhibitors PD159790 or phosphoramidon, although the appearance of the mature peptide, ET-1, was detected in the bathing medium (Maguire et al., 2001) and may therefore contribute to some of the observed functional response to ET-1_(1-31)_. Interestingly, involvement of chymase activity in conversion of Big ET-1 was confirmed in rat aorta (artery) but not vena cava (vein) (Watts et al., 2007).

How important is the chymase pathway compared with ECE? The results from in vitro pharmacology experiments using human vessels, including coronary arteries, clearly demonstrated the potential for vasoconstriction for the putative chymase product (Maguire et al., 2001). In contrast to humans that have only one chymase, mice express several isoforms, so results are more difficult to interpret in this species. However, mouse mast cell protease 4 is thought to be most similar in activity to human chymase. Levels of ET-1 were reduced by 40% in mouse mast cell protease 4 KOs (Houde et al., 2013), and pressor responses to Big ET-1, but not to ET-1, were reduced (Semaan et al., 2015). Studies on isolated arteries from the kidneys of diabetic mice showed that Big ET-1 vasoconstriction produced by the chymase-dependent pathway measured by chymase inhibition was significantly greater in diabetic compared with control kidneys. It was speculated that this pathway might therefore contribute to end-stage renal disease (Harrison-Bernard et al., 2013).

A potential source of chymase is degranulation of mast cells that may occur under pathophysiological conditions, and a working hypothesis is that this would provide an alternative pathway for ET-1 synthesis in disease. However, paradoxically, injection of ET-1 was found to be toxic to mast cell-deficient mice, suggesting that mast cell degranulation releases proteases that metabolize ET-1 to reduce endogenous ET-1 levels and pathophysiology in vivo (Maurer et al., 2004).

ET-1_(1–31)_ levels were increased to several times higher concentrations, and for longer, than ET-1 in the plasma of patients with acute myocardial infarction (Oka et al., 2014). In contrast, although Big ET-1 levels were elevated in patients with chronic heart failure compared with controls, there was no change in ET-1_(1–31)_ (Leslie et al., 2004). There are currently insufficient data to determine whether changes in ET-1_(1–31)_ are associated with clinical and experimental pathophysiological conditions.

#### 8. Is Dual Vasopeptidase Inhibition a Pharmacologically Flawed Concept?

Although volunteer and clinical studies with SLV306 have provided, to a certain extent, proof of mechanism of action, the results have been disappointing in proving efficacy of ECE/NEP inhibition in heart failure and diabetes. It is possible that inhibition of ECE may switch Big ET-1 conversion to an alternative pathway such as chymase, particularly in disease. In animal models with normal renal function this does not appear to be the case. In a positron emission tomography study, [^18^F]-Big ET rapidly accumulated unchanged in the kidney and there was little evidence of conversion by an alternative pathway (Johnström et al., 2010). The study by Parvanova et al. (2013) was well designed and powered but failed to meet the primary criteria of reducing albuminuria despite promising data from animal studies. The authors speculated that although the reduction in ET produced beneficial effects, the increase in ANP could have increased preglomerular vasodilatation, thus maintaining glomerular perfusion and filtration despite lowered blood pressure. Although the efficacy of a specific inhibitor of ECE-1 has not been tested in the clinic, the clinical evidence is against dual inhibition of ECE/NEP as a therapeutic strategy to inhibit the ET pathway.

### B. Endothelin-2 and Big Endothelin-2

#### 1. Synthesis of Endothelin-2.

In humans and other mammals including monkeys, cats, dogs, and cattle, ET-2 differs from ET-1 by two amino acids, Trp^6^ and Leu^7^. Synthesis of ET-2 is thought to be essentially similar to that of ET-1 (Fig. 2). Preproendothelin-2 is formed after transcription of the *EDN2* gene. After removal of the signal sequence, proendothelin is cleaved by furin, to Big ET-2, a 38-amino acid peptide. Interestingly, ECE-1 and ECE-2 were about 10% less efficient in vitro in converting Big ET-2 to mature peptide compared with conversion of Big ET-1, despite sharing the same scissile bond (Trp^21^-Val^22^) (Emoto and Yanagisawa, 1995). The ET-2 synthetic pathway has been studied in detail in human renal adenocarcinoma cells because these produce high levels of ET-2. Synthesis was inhibited by phosphoramidon and ECE confirmed by sequencing (Yorimitsu et al., 1992, 1995; Shinmi et al., 1993).

In vivo, Gardiner et al. (1992a) showed that Big ET-2 was converted to produce cardiovascular actions (dose-dependent pressor effect, bradycardia, renal and mesenteric vasoconstriction), similar to Big ET-1. All these responses were blocked by phosphoramidon, but the ECE/NEP inhibitor had no effect on the actions of the mature peptide. Similar results were obtained in the anesthetized, ganglion-blocked rat, where intravenous bolus doses of Big ET-2 were compared with Big ET-1 where the cardiovascular actions of both peptides were prevented, as expected, by phosphoramidon but not thiorphan (Mattera et al., 1993).

#### 2. Expression.

The cellular and organ distribution of ET-2 has not been as extensively studied as ET-1, but a pattern is emerging of a more restricted organ distribution. Big ET-2 was detected at higher levels in human plasma than Big ET-1 (Matsumoto et al., 1994), although the concentration of ET-2 (∼0.9 pmol/l) is a one-fifth of that of ET-1 (Davenport and Maguire, 2006). Big ET-2 concentration was ∼2 pmol/l of plasma (Suzuki et al., 1991). The most likely source in the plasma is overspill from endothelial cells: both ET-2 mRNA and Big ET-2 have been detected (Howard et al., 1992; O'Reilly et al., 1993a,b). ET-2 mRNA and/or peptide have been detected in a number of human tissues: vasculature, heart, lung, kidney, intestine, and ovaries (Howard et al., 1992; Marciniak et al., 1992; Plumpton et al., 1993; Karet and Davenport, 1996; Palanisamy et al., 2006). Similarly, in the rat, although all 16 organs examined had measurable mRNA for ET-1, ET-2 mRNA was restricted to heart, lung, ovary, stomach, and intestine (Uchide et al., 1999, 2000). High levels were expressed by regions of the intestine (duodenum, jejunum, ileum, colon, and rectum) as well as the ovary, but not testes (De La Monte et al., 1995; Takizawa et al., 2005). Pituitary glands and medulla oblongata had higher ET-2 mRNA levels than ET-1 (Masuo et al., 2003), suggesting a possible role in these tissues that requires further exploration, although most brain regions had lower or undetectable levels of ET-2 (cerebellum and cerebrum). An alternative strategy using a transgenic mouse line that expresses codon-improved Cre recombinase (iCre) under regulation of the endothelin-2 gene (*edn*2) to map ET-2 expression showed expected expression from previous studies in ovary, stomach, and intestine with a punctate expression in the corneal epithelium, liver, lung, pituitary, uterus, and heart. Interestingly, in the mouse embryo, expression was localized in developing hair follicles and the dermis (Cacioppo et al., 2015).

The tertiary structure of ET-2 was found to be essentially the same as ET-1 by NMR and any differences in pharmacology would be attributable to changes in the characteristics of the two side-chains of Trp^6^ and Leu^7^ (Arvidsson et al., 1998). However, in humans and other mammalian preparations both peptides are equipotent. Studies comparing radiolabeled [^125^I]-ET-2 with [^125^I]-ET-1 binding in cultured rat aortic smooth muscle cells that express ET_A_ receptors found identical affinity constants (*K*_D_ = 0.1 nM) and similar maximum receptor density values (46 and 54 fmol per million cells, respectively). Both ligands had the same association rate constants of 0.01 minute^−1^ and similar slow dissociation rates (Roubert et al., 1991), although Devesly et al. (1990) reported ET-2 dissociated more rapidly at 37°C than ET-1 in Swiss 3T3 fibroblasts that also express ET_A_. In human smooth muscle from aorta and coronary artery that express mainly ET_A_ receptors, saturation binding assays were monophasic, Hill slopes close to unity, and *K*_D_ values were 0.7 nM for [^125^I]-ET-2 and 0.4 nM for [^125^I]-ET-1 (Bacon and Davenport, 1996). Antagonists such as FR139317 were equally effective at competing for [^125^I]-ET-2 as for [^125^I]-ET-1 binding. In agreement, in functional assays, ET-2 had a similar potency as a vasoconstrictor to ET-1 in all human isolated vessels tested (saphenous vein, aorta, coronary, internal mammary artery, and pulmonary arteries (Maguire and Davenport, 1995). ET-2 was also found to be equipotent with ET-1 in animal preparations, for example, rat portal vein (Guimaraes et al., 1992).

#### 3. Vasoactive Intestinal Contractor and Big Vasoactive Intestinal Contractor.

In rodents, ET-2 differs from ET-1 by three amino acids (Fig. 1; Asn^4^, Trp^6^, and Leu^7^) (Bloch et al., 1991) and is also called vasoactive intestinal contractor (VIC) because it was originally identified by its ability to contract isolated mouse ileum (Ishida et al., 1989). The synthetic pathway is the same as ET-2: preproVIC, a 175 amino acid precursor protein, via a 38-amino acid Big VIC, which in turn is cleaved to VIC and a C-terminal fragment, which is sensitive to phosphoramidon inhibition (Saida et al., 1989, 2000, 2002; Yorimitsu et al., 1992).

These amino acid substitutions have no effect on the pharmacology of VIC, with the peptide having the same affinity for both receptor subtypes. [^125^I]-VIC had a similar autoradiographical distribution in human, porcine, and rat tissues compared with [^125^I]-ET-1 and [^125^I]-ET-2 (Davenport and Morton, 1991; Davenport et al., 1991). No differences were found in the ability of unlabeled VIC, ET-1, ET-2, or ET-3 to compete for [^125^I]-VIC in ligand binding assays or in vasoconstrictor responses in the guinea pig longitudinal smooth muscle (Yoshinaga et al., 1992). In other ET_B_ (Cozza et el., 1992; Lin and Lee, 1992) or predominantly ET_A_ preparations, VIC was equipotent with ET-1 and ET-2 (Douglas and Hiley, 1990; Schoeffter and Randriantsoa, 1993). VIC had a lower potency than ET-1 in cultured human smooth muscle cells (Iwashima et al., 1997).

#### 4. Distinct Physiologic/Pathophysiological Role for Endothelin-2.

Although the pharmacology of ET-2 is similar to ET-1 and both peptides may be released from the same cells, such as the endothelium, and exist in the same tissue compartments, there is increasing evidence that ET-2 does not simply duplicate ET-1 but has a distinct physiologic and potentially pathophysiological role. This difference may be achieved by a change in levels of gene expression and through differences in synthesis.

Deletion of the ET-2 gene in mice produces a different phenotype to that affected by disrupting the ET_A_/ET-1 or the ET_B_/ET-3 pathways. The ET-2 phenotype was characterized by severe growth retardation, hypoglycemia, ketonemia, and increased levels of starvation-induced genes as well as severe hypothermia, but surprisingly intestinal function was normal (Chang et al., 2013; section V.A.3). After selective deletion of the ET-2 gene in epithelial cells, mice showed no abnormalities in growth and survival, but major changes were observed in lung morphology leading to increased levels of carbon dioxide (hypercapnia) and a deficiency of oxygen (hypoxemia) in the blood. It is not yet clear whether this phenotype is a consequence of internal starvation as a consequence of reduction in ET-2 expression or whether ET-2 may have a critical function in the pulmonary system. ET-2 mRNA localized to epithelial cells, whereas receptor mRNA was mainly present in mesenchyme, suggesting a paracrine function for ET-2 (Chang et al., 2013). PAH remains the major clinical indication for ET antagonists (Liu et al., 2013), where the importance of dysregulation of vascular cell growth is increasingly recognized. Selective deletion of ET-2 may provide new clues to the progression of this disease and the role of ET-2 in PAH warrants further investigation.

Evidence is emerging that ET-2 plays a key role in ovarian physiology, with ET-2-mediated contraction proposed as a final signal facilitating ovulation (Meidan and Levy 2007; Ko et al., 2012; Ling et al., 2013). ET-2 expression is higher than ET-1 in ovaries (Uchide et al., 1999). ECE-1 and ET-2 were found to be transiently expressed in rat ovaries during ovulation (Ko et al., 2006), and in mice, induced superovulation resulted in a surge of ET-2 mRNA expression after 11 hours that coincided with the time of follicular rupture and then subsequently declined (Palanisamy et al., 2006). The precise mechanism of rupture and ovulation are undetermined, but ET-2 caused contraction of the smooth muscle layer surrounding each follicle (Ko et al., 2006). In support, tezosentan (a mixed ET_A_/ET_B_ receptor antagonist), reduced ovulation and resulted in mature unruptured follicles despite gonadotropin superovulation. Although differences are reported between species in the action of ET-2 in ovarian physiology (see Ling et al., 2013), overall the results suggest ET-2 has both a spatial and temporal role in mediating follicular rupture, without which normal ovulation cannot proceed. Further studies on ET-2 signaling pathways in ovulation are needed and may lead to the identification of new drug targets for associated with infertility such as Luteinized Unruptured Follicle Syndrome.

ET-2 also has a distinct chemokine role, functioning as a chemoattractant for neutrophils at low levels, although inhibition of migration occurs at high concentrations (Elferink and de Koster, 1996). The peptide was also found to be as effective as the inflammatory chemokine CCL2 in stimulating chemotaxis in macrophages, mediated via ET_B_ receptors through the MAPK pathway (Yen et al., 1997; Grimshaw et al., 2002b). ET-1 has been shown to affect many stages of cancer development, including proliferation, angiogenesis, migration, invasion, and apoptosis (Grant et al., 2003; Bhalla et al., 2009), but there is increasing evidence for a role for ET-2. The peptide has been isolated from renal adenocarcinoma cell lines (Ohkubo et al., 1990). ET-2 is absent from breast tissue, but under hypoxic conditions, cultured cells from HTH-K mice human breast tumor cell lines show enhanced expression of ET-2, ET_A_, and ET_B_ but not ET-1 and ET-3 (Grimshaw et al., 2002a,b). ET-2 may be the principle isoform increased in hypoxic tumors with increased ET-2 in cancers such as those found in the skin, where expression is three times higher in basal cell carcinoma compared with normal skin (Tanese et al., 2010). Further studies are required to determine whether the ET-2 pathway is a potential target in cancer therapy (Grimshaw et al., 2004).

### C. Endothelin-3, the Receptor Subtype Selective Isoform and Big Endothelin-3

ET-3 is the only endogenous isoform that, at physiologic concentrations, can distinguish between the two receptor subtypes, because it has the same affinity at ET_B_ receptors as ET-1 and ET-2 but has much lower or little affinity for ET_A_ than the other isoforms (Fig. 2). It was an early discovery in the field by Matsumoto et al. (1989) that ET-3 was the most abundant ET peptide in rat brain, mainly localized to neurons and glia of the neostriatum, hypothalamic nuclei, hippocampus, and Purkinje cells of the cerebellum and medulla oblongata (Giaid et al., 1991). This led to the proposal that ET-3 was the "brain" endothelin peptide, particularly because the ET_B_ subtype comprises ∼90% of the ET receptors in human normal cerebral cortex (Harland et al., 1995) (section III.C).

KO mice also emphasize the importance of the ET-3/ET_B_ pathway in the development of the enteric nervous system (Kurihara et al., 1999), a complex network of neurons and glia in the gut controlling intestinal motility, secretion, and blood flow. A mutation in ET_B_ is a common cause of Hirschsprung disease (section V.B.3), in which enteric nervous system precursors and neural crest-derived epidermal melanoblasts fail to colonize the intestine and skin.

#### 1. Endothelin-3 Synthesis and Kell.

Controversy remains concerning the precise contribution of ECE-1 to ET-3 synthesis. ECE-1-deficient mice recapitulated the combined phenotype of both ET-1/ET_A_- and ET-3/ET_B_-deficient mice. Tissue ET-3 levels were lower than in wild-type animals, suggesting that ECE-1 was an important converting enzyme for both Big ET-1 and Big ET-3 in vivo, at least in mice (Yanagisawa et al., 2000). However, Big ET-3 has been reported to be poorly converted by ECE-1 when tested as the purified enzyme or in isolated tissue preparations (D'Orléans-Juste et al., 1991; Telemaque and D'Orléans-Juste, 1991; Shimada et al., 1994; Turner and Murphy, 1996), owing to differences in the scissile bond Trp^21^-Ile^21^ in Big-ET-3 compared with Trp^21^-Val^21^ in Big ET-1 and in the C-terminal sequence. However, Big ET-3 in vivo produces vasoconstriction, for example, in the chronically instrumented rat (called proendothelin-3 by Gardiner et al., 1992a,b), suggesting the existence of further converting enzymes. The Kell blood group comprises 20 different antigens borne by the protein Kell, a 93-kDa membrane bound glycoprotein expressed by erythrocytes with homology to the type II membrane-bound zinc metalloprotease family that includes ECE-1 and ECE-2 (Clapéron et al., 2005). A recombinant, nonphysiologic form of Kell has been shown to efficiently cleave Big ET-3, suggesting the existence of a specific converting enzyme for the synthesis of ET-3 (Lee et al., 1999).

Clapéron et al. (2005) expressed the Kell K2 antigen under more representative physiologic conditions in erythroid and nonerythroid cells lines. Kell K2 efficiently cleaved Big ET-1 as well as Big ET-3 (Big ET-2 was not tested), but unlike ECE-1, the enzyme was inhibited efficiently by thiorphan as well as phosphoramidon. It was not selective for ET synthesis because it also cleaved tachykinins. The relative contributions of Kell versus ECE-1/ECE-2 to the synthesis of mature peptides remain unclear but may explain the disappointing results of targeting the latter (see section V.B.1), and further studies are required to comprehensively delineate the synthetic pathways contributing to the production of the mature endothelin peptides.

#### 2. Expression.

ET-3 (typically ∼0.3 pmol/l) and an order of magnitude higher level of Big ET-3 (∼6 pmol/l) are detectable in human plasma (Matsumoto et al., 1994). Changes associated with disease have not been extensively investigated, but concentrations of Big ET-3 increased significantly in hemodialysis patients (together with Big ET-1 and Big ET-2) but with only a moderate rise in corresponding active peptides (Miyauchi et al., 2012). Human endothelial cells do not synthesize ET-3, but one possible source of circulating Big ET-3 could be the adrenal gland, where this peptide has been visualized in secretory cells of the medulla using selective antisera, although mature ET-3 could not be detected (Davenport et al., 1996). If released, further processing of Big ET-3 could occur within the vasculature by smooth muscle cell ECE (Davenport et al., 1998). ET-3 has been detected in other tissues, including heart (Plumpton et al., 1993), endometrium (O'Reilly et al., 1992; Cameron et al., 1993) using HPLC fractionation combined with radioimmunoassay, brain, and pituitary using sandwich enzyme-linked immunosorbent assay—interestingly the pituitary was stated (but not shown) to have higher levels of ET-3 than ET-1 (Takahashi et al., 1991). In other species, high concentrations of immunoreactive ET-3 were found in the intestine, lung, pituitary gland, and brain (greater than 100 pg/g wet tissue) in rats; in agreement with humans, only pituitary gland expressed higher levels than ET-1 (Matsumoto et al., 1989). Northern analysis showed that preproET-3 mRNA was expressed in the eye, submandibular gland, brain, kidney, jejunum, stomach, and spleen (Shiba et al., 1992). Big ET-3 was also localized by immunocytochemistry (with Big ET-1 and Big ET-2) to mast cells and macrophages in the rat gastrointestinal tract (Liu et al., 1998). Because ET-3 does not activate ET_A_ receptors at physiologic concentrations, the expression should correlate with higher densities of ET_B_ receptor—and this is true to a certain extent for the brain and pituitary (compare in Fig. 7).

### D. Snake Venom Peptides, the Sarafotoxins

The sarafotoxins are a family of four isoforms (S6a, S6b, S6c, S6d; Fig. 2) with a high degree of sequence similarity to ET-1, identified from the venom of the snake *Atractaspis engaddensis* (Kloog et al., 1988; Takasaki et al., 1988). The main differences are in residues 5, 6, 7, and 17, which are Asp, Met, Thr, and Gln. The venoms evolved to immobilize mammalian prey and can be fatal in humans. Symptoms are a rapid rise in blood pressure consistent with systemic vasoconstriction, as observed with ET-1 and changes in electrocardiogram consistent with coronary vasoconstriction or direct inotropic actions on the heart accompanied by changes in the electrocardiogram associated with atrioventricular block and cardiac arrest (Kurnik et al., 1999). The most widely studied are S6b and S6c. Sarafotoxin 6b has similar affinities for both endothelin receptors with a profile similar to ET-1/ET-2, although less potent, whereas S6c is used as a moderately selective ET_B_ agonist (Maguire et al., 1994, 1996; Maguire and Davenport, 1999). A fifth isoform, bibrotoxin, was isolated from the venom of the burrowing asp *Atractaspis bibroni* with an amino acid sequence similar to S6b but with the substitution of Ala^4^ instead of Lys^4^ and similar pharmacology (Becker et al., 1993).

## III. Receptor Structure, Distribution, and Function

ETs activate two receptor subtypes (Davenport, 2002), ET_A_ (Arai et al., 1990) and ET_B_ (Sakurai et al., 1990), that belong to Class 1 (Family A or rhodopsin-like) of the G protein-coupled, 7 transmembrane-spanning domains receptors (GPCR). Although ET receptor-like genes are found outside the vertebrates in the cephalochordate amphioxus, suggesting a long lineage in eukaryote evolution, genes encoding peptides are only present among vertebrates: jawless vertebrates (lampreys and hagfishes), cartilaginous vertebrates (sharks, rays, and chimaeras), and bony vertebrates (ray-finned fishes and lobe-finned vertebrates including tetrapods). It is therefore likely that the receptor-ligand signaling pathway is a vertebrate innovation consistent with the role of the development and regulation of the cardiovascular system (Braasch and Schartl, 2014).

The sequencing of the human genome is virtually complete and most if not all genes encoding GPCRs have been identified (Foord et al., 2005; Davenport et al., 2013). The receptors most closely related in structure to ET_A_ and ET_B_ are GPR37 (endothelin receptor type B-like or Parkin-associated endothelin receptor-like receptor) and GPR37L1. Neither of these receptors was activated by any of approximately 20 ET peptides, Big ET precursors, or known ET receptor ligands (such as BQ123 and BQ3020) that were tested at high concentration. Two other peptides, prosaptide and prosaposin, have been identified as cognate ligands for GPR37 and GPR37L1 (Meyer et al., 2013). In addition, none of the ET ligands had any activity against the remaining ∼80 GPCRs in the screen that are currently classified as orphans, because their endogenous ligand is not yet known.

Studies continue to be published suggesting the existence of further ET receptor subtypes: for example, ET_B_ isoforms were proposed with ET_B1_ present on endothelial cells and ET_B2_ on smooth muscle cells, but the evidence is against this. In competition binding assays, ET_B_ receptors expressed by human isolated endothelial compared with smooth muscle cells in culture could not be distinguished by a range of ET ligands (Flynn et al., 1998). Radiolabeled ET_B_ ligands were always found to bind with a single affinity and Hill slopes close unity in saturation binding assays (Molenaar et al., 1992, 1993; Nambi et al., 1994) and to produce monophasic curves in competition binding assays versus [^125^I]-ET-1 in human native (Peter and Davenport 1995a,b; Russell and Davenport, 1996) or recombinant ET_B_ receptors (Nambi et al., 1994; Reynolds et al., 1995). In global ET_B_ receptor knockout mice both the direct constrictor (smooth muscle) and indirect vasodilator (endothelium) responses to the ET_B_ agonist sarafotoxin 6c were abolished (Mizuguchi et al., 1997). Current evidence only supports the existence of two subtypes, ET_A_ and ET_B_, according to NC-IUPHAR nomenclature (Davenport, 2002). No further Family A GPCRs have been identified that might bind ET peptides.

### A. Endothelin A Receptors

#### 1. Structure.

**Fig. 5.**
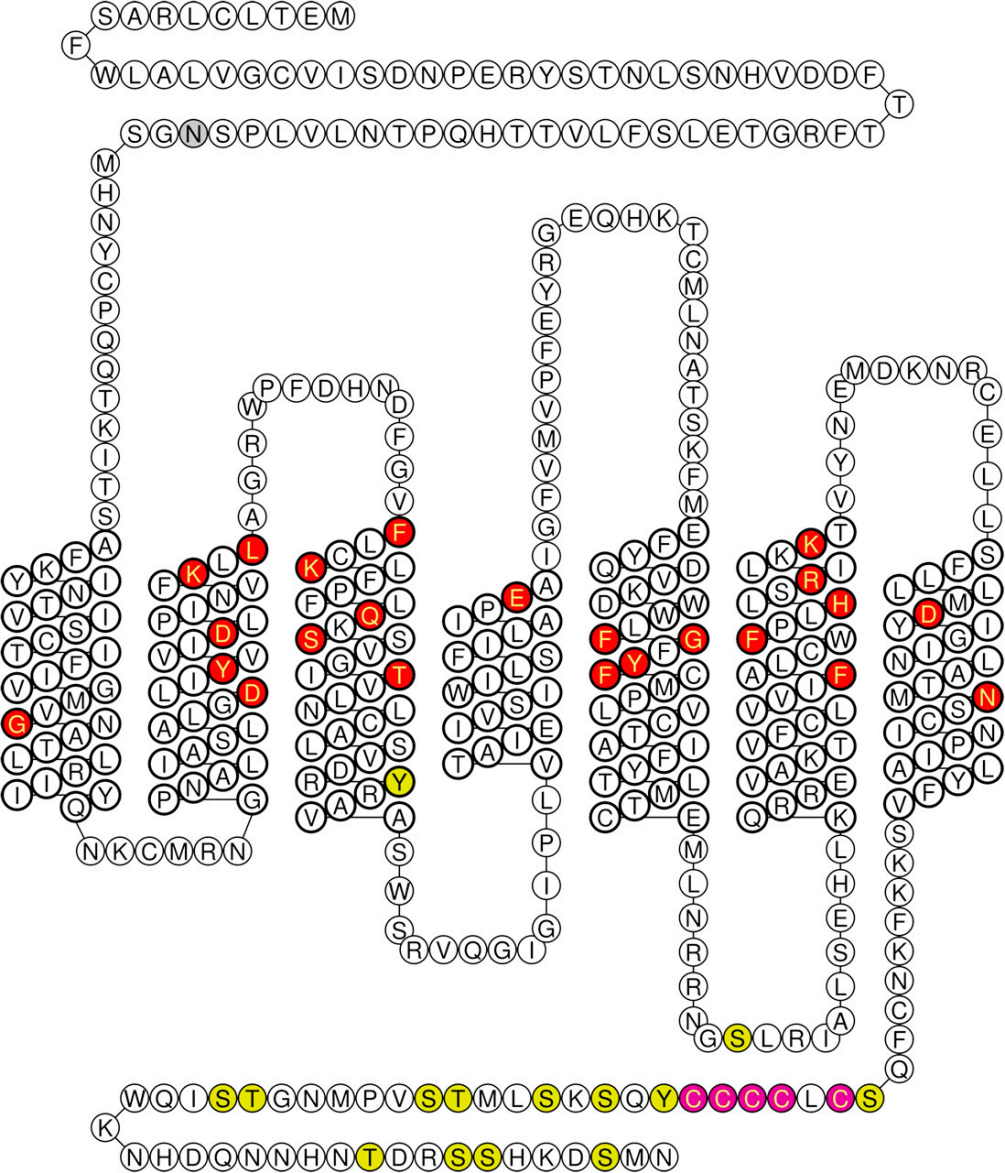
Schematic diagram of the human ET_A_ receptor. Experimental mutations altering receptor function are shown in red. Potential sites for translational modifications are shown as phosphorylation (yellow), glycosylation (gray), and palmitoylation (purple). Diagrams generated using http://tools.gpcr.org/ (Isberg et al., 2014).

The human ET_A_ receptor has 63% sequence similarity with the human ET_B_ receptor over a 420-residue match length. The amino acid sequences of human ET_A_ differ from other species, for example by 9% from rat ET_A_ and by 12% for the ET_B_. The ET_A_ gene spans more than 40 kilobases and contains eight exons and seven introns. It is located on chromosome 4 and encodes a 427-amino acid protein (Hosoda et al., 1992). The amino acid sequence of the human ET_A_ receptor is shown in Fig. 5. The receptor sequence displays a high degree of conservation between human and animal species, including pig, dog, sheep (95%), rat (93%), and mouse (92%). The main differences are in the N terminus, rather than within the transmembrane domains that contribute to binding, and reflect that ET-1 structure is conserved across mammalian species. Figure 5 shows the amino acids that have been mutated and reported to alter the binding of ET ligands. The choice of amino acids to mutate is determined usually by modeling the three-dimensional structure and identifying amino acids that are likely to have groups associated with the binding of ligand to the receptor. For example, an extensive study mutating 18 amino acids identified that Gly^97^, Lys^l40^, Lys^159^, Gln^165^, and Phe^315^, located in transmembrane regions 1, 2, 3, 3, and 6, respectively, reduced binding of radiolabeled ET-1. Interestingly mutation of Tyr^263^, Arg^326^, and Asp^351^ preserved ET-1 binding but reduced that of the antagonist bosentan. All of these three residues are conserved in the ET_B_ subtype, consistent with bosentan displaying little selectivity for either ET_A_ versus ET_B_.

Posttranslational modifications, phosphorylation, palmitoylation, and glycosylation can further modulate the function of GPCRs (Fig. 5). Mutation of a cluster of five Cys residues present in the cytoplasmic tail did not alter binding but prevented palmitoylation of the receptor, resulting in a failure of ET-1 to stimulate the transient increase in the cytoplasmic calcium. Posttranslational modification by palmitoylation would seem to be essential for functional activity of ET_A_ (Horstmeyer et al., 1996). Truncation of the entire N terminus that included a possible glycosylation site abolished binding activity, but it is unclear whether this is the result of a structural change or whether glycosylation is important (Hashido et al., 1992). Phosphorylation is crucial for ligand induced desensitization in ET_B_ receptors, with rapid deactivation after ET-1 stimulation and most activity lost within 5 minutes. In contrast, ET_A_ did not undergo ligand-induced phosphorylation and continued to signal with little reduction in activity after 5 minutes, consistent with long-lasting constrictor activity (Cramer et al., 1997).

#### 2. Receptors Mutations.

ET_A_ receptor knockout mice bear morphologic abnormalities nearly identical to ET-1 and ECE-1 knockout mice. Homozygous mice died at birth of respiratory failure secondary to severe craniofacial abnormalities (Hosoda et al., 1994; section V.B.2). ET_A_ receptors are essential for development, and mutations will be lethal or detrimental. Deletions in the ET_A_ receptor in mice mimic the human conditions collectively termed CATCH 22 or velocardiofacial syndrome. These include severe craniofacial deformities and defects in the cardiovascular outflow tract. Great vessel malformations highly similar to those seen in neural crest-ablated chick embryos and human congenital cardiac defect also occur.

#### 3. Splice Variants.

At least three alternatively spliced ET_A_ receptor transcripts have been reported in human tissues, including aorta, heart atria, and lung. Deletions of exon 3 (producing a protein with two membrane-spanning domains), exon 4 (producing a protein with three membrane-spanning domains), and exon 3 plus exon 4 (producing a protein lacking the third and fourth domain) have been identified, but these did not bind ET-1 when expressed in cells (Miyamoto et al., 1996; Bourgeois et al., 1997; Zhang et al., 1998). The significance of alternative splicing of ET_A_ mRNA is unclear but could reduce the abundance of active receptor and reduce functions such as contractility. For example, mRNA encoding the predicted truncated receptor with the deletion of exon 3 and 4 was more abundant in human melanoma cell lines and melanoma tissue than the wild type (Zhang et al., 1998).

### B. Endothelin B Receptors

#### 1. Structure.

**Fig. 6.**
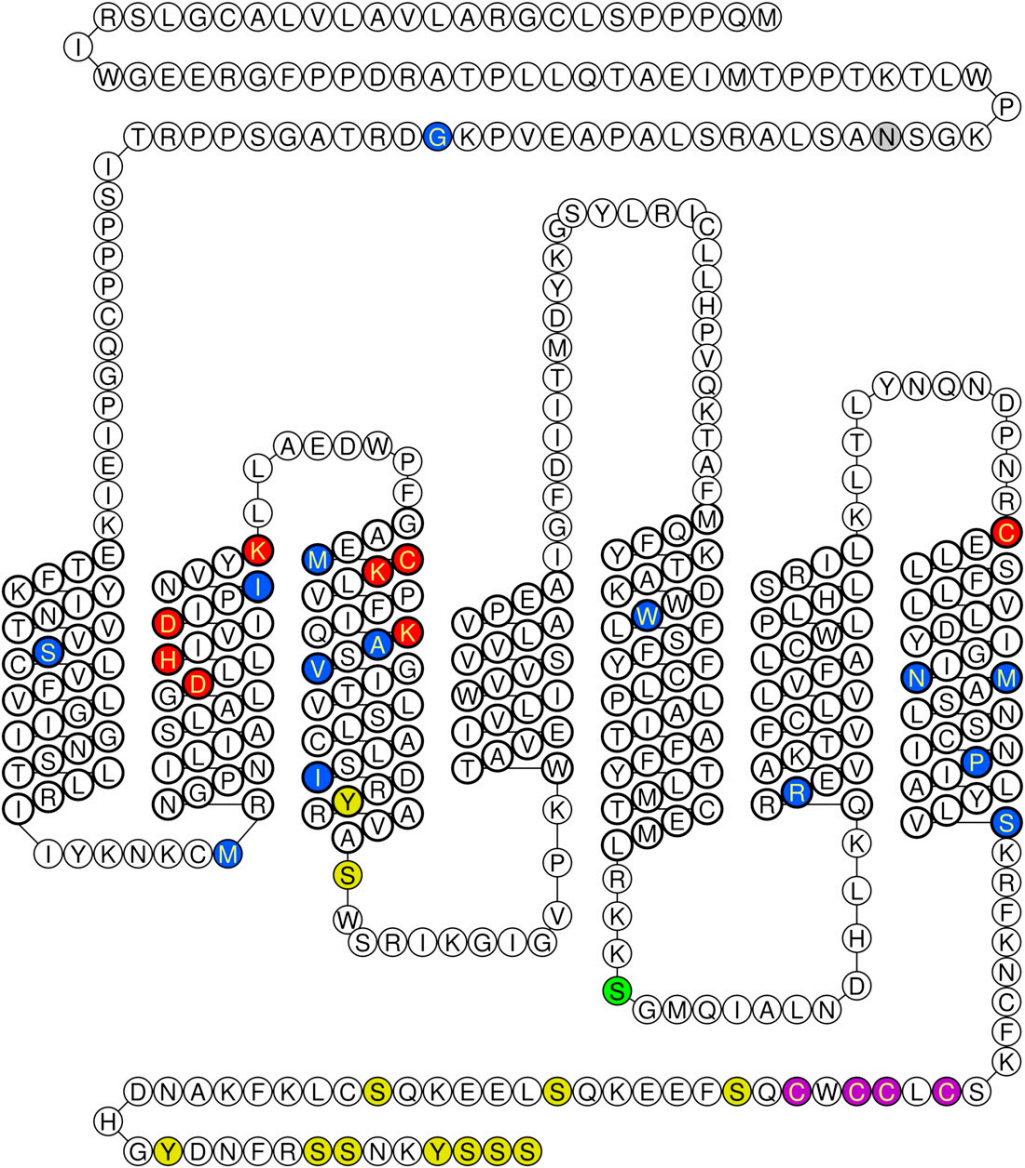
Schematic diagram of the human ET_B_ receptor. Naturally occurring mutation reported in patients with Hirschsprung disease (blue), experimental mutations altering receptor function (red). Potential sites for translational modifications as shown as phosphorylation (yellow), glycosylation (gray), and palmitoylation (purple). Green indicates both a phosphoraylation and site experimentally mutated. Diagrams generated using http://tools.gpcr.org/ (Isberg et al., 2014).

Human ET_B_ gene (*EDNRB*) comprises 7 exons and 6 introns, is located on chromosome 13, and encodes a 442-amino acid protein. The ET_B_ receptor has an unusually long N terminus (Fig. 6) that can be cleaved by a metalloprotease to remove the first 64 amino acids while still retaining ET-1 binding capacity. It is thought that amino acids associated with the first, second, third and seventh transmembrane spanning domains and associated extracellular loops are mainly involved in ligand binding where interestingly most of the naturally occurring or experimental mutations that affect ET ligand binding and ligand receptor selectivity coincide. The carboxyl terminus, as with other GPCRs, is important for intracellular signaling.

Like ET_A_ receptors, ET_B_ receptors have a surprising number of potential post-translational modifications (Fig. 6). There are 13 phosphorylation sites comprising Tyr, Ser, Cys residues mainly located in the C terminus. There is a potential glycosylation site at Asn^59^ in the N terminus but no evidence yet that this is important for binding, although in other GPCRs at least one glycosylation site is often critical for receptor expression at the cell membrane and for function (Okamoto et al., 1998). In addition, there are four sites for possible palmitoylation. The clustering of Cys residues is conserved across many GPCRs, and the degree of C-terminal palmitoylation may act as a selector for coupling with different G proteins and different pathways of intracellular signaling (Stannard et al., 2003a,b). For example, ET mitogenic activity that stimulates extracellular signal-regulated kinases (ERK/MAP kinases) critically depends on palmitoylation (but not on phosphorylation) of ET receptors (Cramer et al., 2001). Okamoto et al. (1998) showed that substitution of three residues Cys^402^, Cys^403^, or Cys^405^ with Ser gave an unpalmitoylated mutant that retained binding and surface expression but did not couple to G proteins, suggesting a critical role in signaling.

#### 2. Receptor Mutations.

Homozygote ET_B_ knockout mice exhibit a different and nonoverlapping phenotype to ET_A_-deficient animals with survival for up to 8 weeks. The enteric nervous system precursors and neural crest-derived epidermal melanoblasts fail to colonize the intestine, causing an aganglionic megacolon and a pigmentary disorder in their skin. ET-3 knockouts display an identical phenotype (section V.B.3; Kurihara et al., 1999). This phenotype is similar to Hirschsprung disease (estimated at 1/5000 live births), a multigenetic disorder, where one of the causative genes includes mutations in ET_B_ receptor expression (Tanaka et al., 1998). Hirschsprung disease can also be associated with Waardenburg syndrome (where in addition to colonic aganglionosis the patients have sensorineural hearing loss, hypopigmentation of skin and hair, and pigmentary disturbances of the irides) that is also linked to mutations in the ET_B_ and ET-3 genes. The effect of some ET_B_ receptor mutations in Hirschsprung disease have been tested experimentally. For example, A183G, W276C, R319W, M374I, and P383L had reduced intracellular signaling and reduced receptor density resulting in a loss of function, although all mutants bound ET-1 with high affinity (Fig. 6; Abe et al., 2000), suggesting ET agonists such as IRL 1620 could rescue function, whereas others such as the C109R mutation failed to translocate into the plasma membrane and had low ET-1 affinity.

A naturally occurring 301-base pair deletion of the ET_B_ gene resulting in a lack of ET_B_ expression, elevation of plasma ET levels, and aganglionic megacolon was identified in "spotting lethal" rats. ET_B_ deficiency caused early onset of renal impairment characterized by reduced sodium excretion and decreased glomerular filtration rate (Hocher et al., 2001; Taylor et al., 2003). A missense mutation in the ET_B_ gene is also associated with Lethal White Foal Syndrome, an equine version of Hirschsprung disease (Metallinos et al., 1998).

#### 3. Splice Variants.

Alternative splice variants of ET_B_ receptors have been reported in humans but they do not seem as important as for other transmitters such as dopamine. Elshourbagy et al. (1996) identified a variant where substitution of 42 amino acids of the intracellular carboxy terminus of the ET_B_ receptor was replaced with an alternative 36-residue sequence, resulted in a receptor retaining binding but lacking any potential palmitoylation sites so that ET-stimulated inositol phosphate accumulation was abolished. Messenger RNA encoding the variant was not particularly abundant, except in skeletal muscle where it represented more than 40% of the total mRNA. Shyamala et al. (1994) described a variant encoding a 10 amino acid increase in the length of the second cytoplasmic domain that exhibited limited distribution in heart, lung, brain, and placenta, but there was no change in binding characteristics or downstream signaling, and therefore the physiologic or pathophysiological significance is unclear. Tanoue et al. (2002) described a mutant ET_B_ gene associated with Hirschsprung disease where the transcript lacked a 134-base pair nucleotide sequence corresponding to exon 5 that may not have been translated or rapidly degraded as a possible cause of the disease. An alternative transcript of the rat ET_B_ in brain has also been reported (Cheng et al., 1993).

### C. Distribution of Endothelin Receptors

**Fig. 7.**
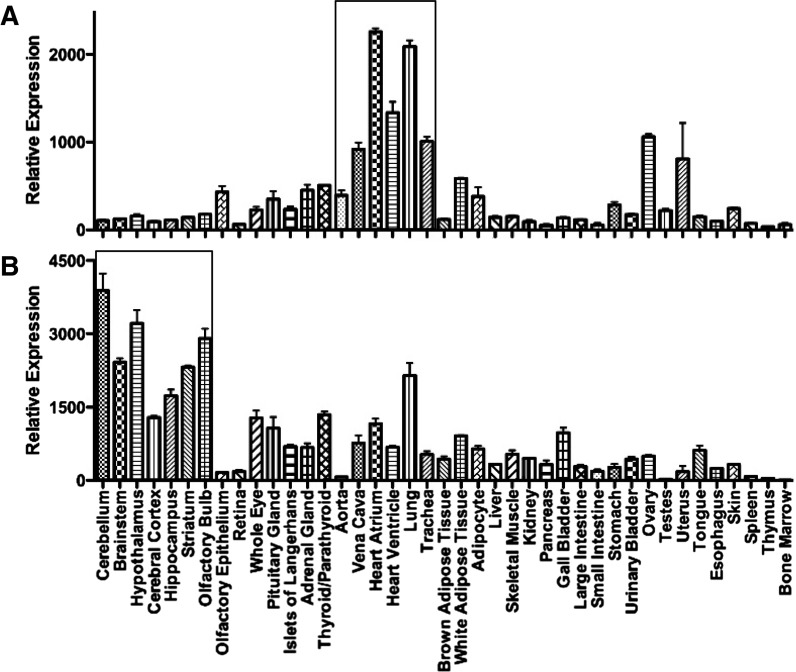
Relative expression of mRNA encoding ET_A_ (*Ednra*) or ET_B_ (*Ednrb*) receptors in 41 adult tissues. (Graphs constructed using data from Regard et al., 2008).

The distribution of mRNA encoding the two receptors is shown in Fig. 7, measured in adult mouse tissues, a species widely used for cell-specific disruption of the ET genes. The pattern emphasizes that ET receptor mRNA is likely to be detected in all tissues or organs receiving a blood supply, reflecting the ubiquitous expression of ET_A_ on vascular smooth muscle and ET_B_ on endothelial cells. Highest relative expression of ET_A_ mRNA is associated with the heart and lungs with relatively low expression in the CNS. ET_A_ mRNA was the fifth most abundant GPCR in heart atria and 11th in ventricle, emphasizing the importance of the ET pathway in the cardiovascular system (Regard et al., 2008). By radioligand binding, the brain regions measured have high expression of ET_B_ receptors and in the periphery the lung is also a particularly ET_B_ rich tissue.

**Fig. 8.**
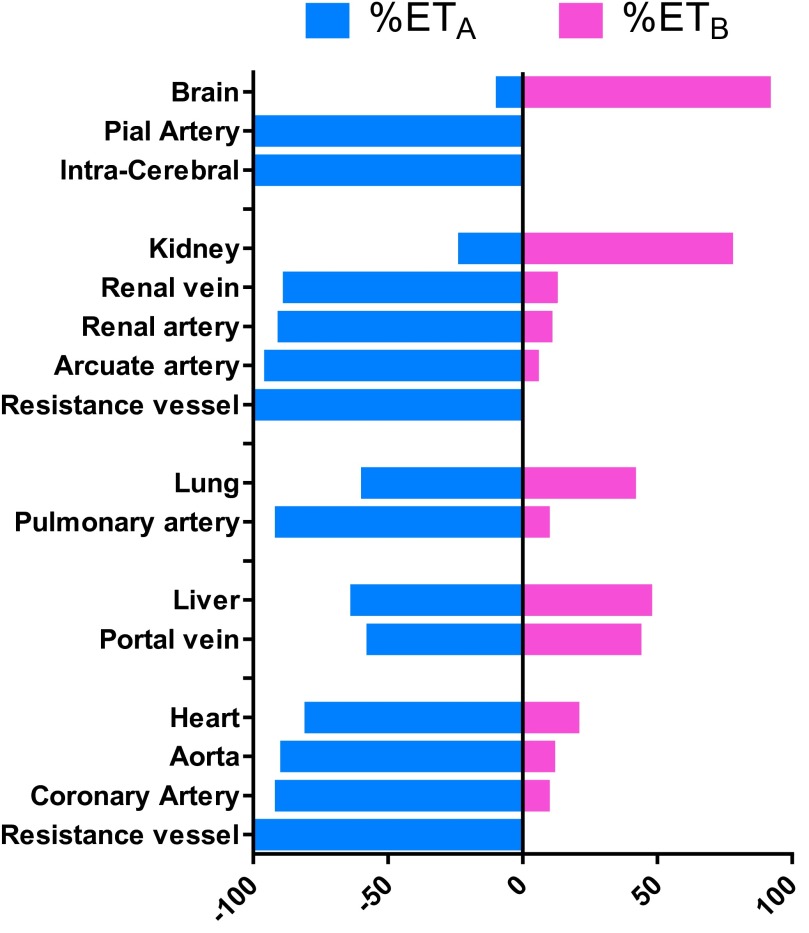
Ratio of ET_A_ to ET_B_ densities measured by saturation binding assays in the human brain, kidney, lung, liver, and heart and smooth muscle layer of the vessels from each organ.

The relative ratio of the two subtypes, measured by competition binding in human tissues, is shown in Fig. 8. The human brain contains a high density of ET receptors (see Schinelli, 2006 for review), with ET_B_ accounting for 90% of total ET receptors in the cerebral cortex (Harland et al., 1995), localized to neuronal regions, particularly levels III and IV. The small proportion of ET_A_ receptors detected were largely restricted to the smooth muscle of the cerebral vasculature (pial arteries and intracerebral vessels) and leptomeninges, with much lower but detectable levels in the gray and white matter. ET_B_ receptors were not detected in the vascular structures or leptomeninges. Functional studies in rat cerebellum suggested glial cells expressed the ET_B_ subtype, whereas some responses of neurons were ET_A_ mediated (Davenport and Morton, 1991; Morton and Davenport, 1992). Smooth muscle cells of the small pial arteries and arterioles that penetrate into the human brain only express ET_A_ receptors (Adner et al., 1994; Yu et al., 1995; Lucas et al., 1996; Harland et al., 1995, 1998; Pierre and Davenport, 1995, 1998a,b, 1999) that play a major role in the maintenance of cerebral blood flow. ET-1 potently constricts brain vessels, including cerebral (Adner et al., 1994) and pial arteries (Hardebo et al., 1989; Thorin et al., 1998; Pierre and Davenport, 1998b, 1999). The latter are particularly sensitive to ET-1 and provide the therapeutic rationale for blocking the actions of the peptide, which is thought to be a mediator of cerebrovascular disorders including delayed vasospasm associated with subarachnoid hemorrhage and stroke. In contrast to the periphery, both ligand binding (Yamaga et al., 1995) and functional studies suggest that human brain endothelial cells isolated from capillaries (diameter <10 *µ*m) that form the blood-brain barrier and from larger microvessels express ET_A_ receptors linked to phospholipase C and IP_3_ accumulation (Stanimirovic et al., 1994; Spatz et al., 1997) possibly functioning to increase capillary permeability, leading to edema (Purkiss et al., 1994). These studies used a comprehensive range of agonists and antagonists to show ET_A_-mediated responses, but this intriguing finding has not been explored further. Interestingly, there are numerous reports of upregulation of expression of ET_B_ receptors in cerebrovascular smooth muscle in rat models of, for example, global ischemic stroke (Johansson et al., 2012), subarachnoid hemorrhage (Vikman et al., 2006), or cerebrovascular remodeling associated with diabetes (Kelly-Cobbs et al., 2011), although effectiveness of ET_A_ or dual receptor antagonists in human cerebrovascular disease appears to be limited.

In human peripheral tissue, the lungs have the highest density of ET receptors (∼9600 fmol/g protein) measured to date compared with other peripheral tissues and even higher than the brain (∼5000 fmol/g protein) (Henry et al., 1990; McKay et al., 1991; Marciniak et al., 1992; Knott et al., 1995; Russell and Davenport, 1996). ET_A_ receptors predominate on smooth muscle of pulmonary arteries and resistance vessels where the subtype mediates vasoconstriction (Hay et al., 1993; Maguire and Davenport, 1995), although McCulloch et al. (1996) suggested a contribution from ET_B_ receptors in resistance vessels. The majority of ET_B_ receptors in the lungs are present on the endothelium, but this receptor is also localized to airway smooth muscle and mediates bronchoconstriction (Adner et al., 1996; Takahashi et al., 1997; Hay et al., 1998), although Fukuroda et al. (1994a) detected an ET_A_ component in isolated human bronchi responses to ET-1. Lower levels of receptors are detectable in parenchyma, airway submucosal glands, and small conduit arteries (Russell and Davenport, 1995). Plasma levels of ET-1 are increased in PAH and correlate with the severity of disease, as well as its prognosis (Rubens et al., 2001). ET-1 levels are also increased in explanted lungs (Giaid et al., 1993), as is the density of both subtypes in distal pulmonary arteries of patients with PAH (Davie et al., 2002). In PAH, this increase in ET-1 is thought to cause sustained vasoconstriction and stimulates cellular proliferation, cell migration, fibrosis, and hypertrophy (see Vachiery and Davenport, 2009).

In human heart, both receptor subtypes are expressed by the myocardium of atria and ventricles (Bax et al., 1993; Molenaar et al., 1992, 1993; Peter and Davenport, 1995a,b, 1996a; Pönicke et al., 1998) and septum, although the ET_A_ receptor predominates. In myocytes ET_A_ comprise 90% of the receptors. In contrast ET_B_ receptors are more abundant in the atrioventricular conducting system (Molenaar et al., 1993). ET-1 is a potent positive inotropic agent (Davenport et al., 1989; Moravec et al., 1989). In vivo the endogenous ligand ET-1 has a tonic positive inotropic effect in normal subjects that can be blocked by BQ123 and is independent of any effect on the peripheral vasculature (MacCarthy et al., 2000). In the right ventricle of human hearts from PAH patients, there was a significant increase in the ratio of ET_A_ to ET_B_ receptors compared with normal hearts as well as a significant increase in the medial layer of small pulmonary arteries. The latter provide the rationale for efficacy of ET antagonists, although the increase in ET_A_ in the ventricle is likely to be an adaptive response to left heart failure to cause a beneficial increase in cardiac output. This would be blocked in the setting of ET antagonism (Kuc et al., 2014). Interestingly, the density of the ET_A_ subtype is increased by 50% in the left ventricle of patients with ischemic heart disease, whereas ET_B_ receptor density is unaltered compared with nonfailing hearts (Peter and Davenport, 1996b).

### D. Function of Endothelin Receptors in the Cardiovascular System

#### 1. Endothelin A Receptor Constriction in the Human Cardiovascular System.

The two receptor subtypes exhibit contrasting cardiovascular actions under normal physiologic conditions. ET-1 is the most abundant isoform in the human cardiovascular system. On release from the endothelium, primarily directed abluminally, the peptide causes vasoconstriction, mainly via ET_A_ receptors that are the principle subtype in the vascular medial layer (comprising mainly smooth muscle cells) in large conduit arteries and veins as well as small resistance vessels (Fig. 4). Electron microscope autoradiography has confirmed unequivocally the presence of high densities of ET_A_ but low densities of ET_B_ receptors in smooth muscle cells with few receptors of either subtype associated with the surrounding collagen (Russell et al., 1997).

In some, but not all, human vessels, ET_B_ mRNA and a low density of ET_B_ receptors (usually <15%) have been detected in the medial layer from certain vessels (Fig. 8, Davenport et al., 1993, 1995a,b). Extensive pharmacological characterization of human isolated vessels shows that ET_B_ agonists such as BQ3020 do not have any activity up to a concentration of 10 *μ*M. Occasionally some vessels were found to respond to the more potent sarafotoxin 6c, but the magnitude of the response was much less than for ET-1. ET-3 was at least two orders of magnitude less potent than ET-1 consistent with activating ET_A_ rather than ET_B_ receptors. Finally all antagonists tested to date, such as BQ123, cause a parallel and rightward shift of the ET-1 concentration response curve in all vessels examined with Schild slopes close to unity (Maguire and Davenport, 1995, Pierre and Davenport, 1998a,b). Although there is a consistent pattern of ET_A_-mediated constriction in human vessels, in animals the relative contribution is more complex, with vasoconstriction via ET_A,_ ET_B_, or a mixture of both depending on species or vascular beds. For example, BQ3020 and sarafotoxins 6c are potent constrictors of rat renal and mesenteric beds; BQ123 does not fully block these responses.

In agreement with in vitro studies in humans, infusion of BQ123 caused vasodilatation in volunteers when the ET_A_ antagonist was infused into the brachial artery (Haynes and Webb, 1994), a finding repeated in other clinical studies. These observations are consistent with blocking the action of ET-1 that is being continuously released from the endothelium on vascular smooth muscle ET_A_ receptors. This was significant as antagonists of other vasoconstrictors, such as ANGII, do not alter blood flow in normotensive individuals. In marked contrast, infusion of an ET_B_-selective antagonist BQ788 caused systemic vasoconstriction in healthy volunteers, showing that the main consequence of activation of endothelial ET_B_ receptors by tonically secreted ET-1 was the physiologic basal release of nitric oxide (Verhaar et al., 1998).

The pharmacological action of ET-1 is very unusual compared with other vasoactive mediators such as ANGII. It remains the most powerful constrictor of human vessels discovered with a remarkably long-lasting action. For example, after intracisternal injection of 10 pmol ET-1 into dogs, the diameter of the basilar artery was 76% of the control after 3 days (Asano et al., 1989). No human vessel has been reported that does not respond to ET-1, suggesting the peptide is the "universal" vasoconstrictor. ET-1 is thought to play a major physiologic role in regulating vascular function and the flow of blood in most, if not all, organ systems, including heart, kidney, lungs, and liver. Other cell types that synthesize the peptide can also modulate vascular reactivity, including perivascular neurons in the periphery, perivascular astrocytes in the CNS, and, under pathophysiological conditions such as atherosclerosis, macrophages and monocytes. Overproduction of ET in pathophysiological conditions from the endothelium and other cellular sources can lead to constriction of vessels in conditions such as PAH, leading to vascular remodeling. This is exacerbated particularly where there is reduced production of opposing vasodilators such as nitric oxide, prostacyclin, and endothelium-derived hyperpolarizing factor. Activation of the ET_A_ receptor can drive proliferation in multiple cell types and contributes to fibrosis and inflammation.

It has often been reported that ET-1 binds irreversibly to ET_A_ receptors. This raises a critical question about the tractability of ET_A_ receptors as therapeutic targets in, for example, cerebral vasospasm, where antagonists if they are to be effective are required to reverse an established constrictor ET response (Pierre and Davenport, 1999). However, although dissociation of [^125^I]-ET-1 from cloned ET_A_ receptors was slow compared with other ligands with a half-life of the about 6 hours (as would be expected for long-lasting constrictor responses), binding was not irreversible (Blandin et al., 2000). Similarly, about 20% of specific [^125^I]-ET-1 binding had dissociated from native ET_A_ receptors in human aorta after 20 minutes (Maguire et al., 1996). Established ET-1 responses can be reversed in vitro and in vivo by receptor antagonists (Warner et al., 1994; Pierre and Davenport, 1999).

#### 2. Does Endothelin B Receptor-Mediated Constriction Change in Human Vascular Disease?

Detailed pharmacological studies have investigated whether there is functional evidence for an upregulation of ET_B_ receptors in human isolated coronary arteries. Constrictor responses to sarafotoxin 6c were variable, with 40% of healthy arteries and 50% of vessels containing an atherosclerotic lesion not responding to the ET_B_. Where vessels did respond, there was no difference in the maximum responses obtained in healthy and diseased tissues. There was also no change in ET_B_ receptor density in the medial layer of atherosclerotic compared with control arteries (Maguire and Davenport, 2000). These results were supported by clinical studies. BQ123 caused coronary vasodilation and improved endothelial dysfunction in patients with atherosclerosis or its risk factors (Halcox et al., 2001). In a further 39 patients, ET_B_ antagonism caused coronary microvascular constriction and not dilatation, without affecting epicardial coronary tone or endothelial function. Combined ET_A_ and ET_B_ blockade, however, dilated coronary conduit and resistance vessels and improved endothelial dysfunction of the epicardial coronary arteries (Halcox et al., 2007). Thus the results of both in vitro and in vivo studies show that ET_A_ receptors contribute to basal constrictor tone with no evidence for constrictor ET_B_ receptors in human atherosclerosis. Animal studies where ET_B_ receptors are increased in pathophysiological conditions, in for example rat coronary arteries, are not representative of humans (Skovsted et al., 2014).

Of particular relevance is what happens in PAH, the current target of ET antagonists. McCulloch et al. (1996) found ET-1 caused a biphasic response in pulmonary resistance arteries (150–200 *μ*m internal diameter) with constrictor responses to low concentrations of ET-1 (<1 nM) blocked by an ET_B_ antagonist, but at higher concentrations, greater than 1 nM, ET-1 responses were blocked by an ET_A_ but not an ET_B_ antagonist, suggesting that at levels of ET-1 in the pathophysiological range ET_A_ receptors will be activated. Davie et al. (2002) quantified the ratio of the two receptor subtypes in small arteries (500–1000 *μ*m) from PAH patients and healthy controls. Although overall ET receptor density in distal arteries was twofold greater than in controls, there was no change in any vessel in the ratio of ET_A_ to ET_B_. These results suggest that ET_B_-mediated constrictor responses occur at low ET concentrations, but in the absence of a change in subtype ratio it is unlikely there would be increased ET_B_ constrictor response in this patient group.

#### 3. Endothelin B Receptor Vasodilatation.

Although the vascular endothelium represents only 1% of the weight of the vessel wall it is distributed as a monolayer throughout all vessels in the body. The paradox that the potent vasoconstrictor ET-1 was also a vasodilator was quickly recognized after its discovery and was particularly evident from early in vivo studies (Wright and Fozard, 1988), because vasodilator responses can be lost during the preparation of vessel for in vitro research. ET-1, released from endothelial cells, functions in an autocrine manner to feed back onto endothelial ET_B_ receptors to release vasodilators, principally nitric oxide, but also prostacyclin or endothelium derive hyperpolarizing factor depending on the vascular bed. This feedback mechanism limits the constrictor actions of ET via smooth muscle ET_A_ receptors and has an essential physiologic role in cardiovascular homeostasis. ET-1 is continuously released from the endothelial constitutive pathway. Low levels of ET-1 promote vasodilatation, whereas higher and pathophysiological concentrations increase blood pressure and total peripheral vascular resistance. In ET-1+/− heterozygous mice, levels of ET-1 in plasma and lung tissue were lower than wild type, and the animals developed *elevated* blood pressure and mild hypertension rather than the fall in blood pressure that might have been expected (Kurihara et al., 1994) but explainable by ET_B_-mediated dilatation as the predominant action of ET-1 in the wild-type mice.

In healthy volunteers, low doses of ET-1 infused into the brachial artery cause vasodilatation consistent with ET_B_-mediated release of vasodilators, but this was followed by sustained vasoconstriction of the forearm vascular bed at higher doses because the peptide accessed the smooth muscle ET_A_ receptors (Kiowski et al., 1991). High concentrations of ET-3 also cause vasodilatation (Haynes et al., 1995a,b). Blocking ET_B_ receptors using BQ788 caused vasoconstriction in healthy volunteers, showing that the main consequence of activation of endothelial ET_B_ receptors by tonically secreted ET-1 was the physiologic basal release of nitric oxide (Verhaar et al., 1998). Similarly, ET_A_ receptor antagonism caused vasodilatation in the peripheral circulation of volunteers and patients with chronic heart failure, whereas selective ET_B_ receptor antagonism caused vasoconstriction in each group. ET_B_ receptor antagonism may therefore cause potentially deleterious vasoconstriction in chronic heart failure (Love et al., 2000). Only nitric oxide, from a range of potential vasodilators tested as physiologic antagonists, was found to fully reverse ET-1-mediated constrictions in human epicardial coronary arteries (Wiley and Davenport, 2000, 2001a) and also inhibited ET-1 binding (Wiley and Davenport, 2001b). It is well established that nitric oxide inhibits ET-1 synthesis, for example in cultured endothelial cells (Ohkita et al., 2002) and synthetic ECE-1 enzyme (Kuruppu et al., 2014).

Thus ET_B_-mediated release of nitric oxide and other vasodilators is crucial in acting as a counterregulatory pathway to limit ET_A_-mediated vasoconstriction through stimulation of vascular cyclic GMP. In pathophysiological conditions where there is endothelial cell dysfunction resulting in a loss of vasodilators, the vasoconstrictor and other pathophysiological effects of ET-1, such as cell proliferation, will be potentiated. In addition, Big ET-1 levels are raised in some condition such as heart failure (Love et al., 2000), and alternative pathways for ET-1 synthesis from Big ET-1, for example by the vascular smooth muscle, would result in ET-1 binding immediately to ET_A_ receptors and thus negating activation of the endothelial ET_B_ feedback pathway that opposes vasoconstriction.

#### 4. Endothelin B Receptor Clearing Receptors.

In addition to vasodilatation, endothelial ET_B_ receptors function as clearing or scavenger receptors to internalize the ligand-receptor complex and remove ET-1 from the circulation (Fig. 4; Gasic et al., 1992; Fukuroda et al., 1994b). Systemic and selective blockade of ET_B_ receptors results in a significant rise in circulating ET-1 (Plumpton et al., 1996). Importantly this is not simply the result of displacement of ET-1 release from the endothelium that would otherwise have bound to vascular ET_A_ receptors, because plasma ET-3 levels also rise. Increases in plasma ET-1 have also been reported for mixed antagonists that block both receptors, such as bosentan, where there is a dose-dependent rise in plasma ET-1 (see, for example, Williamson et al., 2000). With mixed antagonists, provided ET_A_ receptors are blocked it is unlikely that ET-1 will have any adverse effect, whereas selective blockade of ET_B_ may potentially be detrimental. In contrast, administration of the selective ET_A_ antagonists in animals and humans usually does not increase but in some cases reduce plasma ET-1 levels compared with nonselective antagonists, which have been documented to increase ET-1 levels. For example, in patients with heart failure acute ET_A_ antagonism by sitaxentan caused selective pulmonary vasodilatation associated with a reduction in plasma ET-1 (Givertz et al., 2000). Monitoring changes in plasma ET-1 provides an elegant biomarker of receptor occupancy of ET_B_ receptors and also a guide as to the selectivity of antagonists in vivo.

Clearance of ET-1 in humans is very efficient and was reported to be biphasic. The half-life of the initial phase was 4 minutes, with a longer late phase that had not reached basal after 1 hour (Vierhapper et al., 1990). A biphasic curve was obtained with Big ET-1 with a half-life of 7 minutes and 23 minutes for the late phase (Hemsen et al., 1995). By comparison, ET-3 plasma half-life was 1.7 minutes (Weitzberg et al., 1995). In humans, the lungs (Dupuis et al., 1996), liver (Gandhi et al., 1996), and kidney (Saito et al., 1991) are important sites for removal of ET-1. This reflects that these organs are ET_B_ rich (Fig. 7): Seventy percent of the ET receptors in both the cortex and medulla of the kidney are ET_B_ and localize to endothelial cells throughout the renal vasculature consistent with clearing ET-1 (Kuc et al., 1995; Gariepy et al., 2000). In lung comprising about 50% ET_B_, the majority are present on the vascular endothelium. The isolated perfused liver extracts proportionately more ET-1 than the lungs, with 80% uptake in a single pass through binding to ET_B_ receptors on hepatic stellate cells and is reduced in conditions such as cirrhosis (Rockey and Weisiger, 1996). Plasma levels of ET-1 are increased in patients with cirrhosis, and elevated concentrations in the liver are thought to be a consequence of both increased synthesis and decreased clearance (Hinterhuber et al., 2004).

The technique of positron emission tomography (PET) using [^18^F]-ET-1 allows the dynamic distribution and uptake to be visualized in all organs (Johnstrom et al., 2005). This study confirmed the rapid removal from the circulation within 2 minutes, and the major sites for clearance of circulating [^18^F]-ET-1 were the lungs, the liver, and the kidney (particularly the glomeruli, cortex, and inner medulla/papilla), with only low levels of uptake visualized in the thyroid, pituitarym and salivary glands and no binding to the skeleton or brain, the latter indicating the peptide did not cross the blood-brain barrier. BQ788 injected *before* [^18^F]-ET-1 significantly reduced clearing in lung and kidney by 85% confirming the role of the ET_B_ subtype, although interestingly [^18^F]-ET-1 significantly increased in the liver because the label was no longer cleared by ET_B_ receptors and now bound to the ET_A_ subtype. BQ788 injected after [^18^F]-ET-1 did not displace the radioligand, consistent with internalization of ET-1 by ET_B_ receptors and degradation in the lysosome (Bremnes et al., 2000). [^18^F]-ET-1 could not be visualized to the heart despite expressing this organ expressing ET_B_ receptors, but binding was detected under BQ788 blockade. These results emphasize the importance of ET_B_ receptors in maintaining low circulating plasma levels and the potential for ET-1 levels to rise under pathophysiological conditions where clearance is reduced. ET_B_ receptors are expressed by a number of cell types in addition to endothelial cells including epithelial and smooth muscle cells. Do these contribute to removal of ET-1? Selective deletion of endothelial ET_B_ receptors in mice (with other ET_B_ receptors expressed by other cell types unaffected with no change in ET_A_) resulted in impaired clearance of an intravenous bolus of labeled ET-1 (Bagnall et al., 2006; Kelland et al., 2010b). [^18^F]-Big ET-1 was also rapidly cleared from the circulation of the rat (half-life ∼3 minutes) with uptake of radioactivity in three major organs: the liver and lungs were major sites for conversion (inhibited by phosphoramidon), whereas in the kidney [^18^F]-Big ET-1 was excreted largely unmetabolized (Johnstrom et al., 2010).

### E. Receptor Heterodimers?

GPCRs were originally considered as monomeric proteins, but it is now well established that GPCRs are able to form dimers, particularly receptors in Family C, and there is some evidence for a number of Class A receptor dimers, e.g., *β*1 and *β*2 adrenoceptors, CCR2 and CCR5 (Pin et al., 2007). NC-IUPHAR has published a number of criteria that should be met before a heterodimer can be established. There should be a specific pharmacological property, such as a ligand-specific response of the heterodimer. There should be evidence for physical association in native tissues or primary cells; coimmunolocalization using antibodies recognizing each of the subunits should be used, ideally by electron microscopy level, energy transfer technologies, and recombinant fluorescent proteins. Knockout animals or RNAi technology may also provide key information on the existence of heterodimeric GPCRs in vivo.

Gregan et al. (2004) demonstrated that ET_A_/ET_B_ heterodimers artificially expressed in HEK293 cells could be coimmunoprecipitated using ET_B_-specific antibodies, detected using FLAG-tagged ET_A_ and myc-tagged ET_B_ receptors and confirmed using fluorescence resonance energy transfer microscopy. The authors observed a pharmacological difference in that ET_A_/ET_B_ heterodimers internalized more slowly in response to ET-1 than with BQ3020. If this occurred in native tissues this could change the number of functional ET_B_ receptors on the cell surface and also the clearance of ET-1. Evans and Walker (2008a,b) explored evidence for both homo (ET_A_/ET_A_, ET_B_/ET_B_)- and heterodimers (ET_A_/ET_B_). These studies used energy transfer technologies and coimmunoprecipitation to show close association for each of the dimer pairs and a pharmacological difference between homodimers that when stimulated by ET-1 mediated a transient increase in calcium but a sustained increase was obtained to ET-1 with ET_A_/ET_B_ heterodimers. These studies meet some of the required criteria, but were all done in HEK293 cells. Further experiments are required in native tissues to determine whether heterodimers exist, for example, in epithelial cells where both subtypes are expressed and whether such dimers have any physiologic or pathophysiological significance.

## IV. Classification of Selective Agonists and Antagonists

Over the last 20 years the development of agonists and antagonists of varying degrees of specificity for both receptors has been extensive, not only as pharmacological probes but also for candidate drugs. The most concise overview (many compounds from which are discussed individually in this section) is maintained on the IUPHAR/BPS Guide to PHARMACOLOGY (GotPdb) web site (Pawson et al., 2014; Southan et al., 2016). The entry for ET_A_ antagonists is at guidetopharmacology.org/GRAC/ObjectDisplayForward?objectId=219 and for ET_B_ at guidetopharmacology.org/GRAC/ObjectDisplayForward?objectId=220, where further information on structure-activity and molecular structures can be downloaded. Although the GtoPdb entries are an expert-curated core set, the much larger number of receptor-to-compound mappings in ChEMBL represents a maximal compilation extracted from the medicinal chemistry literature (PMID 24214965). Release 20 of ChEMBL (https://www.ebi.ac.uk/chembl/) specifies 3885 unique chemical structures mapped to ET receptors, but this includes inactive compounds and entries are not categorized pharmacologically as agonist or antagonist. As a triage, these 3885 records were filtered to distinct structures with a reported affinity greater than 1 *µ*M then mapped to PubChem Compound Identifiers. This resulted in 1133 chemical structures with binding data for the ET_A_ receptor, 534 for the ET_B_, 456 against both, 677 A-only, and 78 B-only. Lists for these sets of ChEMBL IDs are available as supplementary data on figshare (http://dx.doi.org/10.6084/m9.figshare.1549677).

**Fig. 9.**
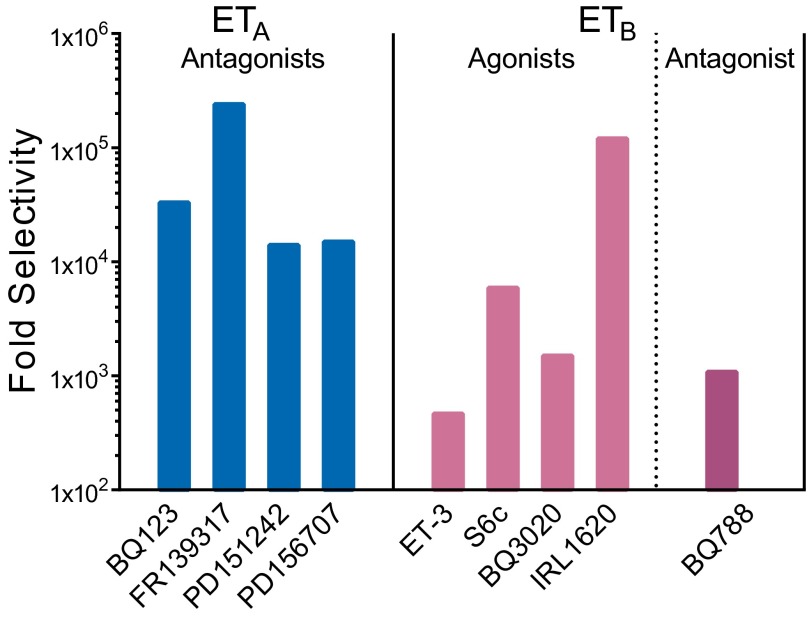
Comparison of the selectivity of ET ligands for native ET_A_ and ET_B_ receptors measured in the same competition binding assay against [^125^I]-ET-1. This assay uses human heart, which expresses both subtypes, resulting in a biphasic competition curve. Measurement of the affinity (the equilibrium dissociation constant or *K*_D_) of each compound at the two receptor sub-types can be accurately determined using non-linear iterative curve fitting. Comparison of the two affinities provides a measure of the selectivity for each subtype (Maguire and Davenport, 2012).

A comparatively large number of nonpeptide antagonists were developed for potential clinical use. ET receptor antagonists are classified as either selective for one receptor subtype or alternatively as mixed or "balanced" antagonists that block both receptors. The classification is usually made by the company or group discovering the compound by measuring the affinity at the two subtypes and calculating a ratio of selectivity, but there is no agreed definition. We have proposed ET_A_-selective compounds should display more than 100-fold selectivity for the ET_A_ subtype and those with less than 100-fold ET_A_ selectivity should be classified as mixed antagonists (Davenport and Maguire, 2006). Similarly, ET_B_ agonists and antagonists should display 100-fold selectivity to be useful research compounds. In practice, the tool compounds described below display at least two orders of magnitude selectivity and, provided appropriate concentrations are chosen, would be expected to be selective in both in vivo and in vitro assays. In contrast, a number of antagonists developed for clinical use display marginal selectivity (Fig. 9). Bosentan is commercially available and is a mixed ET_A_/ET_B_ antagonist. One of the first orally active nonpeptide antagonists to be discovered, it has been widely used in in vitro and experimental models.

### A. Agonists

ET-1 is equipotent for ET_A_ and ET_B_ and will therefore activate both. ET-1 has been widely used in pharmacological preparations in vitro and in vivo as well as experimental medicine studies in volunteers and patients. Most studies characterizing and localizing ET receptors have used [^125^I]-ET-1 (directly labeled via Tyr^13^, a residue not critical for ligand-receptor interaction) that binds with the same affinity to both subtypes. This radiolabeled analog of ET-1 is stable under nonphysiologic binding conditions with little or no degradation being detected. ET-1 can also be labeled with the positron emitting isotope [^18^F] and used to image and quantify ET receptors in vivo (Johnstrom et al., 2005). [^125^I]-ET-2, [^125^I]-VIC, and [^125^I]-sarafotoxin 6b have also been labeled and used in saturation assays where they also bind to both subtypes (Davenport and Morton, 1991; Bacon and Davenport, 1996; Maguire et al., 1996). Binding was also detected using [^125^I]-ET-Big ET-1 labeled at Tyr^13^ to follow processing by enzymic cleavage to the mature labeled peptide, whereas no specific binding was detected with Big [^125^I]-ET-1 labeled at Tyr^31^, where enzymic activity yielded the inactive C-terminal fragment (Russell et al., 1998). [^18^F]-ET-Big has also been used to quantify and characterize conversion in vivo using PET (Johnstrom et al., 2010).

Some authors preferred to inject Big ET-1 instead of ET-1 for in vivo studies in the rat designed to characterize antagonists such as bosentan, arguing that the conversion to the mature peptide by the vasculature is a better representation of the ET signaling pathway (Clozel et al., 1994). However, in a detailed study that compared the ability of bosentan to block the actions in vivo of ET-1 versus Big ET-1, it was clear that the responses were not the same, with the authors concluding that ET-1 triggered a covert mesenteric vasodilator mechanism that was antagonized by bosentan but not seen with the precursor (Gardiner et al., 1994).

#### 1. Endothelin Receptor A-Selective Agonists.

No ET_A_-selective agonists, either peptide or nonpeptide, have been discovered.

#### 2. Endothelin Receptor B-Selective Agonists: Sarafotoxin 6c, IRL1620, BQ3020, and Radioligands.

Initially receptor subtypes were characterized by their rank order of affinity for the three ET peptides: ET_A_: ET-1 = ET=2 > ET-3 versus ET_B_: ET-1 = ET-2 = ET-3. Therefore, ET-3 is the only endogenous ET peptide that distinguishes the two receptor subtypes. For ligand binding assays, ET-3 can be radiolabeled at three positions: Tyr^6^, Tyr^13^, and Tyr^14^. Tyr^6^ is generally used because it is more difficult to separate [^125^I]-ET-3 labeled at the latter two Tyr residues, although all three ET-3 ligands have similar affinities. In practice, the selectivity of ET-3 for ET_B_ versus ET_A_ receptors is often only about two orders of magnitude (Fig. 9), and it is difficult to precisely delineate the two subtypes in functional experiments without the use of selective antagonists. ET-3 has been used in clinical studies, for example by Haynes et al., (1995a, b).

Four synthetic peptides have become established as the most highly cited compounds that are selective agonists at the ET_B_ receptor with greater selectivity than ET-3, and interpretation of results for these are unequivocal. Sarafotoxin 6c, one of the four sarafotoxin isoforms originally identified from the snake venom, has remarkably high selectivity for rat ET_B_ receptors (200,000-fold', Williams et al., 1991), although less so for ET_B_ human receptors (Fig. 9; Russell and Davenport, 1996). The peptide is resistant to peptidases. [Ala^1,3,11,15^]-ET-1 (Saeki et al., 1991) is a linear analog of ET-1 where substitution of Ala for Cys residues removes the disulfide bridges, converting the ET-1 structure to an ET_B_ agonist. The most widely used ET_B_-selective agonists are IRL1620 (Suc-[Glu^9^, Ala^11,15^]-endothelin-1_8-21_) (Takai et al., 1992), which is a truncated linear analog where the N terminus has an *N*-succinyl modification that reduces metabolism by nonspecific peptidases, and BQ3020 ([Ala^11,15^]Ac-ET-l_6-21_) (Ihara et al., 1992b). Sarafotoxin 6c has been used in experimental medicine studies in humans (Haynes et al., 1995a, b). All four peptides have been radiolabeled to characterize ET_B_ receptors, although [^125^I]-BQ3020 and [^125^I]-IRL1620 have been most frequently used (Ihara et al., 1992b; Molenaar et al., 1992). [^125^I]-BQ3020 binds with subnanomolar affinity to the ET_B_ receptor with 1500-fold selectivity for this subtype over the ET_A_ receptor, and [^18^F]-BQ3020 has been used as a selective PET imaging agent in vivo (Johnstrom et al., 2000, 2006). [^125^I]-IRL1620 is also highly ET_B_ selective (Watakabe et al., 1992).

#### 3. Endothelin Receptor B Agonists in Chemotherapy and Neuroprotection.

ET_B_ agonists have been tested for clinical application in cancer (Maguire and Davenport 2012; Gulati et al., 2012). A Phase 1 clinical trial of IRL-1620 (SPI-1620, Spectrum Pharmaceuticals; (Henderson, Nevada)) was carried out in patients with recurrent progressive carcinoma and shown to selectively and transiently increase tumor blood flow (Gulati et al., 2012). The rationale for the study was that stimulating ET_B_ receptors would cause vasodilatation to increase the penetration of cytotoxic antitumor agents into tumors but reduce the concentration in healthy tissue. This hypothesis was based on the reports that IRL1620 increased effectiveness of antitumor agents in rat models of prostrate and breast cancer (Rajeshkumar et al., 2005) as well as improving the efficacy of radiotherapy (Gulati et al., 2012). Preclinical studies have suggested IRL1620 may also be effective in animal models of stroke and cerebral ischemia (Leonard et al., 2011, 2012; Leonard and Gulati, 2013). Interestingly, the complementary approach of ET_A_ antagonism has also been suggested as potential benefit in these models (Patel et al., 1996).

### B. Antagonists

#### 1. Endothelin Receptor A Peptide and Nonpeptide Antagonists and Radioligands.

The most widely cited ET_A_-selective peptide antagonists used in vivo and in vitro are BQ123 (cyclo-[d-Asp-l-Pro-d-Val-l-Leu-d-Trp-], (Ihara et al., 1992b), originally isolated from fermentation products of the microorganism *Streptomyces misakiensis*, that has low nanomolar affinity for the receptor, followed by the linear peptide FR139317 (*N*- [(hexahydro-1-azepinyl)carbonyl]l-Leu(1-Me)d-Trp-3 (2-pyridyl)-d-Ala; Aramori et al., 1993) (Figs. 3 and 4). These antagonists are commercially available and are recommended for their very high selectivity for ET_A_, their solubility, and lack of plasma binding (Fig. 9). Owing to their peptidic structure they do not have oral bioavailability, are administered intra-arterially, and are metabolized or excreted over comparatively short periods of time. BQ123 and FR139317 are therefore used for short-term, acute investigations in both animal models and in experimental medicine studies. BQ123 in particular has been used extensively in acute studies in volunteers and patients with no adverse effects reported at the concentrations tested. A second peptide antagonist, the cyclic hexapeptide TAK-044 (Masuda et al., 1996), also isolated from *Streptomyces misakiensis* with a more modest degree of ET_A_ selectivity, has also been used in experimental (Plumpton et al., 1996; Haynes et al., 1996; Ferro et al., 1997) and clinical studies including renal failure (Dhaun et al., 2007a) and in an a multicenter, randomized, double-blind, placebo-controlled trial in patients with subarachnoid hemorrhage (Shaw et al., 2000).

Key tool compounds for radioligand binding and autoradiography (using a fixed concentration of ligand) include [^125^I]-PD151242 that is recommended to localize ET_A_ receptors. This linear tetrapeptide, based on the initial FR139317 structure, binds with subnanomolar affinity to the ET_A_ receptor and has about 10,000 fold selectivity for this subtype in human (Davenport et al., 1994a,b) and animal tissues (Peter and Davenport, 1995a,b). A tritiated analog, [^3^H]-BQ123, has also been reported to be a highly specific and reversible ET_A_ radioligand (Ihara et al., 1995), although, as with other tritiated ligands, it has much lower specific activity than[^125^I]-labeled compounds.

#### 2. Endothelin Receptor A-Selective Antagonists in the Clinic: Ambrisentan and Withdrawal of Sitaxentan.

**Fig. 10.**
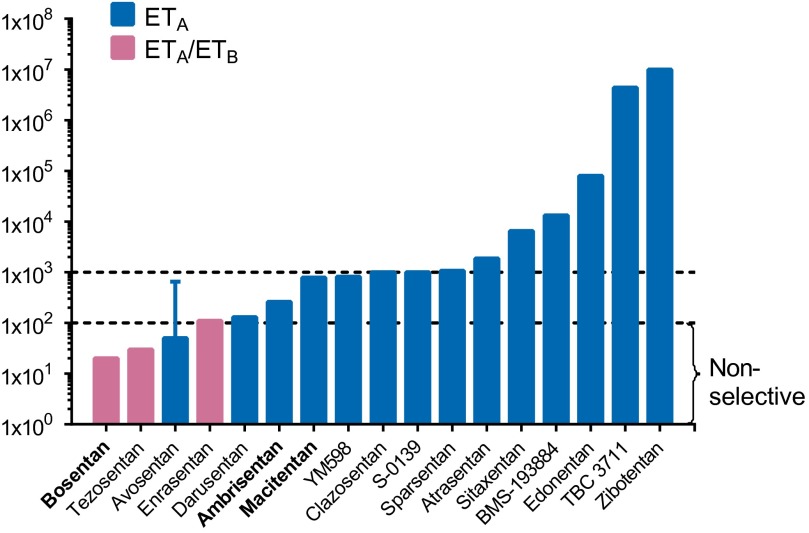
Spectrum of selectivity of antagonists for ET_A_ versus ET_B_ receptors as reported by the companies that discovered the compounds mainly based on measuring affinity constants in separate competition assays against [^125^I]-ET-1 using human recombinant ET_A_ versus ET_B_ receptors. Antagonists are classified as either selective for one subtype or alternatively as mixed antagonists that block both receptors. The classification is usually made by the manufacturer, but there is no agreed definition and there are anomalies. We proposed that antagonists that are ET_A_-selective should display more than 100-fold selectivity for the ET_A_ subtype and those that block both ET_A_ and ET_B_ (mixed antagonists) should demonstrate less than 100-fold ET_A_ selectivity. The rational for this classification is based on receptor occupancy where a concentration can be calculated to block virtually all ET_A_ receptors but not ET_B_ for in vitro studies. The threshold is indicated by the lower dashed line. It is difficult to achieve exact plasma concentration in vivo and to achieve selectivity; compounds with at least two orders of magnitude are more useful. Bosentan, ambrisentan, and macitentan are currently approved for clinical use and are highlighted. Note that the manufacturers of macitentan classify the antagonist as a mixed antagonist although it displays ET_A_ selectivity. Sparsentan is a dual ET_A_/angiotensin II receptor type 1 receptor antagonist (modified from Maguire and Davenport, 2014).

The majority of experimental studies have used a number of commercially available experimental ET_A_ antagonists (Palmer, 2009). These include PD156707 (Maguire et al., 1997) that has also been used to image ET_A_ receptor in vivo using PET (Johnstrom et al., 2000; Maschauer et al., 2013) and A127722 (ABT627, atrasentan) (Wessale et al., 2002). A nonpeptide ET_A_ selective ligand, an analog of PD156707, has been developed, [^125^I]-PD164333 (Davenport et al., 1998), as well as [^18^F]-PD156707 (Johnstrom et al., 2000). Preclinical studies characterizing the pharmacology of ET_A_ small molecule antagonists as well as mixed antagonists are shown in Fig. 10.

Ambrisentan (Letairis, Volibris, LU208075, BSF 208075) (Vatter et al., 2003) was approved in 2007 by the Food and Drug Administration and European Medicines Authority in 2008 as an orally active, once daily drug for the treatment of PAH (Table 1). The compound is a competitive ET_A_ antagonist, although less ET_A_ selective than sitaxentan, but has good bioavailability, long half-life with a low incidence of acute hepatic toxicity, and does not induce or inhibit P450 enzymes, so less likely to affect the pharmacokinetics of P450-metabolized drugs. Ambrisentan has fewer side effects, such as an increase in liver enzymes that require liver function tests that is associated with bosentan, but nasal congestion and peripheral edema are more prevalent (Vizza et al., 2012).

**TABLE 1:** Pharmacodynamic and pharmacokinetic properties of ET receptor antagonists approved for clinical use

	Bosentan	Macitentan	Active Metabolite of Macitentan	Ambrisentan	Sitaxentan (Withdrawn from Clinical Use in 2010)
Trade name	Tracleer,	Opsumit		Letairis, Volibris	Thelin
Chemical Name	benzenesulfonamide	sulfamide	sulfamide	benzenepropanoic acid	3-thiophenesulfonamide
Inhibition [^125^I]-ET-1 IC_50_ (nM, *Ki)					
ET_A_	5*	0.5	3	0.3	1
ET_B_	97*	391	185	185	9800
ET plasma levels after administration	↑↑	↑↑	—	↑	↓
Bioavailability	∼50%	Not reported	Not reported	High	70–100%
Time to max plasma concentration	3–5	4-12	30	1.7–3.3	1–4
Terminal half-life (h)	5.4	16	40.2-65.6	15	10
Excretion in urine (%)	<3	50%	—	Low	50–60

(Weber et al., 1996;^1999^; Spence et al., 2008; Opitz et al., 2008; Sidharta et al., 2013)

In 2015, clinical trials with ambrisentan (clinicaltrials.gov) included the treatment of portopulmonary hypertension, hypoplastic left heart syndrome, inoperable chronic thromboembolic pulmonary hypertension, PAH associated with systemic sclerosis, and exercise-induced PAH. Analysis of clinicaltrials.gov showed that there were no records for the majority of investigational ET antagonists with the exception of two compounds. Zibotentan, the most ET_A_ selective of the small molecule compounds (Fig. 10) was discontinued from development by Astra-Zeneca (Cambridge, United Kingdom) after it failed to show any improvement in survival in Phase III trials in patients with resistant prostate cancer metastatic to bone (Nelson et al., 2012). The antagonist was made available to other groups and trials included patients with intermittent claudication and renal scleroderma. Atrasentan (AB 627) is the most extensively studied ET_A_ antagonist in preclinical studies and has shown promise in patients with type 2 diabetic nephropathy, reducing albuminuria, blood pressure, cholesterol, and triglyceride levels (de Zeeuw et al., 2014), and this has led to the initiation of a Phase 3 multicenter trial (Sonar) with 4000 patients (clinicaltrials.gov).

Sitaxentan (Table 1; Thelin, TBC11251) (Wu et al., 1997; Barst et al., 2007) was introduced in 2008 as a potent, competitive, long-acting, orally active ET_A_ antagonist, the most highly selective to be approved for the treatment of PAH. The compound was withdrawn is 2010 owing to cases of idiosyncratic hepatitis resulting in acute liver failure (Don et al., 2012).

#### 3. Endothelin B Receptor Peptide and Nonpeptide Antagonists.

Few ET_B_ receptor antagonists have been developed, reflecting the potential danger of blocking the beneficial vasodilatation and internalization of ET mediated by this subtype (Palmer, 2009). The most extensively used and highly cited is the peptide BQ788 (Ishikawa et al., 1994), although a small number of papers have reported on other compounds such as the first small molecule, nonpeptide RO468443 that displayed 2000-fold ET_B_ selectivity (Breu et al., 1996) and A192621. The latter compound has the advantage over BQ788 because it has good oral bioavailability in a number of species (Wessale et al., 2002). ET_B_ antagonists are generally less potent than ET_A_ antagonists and display lower selectivity (usually only 1 to 2 orders of magnitude) for the ET_B_ over the ET_A_ receptor, but concentrations can be chosen that selectively block the ET_B_ receptor. Other proposed antagonists should be avoided, and results are difficult to interpret for compounds such as RES-701-1 that had little or no action, for example, at human or rat ET_B_ receptors (Tanaka et al., 1995; Russell and Davenport, 1996) or have low affinity, such as IRL-2500 (Russell and Davenport, 1996). Radiolabeled ET_B_ antagonists have not been developed.

### C. Mixed Antagonists in the Clinic: Bosentan and Macitentan

Bosentan is a sulfonamide (Palmer, 2009) and was the first ET orally active antagonist to be approved for clinical use in the treatment of PAH (Table 1). It has a comparatively short half-life but good bioavailability, and as a competitive mixed antagonist, it will block both the detrimental effects of ET-mediated via ET_A_ as well as beneficial actions mediated via ET_B_. Bosentan is the most widely used mixed antagonist in preclinical studies and has been radiolabeled with tritium (Dashwood et al., 1995). The mixed antagonist SB209670 has been labeled with ^18^F (Johnstrom et al., 2004). In common with other dual antagonists, bosentan tends to have lower rates of fluid retention and edema than observed with the ET_A_ antagonists. However, liver toxicity is a significant side effect; this is not thought to be as a consequence of blocking ET receptors but by inhibiting the bile salt export pump leading to accumulation of cytotoxic bile salts, resulting in hepatocellular damage. In 2015, bosentan clinical trials included scleroderma renal crisis, ischemic optic neuropathy, stable severe chronic obstructive pulmonary disease, and combined with iloprost in PAH (clinicaltrials.gov).

Macitentan (Table 1, Opsumit, ACT-064992; Actelion) is a sulfamide that was approved for clinical use in PAH in 2013 (Patel and McKeage, 2014; Sidharta et al., 2015). It was designed using structure-activity studies to improve the efficacy and tolerability of bosentan. Macitentan has been proposed to enter the liver via passive diffusion and not by active uptake as is the case of bosentan (Treiber et al., 2014), and, as a result of reduced liver toxicity, doses do not need to be altered in patients with renal or hepatic disease (Dingemanse et al., 2014). Macitentan has fewer interactions with other drugs and reduced edema/fluid retention compared with bosentan (Bruderer et al., 2012; Gatfield et al., 2012). The pharmacokinetics are more complex: it is an insurmountable antagonist that is metabolized to ACT-132577, which is also active albeit with a lower potency, but the metabolite reaches a higher plasma concentration and has a longer half-life than the parent compound macitentan (Iglarz et al., 2008; Sidharta et al., 2013, 2014). Macitentan is an order of magnitude more potent than bosentan, as a result of longer receptor occupancy (17 minutes versus 70 seconds for bosentan measured by in vitro assays), probably as a result of interaction with different amino-acid residues in the ET receptors. Actelion describes the compound as a dual antagonist but on the basis of their own data measuring inhibition of [^125^I]-ET-1 binding to human expressed receptors it displays ∼800 fold selectivity for the ET_A_ subtype (Pulido et al., 2013). However, plasma ET-1 concentrations were significantly increased (twofold at the highest dose tested), suggesting blocking of ET_B_ occurs at the dose used (Sidharta et al., 2011). In 2015, clinical trials with macitentan included thromboembolic pulmonary hypertension, portopulmonary hypertension, and Eisenmenger Syndrome. Perhaps the most intriguing new target is glioblastoma, where the ET pathway is upregulated (Harland et al., 1998) in combination with existing therapy, temozolomide (clinicaltrials.gov).

### D. Will Endothelin Agonists and Antagonists Be Developed in the Future?

**Fig. 11.**
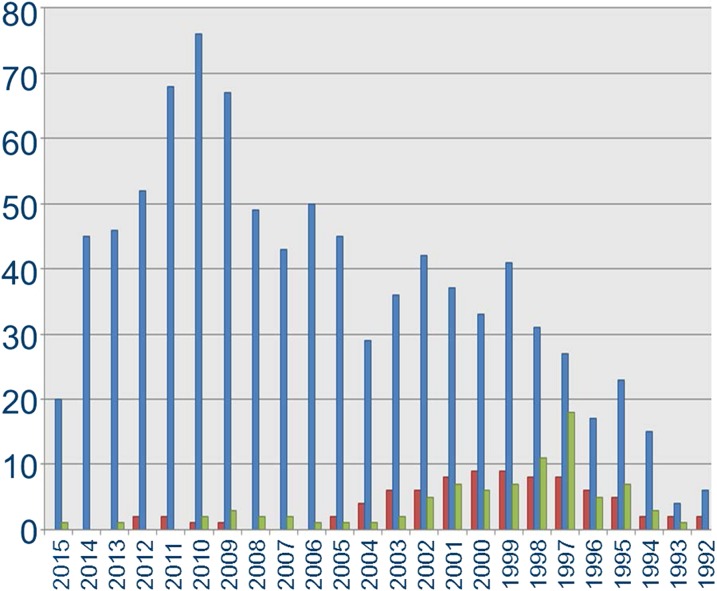
Pipeline of ET receptor antagonists measured by papers in PubMed (blue bars), papers with ChEMBL entries in Europe PubMed Central (red bars) and patents (green bars) in PATENTSCOPE with ET receptor antagonists combined with patent classification C07D (code for medicinal chemistry) (Bento et al. 2014).

The first peptide receptor antagonists were discovered in 1992 (section IV.B.1). ET receptors continue to be a challenging drug target. First, the key question as to whether selective ET_A_ receptor antagonists are clinically advantageous over dual ET_A_/ET_B_ antagonism is still open. Second, in a review based on commercial intelligence sources, up to 2009, only three antagonists were approved for the clinic, bosentan, sitaxentan, and ambrisentan from ∼500 preclinical compounds (Palmer, 2009). These challenges are reflected in a primary patent literature review in the same year (Mucke, 2009). The latter concluded that pharmaceutical companies have reduced ET antagonist primary patenting (first filings on novel structure activity series) since ∼2000 because most potential therapeutic applications have already been claimed. Figure 11 shows that although publications on ET antagonists reached a peak in 2011, patenting and medicinal chemistry output was already declining by the late 1990s and only a small number of patents continue to be filed, most recently in 2015. This latter is a series of phenoxybutanoic acid derivatives with a lead compound identified as a potent and selective ET_A_ antagonist with efficacy in animal models of hypoxia-induced PAH (Cai et al., 2015).

**Fig. 12.**
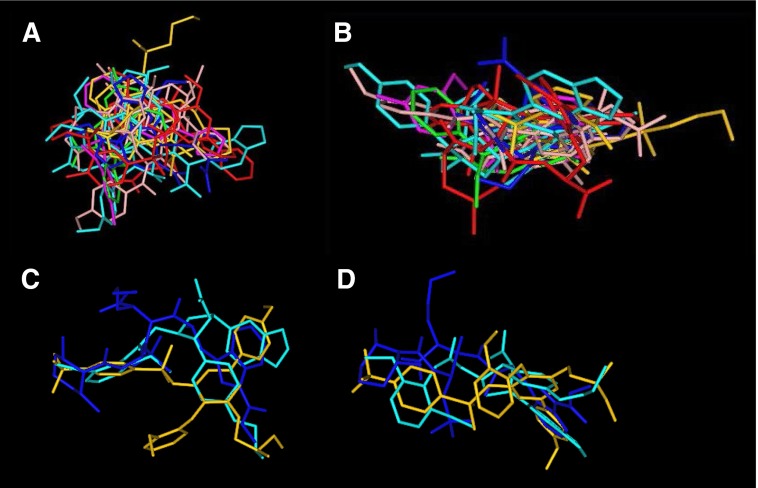
Three-dimensional alignments shown as a top view (A) and the side view (B) of the 11-molecule cluster of ET antagonists using ChemAxon Marvin tool at default settings to perform a flexible overlap of atoms via on-the-fly adjustments. The following 11 were selected as having high affinity: peptide antagonist FR139317 and nonpeptide antagonists, A127722, ambrisentan, avosentan, darusentan, macitentan, PD156707, PD164333, SB234551, sitaxentan, and zibotentan. Top (C) and side view (D) of a three-molecule cluster of ET_B_ peptide antagonist, BQ788, and nonpeptide antagonists A192621 and RO468443.

The results show that to date ET agents are chemically very similar and there is further chemical space to be exploited. Both Palmer (2009) and Mucke (2009) commented that although the explored and claimed chemical space is large (the space spanned by all potential chemical compounds), the ET antagonists that have progressed are mostly N-heterocyclic sulfonamides of comparable structure and molecular weight and those that are not sulfonamides (atrasentan, ambrisentan, darusentan, and enrasentan) still retain strong similarities with each other and with the sulfonamides. One way to examine this characteristic for a set of molecules is via a three-dimensional alignment using the ChemAxon Marvin tool. Figure 12 shows antagonists with a range of ET_A_ selectivity. The overlays imply that this compact conformational ensemble circumscribes an antagonist binding pocket (note that the extended orange side chain is from macitentan). Although such results should not be over interpreted, the side view suggests that this pocket is flattened in terms of what conformations can access it to exert the biologic effects. The analogous results are presented for the ET_B_-selective antagonists A-192621, BQ788, and RO468443 in Fig. 12.

### E. Allosteric Modulators

Allosteric modulators bind to a separate region of a given receptor to the orthosteric agonist binding site that binds the endogenous ligand, in this case ET-1. Negative allosteric modulators would be expected to reduce ET-1-mediated responses. Currently, ET receptor antagonists carry a black box warning because of the risk of the embryo-fetal toxicity inferred from ET-1 KO studies in mice. Allosteric modulation, in reducing but not blocking ET-1, may have an advantage in lessening this possibility. Only a limited number of allosteric modulators of the ET_A_ receptor (Blandin et al., 2000; Talbodec et al., 2000; Farhat and Allen et al., 2004; Ahmed et al., 2008) have been reported and none for the ET_B_ subtype. These would all be classified as negative modulators of ET-1 binding such as sodium salicylate (Talbodec et al., 2000). This compound and related molecules normally act as nonsteroidal anti-inflammatory drugs by inhibiting cyclo-oxygenase activity. It is therefore difficult to interpret the results of testing the action of such compounds in vivo on the cardiovascular actions of ET-1, because they would also inhibit the production of vasodilators such as prostacyclin. Acetyl salicylate has not been reported to interact with any other G-protein coupled receptors. However, from the limited set of reports it is not possible to draw any structure-based conclusions on allosteric modulators. Based on the small molecular size [for example, molecular weight of 296 for 3,5-dibromosalicylic acid and low potency, *K*_i_ ∼300 *µ*M (Blandin et al., 2000)], the usefulness of structures reported so far for SAR expansion seems limited. Although the absence of additional results should not be over interpreted, given the extent of chemical space, it would seem a reasonable assumption that there may not be easily exploitable allosteric effector pockets in these receptors.

### F. Therapeutic Monoclonal Antibodies: Rendomab-B1

A new strategy to block the action of ET-1 is the development of a monoclonal antibody, rendomab-B1, which has subnanomolar affinity at the ET_B_ receptor and was reported to be more effective than BQ788 in competing for ET-1 binding. Rendomab-B1 was proposed to function as an antagonist based on the observed inhibition of ET-1-induced IP_3_-calcium signaling (Allard et al., 2013). The antibody was not tested in vivo, and it might be expected, based on experience from BQ788 experimental medicine studies, to block endothelial ET_B_ receptors and therefore cause vasoconstriction. The rationale for clinical application is unclear compared with that for a monoclonal antibody against ET_A_ receptors. However, one advantage could be in linking the monoclonal to a toxic payload for cancer treatment of those tumors in which ET_B_ receptors are overexpressed. The generation of monoclonal antibodies against GPCRs is challenging. However the study provides proof of principle for the generation of a pharmacologically active monoclonal against the ET_B_ receptor. Membrane bound GPCRs are highly favorable targets for the development of monoclonal antibodies and these can have advantages over small molecule antagonist with respect to specificity and prolonged time course of action of several months.

### G. Targeting the Endothelin B Receptor with Cell Penetrating Peptides

The cell penetrating peptides (CPP) superfamily comprises several classes of peptides that deliver an exogenous moiety to a specific intracellular target. They include the protein-derived CPPs and pepducins that are lipidated peptides that remain tethered to the cell membrane but can interact with an intracellular segment of a GPCR resulting in activation or blockade of downstream signaling. Currently there are pepducin agonists and antagonists for many GPCRs including chemokine receptors, the *β*2 adrenoceptor, proteinase activated receptors, sphingosine-1-phosphate receptors, and the apelin receptor (for reviews, see Dimond et al., 2011; O’Callaghan et al., 2012; Zhang et al., 2015).

A CPP has been reported for the ET_B_ receptor incorporating an amino acid sequence based on the second intracellular loop of ET_B_ linked to a SynB3 vector derived from the antimicrobial protein protegrin-1 (Green et al., 2013). This molecule, IC2B, reduced right ventricular systolic pressure and hypertrophy and the degree of pulmonary artery muscularization in a rat model of hypoxia induced PAH without an effect on blood pressure. In acute studies, 24 hours of hypoxia increased levels of Akt and ERK in pulmonary artery smooth muscle, and these were attenuated by injection of IC2B at the beginning of the hypoxic period. In isolated pulmonary artery from the same animals, in the presence of the ET_A_ antagonist BQ123, ET-1 further activated Akt and ERK phosphorylation and this was also blocked by ICB2 confirming involvement of the ET_B_ receptor (Green et al., 2013). These data are somewhat unexpected because blockade of ET_B_ receptors has generally been shown to be detrimental in PAH. The authors suggested that the beneficial effect may result from a selective blockade of smooth muscle ET_B_ receptor-mediated vasoconstriction and proliferation via Akt and ERK signaling that contribute to disease progression, leaving endothelial ET_B_-mediated vasodilatation for example unaffected; however, this remains to be confirmed.

### H. Biased Signaling

A major development in GPCR research has been the discovery of "biased ligands" that stabilize the receptor in a conformation that preferentially activates a subpopulation of the G-protein-dependent and/or -independent cascades that are normally engaged by an unbiased natural ligand (for reviews, see Kenakin, 2011; Wisler et al., 2014). Previously, the assumption had been that ET-1 acting at the ET_A_ receptor, for example, would elicit G-protein-dependent effects such as vasoconstriction and stimulate *β*-arrestin recruitment to silence the receptor by desensitization and subsequent internalization. ET receptors are internalized via *β*-arrestin and dynamin/clathrin-dependent mechanisms, but the receptor subtypes exhibit clear differences; ET_A_ receptors are recycled to the plasma membrane and are therefore available for further signaling, whereas, after internalization, the ET_B_ subtype is directed to the lysosomes and degraded (Bremneset al., 2000). However, it is now apparent that *β*-arrestins can also contribute to physiologic and, importantly, pathophysiological responses such as cell migration and proliferation (for review, see Shukla et al., 2011). Pathway biased ligands are therefore of great interest, and the development of biased drugs have the potential to limit detrimental on-target effects. To date, agonists have been identified that show either G-protein bias, for example at the nicotinic acid (Walters et al., 2009), *μ* opioid (deWire et al., 2013), and apelin receptors (Brame et al., 2015), or *β*-arrestin bias, such as the angiotensin II receptor type 1 (Violin et al., 2010) and parathyroid hormone receptors (van der Lee et al., 2013).

There have been some limited studies that indicate that the ET peptides and sarafotoxins show some pathway bias at the ET receptors, at least in vitro, in some assays. Compeer et al. (2013) proposed that ET-1 and ET-2 activate different signaling mechanisms to elicit and maintain vasoconstriction that were dependent on the vascular bed investigated. It is now recognized that signaling bias is best demonstrated by comparing the relative potencies of agonists to a standard, preferably an endogenous agonist, in assays that interrogate multiple G-protein-dependent and -independent pathways that have relevance to ET physiology or pathophysiology. One study (Maguire and Davenport, 2012) has reported that the endogenous ET peptides show similar rank order of potencies and maximum responses in both a G-protein-dependent constrictor assay and cell-based *β*-arrestin recruitment assay, whereas sarafotoxin 6b was a full agonist in the constrictor assay but a partial agonist in the *β*-arrestin assay, suggesting the potential to generate biased ET agonists. It is interesting to speculate that a reduction in the ability of the toxin to trigger receptor desensitization may have been a result of evolutionary selection to prolong the effect on prey of envenomation. Of perhaps more relevance to the development of improved clinical compounds, it was shown that although bosentan does not distinguish between ET_A_ and ET_B_ receptors in radioligand binding assays, this compound exhibited a higher affinity in the ET_A_-mediated *β*-arrestin recruitment assay compared with the ET_B_-mediated *β*-arrestin assay.

The potential to develop *β*-arrestin biased antagonists is intriguing because detrimental effects of ET-1, particularly in cancer, may result from activation of *β*-arrestin-mediated signaling. The contribution of the ET-1/ET_A_/*β*-arrestin pathway is well defined in epithelial ovarian cancer and contributes to tumor cell proliferation, invasion, and metastasis. ET-1 induced activation of nuclear factor kappa-light-chain-enhancer of activated B cells, a key mediator in oncogenesis, is dependent on *β*-arrestin (Cianfrocca et al., 2014), and *β*-arrestin-1 is an epigenetic regulator of ET-1-induced *β*-catenin signaling, a critical mechanism for controlling cell division and ovarian cancer progression (Rosanò et al., 2009, 2013b). Selective activation of the ET_A_/G*α*s pathway was recently proposed as an alternative strategy in this disease (Teoh et al., 2014) because this G-protein is linked to tumor-suppressive effects. In this study it was reported that ET_A_/G*α*s stimulated cAMP and subsequent activation of protein kinase A inhibited the expression of angiogenic and metastasis genes that are switched on by ET_A_-mediated *β*-arrestin signaling.

### I. Pharmacological Targeting of Epigenetic Regulation of Endothelin Signaling

Epigenetic mechanisms are crucial for normal development and maintenance of tissue-specific gene expression. These mechanisms include DNA methylation and histone modification, the latter being posttranslational covalent modification of globular histone proteins by a range of writers, erasers, and readers that alters the ability of associated DNA to be transcribed. These covalent marks are read by specific families of proteins such as bromodomains that can now be targeted by small molecule inhibitors, which selectively modulate gene expression (Prinjha et al., 2012).

Epigenetic regulation is critical to the ET pathway because transcription is the primary level of regulation of the endothelin-1 gene by histone modifications and DNA methylation (Welch et al., 2013). This regulation can be affected in disease by alterations such as aberrant DNA methylation in the promoter regions of the gene. For example, in the cardiovascular system, ANG II induces cardiac hypertrophy and fibrosis partly by stimulating ET-1 transcription. This occurs as a result of an epigenetic complex of chromatin remodeling proteins and histone methyltransferase being recruited to the ET-1 promoter region in endothelial cells (Weng et al., 2015). Conversely downregulation of the ET pathway may be detrimental in cancer. In rat and human colon tumors, hypermethylation of *EDN2* and *EDN3* genes resulted in the epigenetic inactivation of ET-2 and ET-3 mRNA and corresponding protein. Restoring ET-2 and ET-3 expression in human cells significantly attenuated the migration and invasion of human colon cancer cells (Wang et al., 2013). During development of tumors, DNA methylation silences the *EDNRB* gene and downregulates the receptor protein, reducing beneficial ET_B_-mediated apoptosis. Hypermethylation of the *EDNRB* gene was highly prevalent in gastric cancer and remarkably related to infiltration and metastasis that leads to the tumor progression (Tao et al., 2012). Epigenetic drugs may have efficacy where changes in ET signaling are thought to cause some tumor types such as melanomas and oligodendrogliomas (Bagnato et al., 2011).

### J. Physiologic Antagonism by Nitric Oxide and Other Vasoactive Mediators

A key physiologic action of nitric oxide, the endothelium derived relaxing factor, is to activate soluble guanylate cyclase and generated cGMP to cause relaxation. Endothelial nitric oxide synthase is constitutively active, generates NO in response to shear stress and other physiologic stimuli and can limit the constrictor action of ET-1. This has recently been reviewed (Rapoport, 2014). A number of pathophysiological conditions are associated with endothelial cell dysfunction and loss of nitric oxide. The balance between ET and nitric oxide appears critical. In contrast to nitric oxide, shear stress downregulates the transcription of the ET-1 gene and may be related to cell shape and cytoskeletal change (Malek et al., 1993). It is well established that inhibitors of NOS increase levels of ET-1. In rats, in vivo treatment with NOS inhibitor to cause hypertension resulted in increased ET-1 synthesis in renal microvessels when NO production is suppressed. ET-1 was found to be a major activator of collagen I formation in renal resistance vessels and in the development of renal fibrosis (Tharaux et al., 1999). In humans in vivo, nitric oxide has been demonstrated to be a major component of ET_B_-mediated vasodilation (Verhaar et al., 1998, see section III.D.1 and 3). As noted (section III.D.3) in a study of human vessels in vitro that tested the potency of four endogenous directly acting vasodilators, nitric oxide atrial (ANP), brain, and C-type natriuretic peptides to oppose the actions of ET-1 (10 nM) in human vessels, only NO could fully reverse ET-1-mediated constrictions in conductance coronary arteries (Wiley and Davenport, 2000, 2001a, 2002). In human skin, ET-1 caused vasoconstriction at the injection site, but also vasodilatation in the surrounding area. The latter response is thought to be mediated by polimodal nociceptor fibers either releasing nitric oxide directly or are interconnected with nitrergic fibers innervating skin microvessels (Wenzel et al., 1998). Nitric oxide is also able to inhibit ET synthesis, and release from the vascular endothelium of porcine conduit vessels (Boulanger and Luscher, 1990) opposes the vasoconstrictor actions of ET-1 in small and resistant vessels of rabbits (Ito et al., 1991).

Other vasodilators also act as physiologic antagonists. For example, pressor responses to circulating and locally produced ET-1 were found to be terminated by a regionally selective effect of calcitonin gene-related peptide though cyclic nucleotide-independent relaxation of vascular smooth muscle (Meens et al., 2011; Labruijere et al., 2013). The apelin family of peptides potently reverses ET-1-induced vasoconstriction in isolated human vessels (Maguire et al., 2009). The apelin signaling pathway is downregulated in pulmonary arterial hypertension, suggesting it may be an important mechanism counteracting the pathophysiological actions of ET.

## V. Phenotypes of Global Genetic Modifications

Over the past 20 years, there has been an explosion of genetic manipulation tools that have been used to generate transgenic mice and rats. By using homologous recombination in embryonic stem cells (Evans and Kaufman, 1981; Smithies et al., 1985; Doetschman et al., 1987; Thomas and Capecchi, 1987), whole body knockout (KO) models have been developed. However, a limitation to this approach is that the target of interest is knocked out during all of development and may result in developmental disorders and a lethal phenotype, as is the case for ET-1, ECE-1, ET_A_, and ET_B_. To circumvent this obstacle, there are a number of genetic approaches including loxP/Cre (Sternberg and Hamilton, 1981; Lakso et al., 1992; Gu et al., 1993) or Flp/FRT (O'Gorman et al., 1991) that can be used to manipulate genomes in a cell-specific manner. These technologies have resulted in a large number of murine models. More recently, zinc finger nucleases have been used to edit genomes in a variety of animals, including rats (see Urnov et al., 2010), and dramatic progress has been made with the use of clustered regularly interspaced short palindromic repeats/Cas9 (Cong et al., 2013). There are now technologies that can be used to either knock down or knock in a target of interest, and these studies have been critical in furthering our understanding of the physiologic role of the ET axis. This review focuses on how global and cell-specific genetic modifications have been used to delineate the biologic function of components of the ET system.

### A. Endothelin-1/-2/-3

#### 1. Global Overexpression of Human Endothelin-1.

Whole body ET-1-overexpressing mice were generated by microinjecting linear human preproET-1 genomic DNA into single-cell embryos (Hocher et al., 1997). These hemizygous ET-1 transgenic mice (ET-1^+^) had normal circulating ET-1 concentrations but significantly higher ET-1 expression in the brain, lungs, and kidney. Furthermore, ET-1 concentration from total kidney extracts was significantly elevated in ET-1^+^ mice at both 3 months and 14 months of age (Hocher et al., 1997). As a consequence, ET-1^+^ mice presented with a significant increase in the number of renal cysts, inflammation, an increase in renal interstitial fibrosis, and glomerulosclerosis, resulting in a decline in renal blood flow and GFR that was independent of changes in mean arterial pressure (Hocher et al., 1997; Theuring et al., 1998; Shindo et al., 2002). ET-1^+^ mice also have significantly increased apoptosis of glomeruli, tubular, and interstitial cells, whereas proliferation was not different from control mice (Hocher et al., 1998). Furthermore, part of the increased renal inflammation and scarring was androgen dependent because castration of ET-1^+^ male mice reduced proteinuria, reduced CD4+ cell expression, and attenuated glomerulosclerosis and perivascular fibrosis (Kalk et al., 2009). Castration did not significantly affect GFR or renal CD8+ or macrophage expression, nor did it significantly affect renal interstitial fibrosis or media/lumen-ratio of renal arteries (Kalk et al., 2009).

Interestingly, ET-1^+^ mice have increased nitric oxide synthase (NOS)-2 expression in the renal vasculature and macrophages (Hocher et al., 2004) and increased urinary nitrite and nitrate (NOx) excretion while on a standard breeding diet, suggesting that the increased renal ET-1 may stimulate renal NO production (Quaschning et al., 2003). However, urinary NOx is greatly affected by dietary intake, and it is unclear if ET-1^+^ mice eat similar amounts of chow compared with control animals. Nevertheless, ET-1^+^ mice have a small but significantly greater aortic dilation in response to acetylcholine and have blunted constriction to exogenous ET-1 compared with control aortae, suggesting there is increased NO bioavailability that challenges the ET-1-induced aortic contraction (Quaschning et al., 2003). Moreover, intravenous injection of *N^ω^* -nitro-l-arginine methyl ester (l-NAME, 25 mg/kg) resulted in a significantly greater increase in blood pressure in ET-1^+^ mice compared with littermate controls (Hocher et al., 2004). These data suggest that activation of the NO axis limits ET-1-dependent increases in both acute and chronic blood pressure regulation in this model.

The interaction between ET-1 and NO is well documented throughout the literature. In the endothelium, ET-1 stimulates NOS3-dependent NO production, leading to vasodilation. In cardiovascular disease, there is often NO deficiency and elevated ET-1 that may be related. To determine the pathophysiological mechanisms of NO deficiency coupled with elevated ET-1, the ET-1^+^ mouse was crossed with the NOS3^−/−^ KO mouse (Vignon-Zellweger et al., 2011, 2014). The ET-1^+^NOS3^−/−^ mice and NOS3^−/−^ mice (both males and females) have a similar increase in systolic blood pressure and a similar reduction in heart rate compared with wild-type controls or ET-1^+^ mice (Vignon-Zellweger et al., 2011). These investigators used heart catheterization to observe that NOS3^−/−^ mice have an increase in left ventricular end-diastolic pressure, and female NOS3^−/−^ mice have reduced ventricular relaxation. However, ET-1^+^NOS3^−/−^ mice had relatively normal left ventricular end-diastolic pressure and heart relaxation. Female ET-1^+^NOS3^−/−^ mice develop perivascular fibrosis of intracardiac arteries more rapidly than males (Vignon-Zellweger et al., 2014). Female ET-1^+^NOS3^−/−^ and NOS3^−/−^ mice have reduced ventricular ET-1 mRNA compared with male ET-1^+^NOS3^−/−^ and NOS3^−/−^, which may partially explain the sex difference in the cardiac fibrosis observed (Vignon-Zellweger et al., 2014). Using a proteomics approach, Vignon-Zellweger at al. (2014) determined that ET-1^+^NOS3^−/−^ mice had increased cardiac antioxidant expression, reduced expression of proteins involved in destabilization of actin, and greater reduction in proteins that lead to cardiomyocyte hypertrophy compared with NOS3^−/−^ mice. Thus, increases in ET-1 in the context of NO deficiency may, under these unique circumstances, be beneficial for cardiac function, although clear sex differences exist in this context. A summary of mice overexpressing the endothelin system is listed in Table 2.

**TABLE 2:** Summary of the phenotype of mice with genetic overexpression of endothelin-1

Animal Model	Site (Cre)	Phenotype	References
Overexpressing			
ET-1^+^	Whole body	Lung: chronic inflammation; normal pulmonary pressure	Hocher et al., 1997, 2000;
		Kidney: increased renal cyst formation; renal interstitial fibrosis; glomerulosclerosis; age-dependent salt-sensitive hypertension	Shindo et al., 2002; Kalk et al., 2009
ET-1^+/+^/NOS2^−/−^	Whole body	increased blood pressure	Quaschning et al., 2008
ET-1^+^/NOS3^−/−^	Whole body	elevated blood pressure, reduced heart rate	Quaschning et al., 2007, Vignon-Zellweger et al., 2011, 2014
eET-1	Endothelium (Tie2-CRE)	Vascular endothelial dysfunction; vascular remodeling; increased lipid metabolism gene expression; normal blood pressure	Amiri et al., 2004; Simeone et al., 2011
TET-1	Endothelium(Tie1-CRE)	Elevated blood pressure; endothelial dysfunction	Leung et al., 2004
Apoe^−/−^eET-1	Whole body ApoE knockout, endothelial ET-1 overexpression	High-fat diet-induced atherosclerosis, abdominal aortic aneurysms, increase oxidative stress; increase immune cell infiltration	Simeone et al., 2011; Li et al., 2013
Inducible cardiomyocyte ET1^+/+^	Cardiomyocytes (alpha-myosin heavy chain -Cre)	Pulmonary and hepatic congestion; heart inflammation and hypertrophy	Yang et al., 2004
GET-1	Astrocyte (glial fibrillary acidic protein-Cre)	Normal development; normotensive; larger infarct size in response to ischemia/reperfusion injury; alleviates inflammatory pain	Lo et al., 2005; Hung et al., 2012
ET_A_^ET-1/+^	Where ET_A_ is expressed, ET-1 will be expressed	Constitutive ET_A_ activation; mandibular arch instead of maxillary arch; perinatal death	Sato et al., 2008
ET-1 H/+	Whole body	350% increased expression of ET-1; decreased plasma volume, heavy stiff hearts	Hathaway et al., 2015

ApoE, apolipoprotein E.

#### 2. Overexpression of Endothelin-2.

To determine the effect of increases in ET-2 expression, a human ET-2 transgene was inserted into the Sprague-Dawley rat genome (Table 3). These heterozygous rats, termed TGR(hET-2)37, express the ET-2 transgene in the kidney, adrenal gland, gastrointestinal tract, spleen, lung, and brain (Hocher et al., 1996; Liefeldt et al., 1999). Male hemizygous rats tended to have higher levels of circulating ET peptide (ET-1 and ET-2 are indistinguishable by available immunoassays) compared with female TGR(hET-2)37 (Liefeldt et al., 1999). ET-2 overexpression resulted in a significantly lower body weight, and heart-to-body weight and kidney-to-body weight ratios in males only. Basal blood pressure was similar between TGR(hET-2)37 and control rats and was also similar between males and females. Infusion of the NO synthase inhibitor, l-NAME, resulted in a significantly greater increase in blood pressure in male TGR(hET-2)37 rats compared with controls suggesting that overexpression of ET-2 results in greater NOS activity (Liefeldt et al., 1999). Male, but not female, TGR(hET-2)37 also had reduced creatinine clearance compared with controls. Finally, male TGR(hET-2)37 presented with significant interstitial and glomerular sclerosis (Liefeldt et al., 1999). Female hemizygous TGR(hET-2)37 had increased ET-1 mRNA in the glomeruli, and blood pressure-independent glomerulosclerosis and proteinuria compared with control rats (Hocher et al., 1996). These studies suggest that an increase in ET-2 can lead to organ sclerosis and that this is independent of blood pressure. In the context of diabetic cardiomyopathy, inducing diabetes (streptozotocin) in male TGR(hET-2)37 rats resulted in severe coronary and intramyocardial vessel hypertrophy and myocardial interstitial fibrosis compared with diabetic control rats (Liefeldt et al., 2010). Thus, elevated ET-2 levels can exacerbate organ damage in modeled disease states. Whether this is pathologically relevant to disease has yet to be established.

**TABLE 3:** Summary of phenotype of rats with genetic alterations of the endothelin system

Animal Model	Site (Cre)	Phenotype	References
TGR(hET-2)37	Whole body ET-2 overexpression	males: lower body weight, interstitial and glomerularsclerosis	Liefeldt et al., 1995, 1999
		females: glomerularsclerosis	Liefeldt et al., 1999
Homozygous spotting lethal (*sl/sl*)	Whole body, natural mutation in the ET_B_	aganglionic megacolon; white coat color; death at an early age.	Gariepy et al., 1996
D*β*H-ET_B_:ET_B_^sl/sl^	ET_B_ deficient except for nerves (insertion with dopamine-*β*-hydroxylase promoter)	ET_B_ expressed in nerves but absent elsewhere; coat color spotting; salt-sensitive hypertension	Gariepy et al., 1998; Gariepy et al., 2000; Pollock et al., 2000

#### 3. Endothelin-1, -2, -3 Knockout Mice.

##### a. Endothelin-1 knockout mice.

By disrupting exon 2 of the ET-1 gene, a whole body ET-1 KO mouse was developed (Kurihara et al., 1994). Mice homozygous for this deletion (ET-1^−/−^) were neonatal lethal. ET-1^−/−^ mice delivered by caesarian at day 18.5 postcoitum all displayed significant craniofacial and cardiac abnormalities, highlighting the critical developmental role of ET-1 (Kurihara et al., 1994, 1995b). Moreover, ET-1^−/−^ mice have reduced neonatal weight, impaired thyroid and thymus development (Kurihara et al., 1995a), and reduced cardiac sympathetic innervation (Ieda et al., 2004). Mice heterozygous for ET-1 deletion, ET-1^+/−^, appeared normal, were fertile, and had reduced lung and plasma ET-1 concentration; however they presented with an elevated systolic, diastolic, and mean arterial blood pressure (Kurihara et al., 1994). Interestingly, ET-2, ET-3, ET_A_, and ET_B_ expression were similar in a number of organs among neonatal wild-type, ET-1^−/−^ and ET-1^+/−^ mice, suggesting that other components of the ET system are not regulated by ET-1 in neonatal mice (Maemura et al., 1995).

##### b. Endothelin-2 knockout mice.

ET-2 was targeted by deletion of exon 1 and exon 2 (which encodes the mature peptide) (Chang et al., 2013). Although ET-2^−/−^ mice were born at the expected Mendelian frequencies and were observed nursing normally, at postnatal day 3 they were obviously smaller than ET-2^+/+^ pups and died by 3–4 weeks of age. ET-2^−/−^ mice had severe growth retardation, reduced blood glucose levels but normal insulin levels, and elevated blood urea nitrogen and uric acid, suggesting they suffered from internal starvation (Chang et al., 2013). Moreover, these mice presented with a reduced body temperature and abnormal lung histology. To determine the effect of ET-2 deletion in adult animals, Chang et al. (2013) also generated an inducible ET-2^−/−^ mouse (*ET2^f/f^:CAGGCre-ER*). Deletion of ET-2 was induced in both neonates and adults, and both presented with a mild hypothermia at ambient temperature and a significant drop in body temperature when challenged with cold for 12 hours (Chang et al., 2013). Thus, ET-2 is critical for metabolism and proper growth. Although ET-2 is highly expressed in the intestine and central nervous system, cell specific deletion of ET-2 resulted in a normal phenotype (see specifics in sections VI.B.8 and 9, respectively) and thus cannot explain the abnormalities observed in the global knockouts. What effect ET-2 deficiency has on other physiologic pathways, such as blood pressure control, remain to be determined.

##### c. Endothelin-3 knockout mice.

Whole body ET-3 KO mice were produced in a similar manner as the ET-1 KO mice: exon 2 of the ET-3 gene encodes for mature ET-3 and was targeted for deletion using homologous recombination in embryonic stem cells (Baynash et al., 1994). ET-3^+/−^ heterozygous mice were phenotypically normal and used to breed ET-3^−/−^ mice, which also were born with the expected Mendelian inheritance ratios. However, these mice only survived for an average of 21 days after birth (Baynash et al., 1994). ET-3^−/−^ mice have normal tidal volume and breathing frequency (even in hypoxic, hyperoxic, and hypercapnic environments) (Nakamura et al., 2001), thus pulmonary dysfunction seems unlikely to explain their early demise. Blood pressure measurements and baroreflex sensitivity appear to be normal in ET-3^−/−^ mice at ages 2–3 week compared with wild-type controls (Kuwaki et al., 2002), suggesting that at least while these mice are nursing, blood pressure is normal. However, what has not been tested is whether when challenged with different salt diets, if ET-3^−/−^ mice maintain normal blood pressure and baroreflex sensitivity. Because the ET_B_ receptor is the primary receptor for ET-3, we would expect the ET-3^−/−^ mice to have salt sensitivity similar to the ET_B_-deficient rats and CD ET_B_ KO mice (see sections V.B.4 and VI.B.12, respectively). However, the ET_B_ receptor does not exclusively bind ET-3, therefore ET-1 levels may compensate for the loss of ET-3.

ET-3^−/−^ mice presented with 70–80% of their coat being white (their background was C57blk/6J) but mainly their heads maintained a black coat. Histologic analysis of the skin determined the absence of melanin pigment and melanocytes (piebaldism) (Baynash et al., 1994), suggesting that ET-3 is critical for melanocyte melanin production. ET-3^−/−^ mice also present with aganglionic megacolon (which develops between 1 and 12 weeks of age), with an absence of the myenteric ganglia in the distal narrow segment of the colon but normal myenteric ganglion neurons between the smooth muscle of the proximal colon. From this study, it was determined that ET-3 is critical for normal development of vagal neural crest-derived enteric neurons and the trunk neural crest-derived epidermal melanocytes (Baynash et al., 1994). Moreover, this study identified ET-3 as the likely candidate gene that, if mutated, may lead to megacolon (Hirschsprung disease).

Piebaldism, commonly called lethal spotted (*ls*), arose spontaneously in a number of mice, including the C57bl/6J colony at the Jackson Laboratory. These mice also have megacolon. To confirm that it was ET-3 deficiency leading to this pathology, an ET-3 transgene was introduced into the *ls/ls* mouse under the control of the human dopamine-*β*-hydroxylase (D*β*H) promoter. During development of the enteric nervous system, vagal neural crest cells express D*β*H (Baetge and Gershon, 1989; Baetge et al., 1990a,b; Blaugrund et al., 1996). The *ls/ls* mice that harbor the D*β*H-ET-3 transgene had a reduction in piebaldism and were rescued from megacolon (Rice et al., 2000). Thus, ET-3 deficiency was identified as the mutated gene in the *ls/ls* mouse and provided evidence of a novel genetic target for Hirschsprung disease. A summary of all the endothelin system KO mice is presented in Table 4. Table 5.

**TABLE 4:** Summary of the phenotype of mice with knockout of the components of endothelin system

Animal Model	Site (Cre)	Phenotype	References
***ECE***			
ECE-1^−/−^	Whole body	Craniofacial and cardiovascular defects; aganglionic megacolon and coat color spotting; embryonic lethal	Yanagisawa et al., 1998a
ECE-2^−/−^	Whole body	No detectable abnormalities	Yanagisawa et al., 2000
ECE-1^−/−^ ECE-2^−/−^	Whole body	More severe cardiac abnormalities compared with ECE-1^−/−^ mice	Yanagisawa et al., 2000
***ET-1***			
ET-1^−/−^	Whole body	Abnormalities in craniofacial and cardiovascular system, thyroid and thymus gland; neonatal lethal	Kurihara et al., 1994, 1995a.b
ET-1^-/+^	Whole body	Mild blood pressure elevation; increased resting renal sympathetic nerve activity	Kurihara et al., 1994; Kuwaki et al., 1996; Ling et al., 1998; Morita et al., 1998; Kuwaki et al., 1999
ET-1 L/L or L/+	Whole body	20% and 65% ET-1 expression of a wild type mouse; dilated cardiomyopathy, elevated blood pressure, increased plasma volume	Hathaway et al., 2015
Neuron ET-1^−/−^	Neuron (synapsin I-Cre)	Greater sensitivity to acute nociceptive stimuli	Hasue et al., 2005
Cardiomyocyte ET-1^−/−^	Cardiomyocytes (alpha-myosin heavy chain -Cre)	Shorter life span; age-associated reduction in cardiac function	Zhao et al., 2006
Smooth muscle ET-1^−/−^	Smooth muscle protein 22-Cre	Normal right ventricular systolic pressure basally; hypoxia leads to an attenuated increase in right ventricular systolic pressure compared with control mice	Kim et al., 2015
VEETKO	Endothelium (Tie-2-Cre)	Hypotensive to normotensive; ANGII hypertension is similar to controls but a reduction in cardiac hypertrophy and fibrosis; attenuation of oxidative stress, inflammation and injury following renal ischemia/reperfusion injury, hypoxia or high salt feeding	Kisanuki et al., 2010; Adiarto et al., 2012; Arfian et al., 2012; Heiden et al., 2014; Heimlich et al., 2015
Collecting duct ET-1^−/−^	AQP2-Cre	Elevated blood pressure; salt-sensitive hypertension; impaired ability to excrete water	Ahn et al., 2004; Ge et al., 2005a, 2007, 2008b
***ET-2***			
ET-2^−/−^	Whole body	Growth retardation; internal starvation; severe hypothermia; lung dysfunction; death at an early age	Chang et al., 2013
inducible ET-2^−/−^ knockout	Whole body (CAGGCre-ER)	Reduced weight gain; reduced lipid deposition; mild hypothermia	Chang et al., 2013
Intestinal epithelium ET-2^−/−^	Villin-Cre	Normal growth and blood glucose level	Chang et al., 2013
Neuron ET-2^−/−^	Nestin-Cre	Normal core temperature; greater sensitivity to acute nociceptive stimuli	Chang et al., 2013
***ET-3***			
ET-3^−/−^	Whole body	Aganglionic megacolon; coat color spotting; normotensive and normal baroreflex sensitivity; death at an early age	Baynash et al., 1994; Kuwaki et al., 2002
ls/ls	Whole body, natural mutation in the ET-3 gene	Aganglionic megacolon; white spotting coat	Rice et al., 2000
ls/ls D*β*H-ET-3	Naturally ET-3 deficient but ET-3 overexpressed in nerves (insertion with dopamine-*β*-hydroxylase promoter)	Reduction in white spotting coat; rescued from megacolon	Rice et al., 2000
Piebald lethal s’/s’	Natural deletion of the ET_B_ gene from the whole body	Aganglionic megacolon; white spotting coat	Hosoda et al., 1994
***ET_A_***			
ET_A_^−/−^	Whole body	Craniofacial and cardiovascular defects: lethal minutes after birth	Clouthier et al., 1998
Cardiomyocyte ET_A_^−/−^	Cardiomyocytes (alpha-myosin heavy chain -Cre)	Normal development and cardiovascular function; ANGII or isoproterenol-induced myocardial hypertrophy: protected from cold stressed-induced cardiac fibrosis and dysfunction	Kedzierski et al., 2003; Zhang et al., 2012
Smooth muscle ET_A_^−/−^	Smooth muscle protein 22-Cre	Defects of arterial network, mandibular and thymus structure; death at an early age	Donato et al., 2014
Collecting duct ET_A_^−/−^	AQP2-Cre	Normotensive; impaired ability to excrete water	Ge et al., 2005b
Whole nephron ET_A_^−/−^	Inducible (Pax8-rtTA and LC-1)	Normotensive; fluid retention during high salt intake	Stuart et al., 2012; Stuart et al., 2013
***ET_B_***			
ET_B_^−/−^	Whole body	Aganglionic megacolon; white spotting coat; death at an early age	Hosoda et al., 1994
ET_B_^-/s’^	Whole body, 1 allele naturally mutated	Systemic ET_B_ deficiency; reduced incidence of megacolon; 50% white coat spotting; increased blood pressure; normal cardiac sympathetic and vagal activity; reduced brain and lung ET_A_	Ohuchi et al., 1999; Davenport and Kuc 2004; Kuc et al., 2006
ET_B_^−/−^+ET_B_-D*β*H	ET_B_ deficient except for nerves (insertion with dopamine-*β*-hydroxylase promoter)	ET_B_ expressed in nerves but absent elsewhere; white spotted coat; rescued from megacolon; elevated blood pressure; salt-sensitive blood pressure; endothelial dysfunction	Murakoshi et al., 2002; Quaschning et al., 2005
ECET_B_^−/−^	Endothelium (Tie-2-Cre)	Endothelial dysfunction; normotensive; red cell congestion of the intrahepatic branch of portal vein	Bagnall et al., 2006; Kelland et al., 2010b; Ling et al., 2012
Collecting duct ET_B_^−/−^	AQP2-Cre	Elevated blood pressure; increased ENaC activity; salt-sensitive hypertension	Ge et al., 2006; Bugaj et al., 2012
Collecting duct ET_A_^−/−^/ET_B_^−/−^	AQP2-Cre	Elevated blood pressure; increased ENaC activity; salt-sensitive hypertension	Ge et al., 2008a; Bugaj et al., 2012

**TABLE 5:** Clinical trials of ET antagonists

Compound	Class	Company	Conditions Studied
Ambrisentan	Selective ET_A_	Gilead Sciences	Pulmonary arterial hypertension *(licensed in Europe & USA)*
LU208075			Scleroderma
BSF208075			Pulmonary fibrosis
Atrasentan ABT-627/	Selective ET_A_	Abbott	Prostate cancer
A147627/			Diabetic nephropathy
A127722			Ischemic heart disease
Avosentan	Selective ET_A_	Speedel	Diabetic nephropathy
SPP301			
Bosentan RO470203	Mixed ET_A/B_	Actelion	Pulmonary arterial hypertension *(licensed in Europe & USA)*
			Scleroderma
			Hypertension
			Chronic heart failure
			Melanoma
Clazosentan	Selective ET_A_	Actelion	Subarachnoid hemorrhage
AXV034343			
VML588			
Ro611790			
Darusentan	Selective ET_A_	Gilead Sciences	Hypertension
LU125252			Chronic heart failure
BSF135252			
Edonentan	Selective ET_A_	Bristol-Myers Squibb	Chronic heart failure
BMS207940			
Enrasentan	Mixed ET_A/B_	GlaxoSmithKline	Chronic heart failure
SB217242			
Macitentan	Mixed ET_A/B_	Actelion	Pulmonary arterial hypertension *(licensed in Europe & USA)*
ACT-064992			Pulmonary fibrosis
Sitaxsentan	Selective ET_A_ Selective ET_A_	Encysive (acquired by Pfizer)	Pulmonary arterial hypertension Chronic heart failure
TBC11251		Retrophin	Chronic kidney disease
Sparsentan*			Hypertension
RE021			Focal & segmental glomerulosclerosis
Tezosentan	Mixed ET_A/B_ Selective ET_A_	Actelion AstraZeneca	Chronic heart failure
Ro 61-0612			Acute coronary syndrome
Zibotentan			Hepatorenal syndrome
ZD4054			Prostate cancer
			Ovarian cancer

* Dual ET_A_ and angiotensin receptor antagonist (Murugesan et al., 2005)

#### 4. High and Low Expression of Endothelin-1.

Recently, the Kakoki laboratory developed low-expressing (L) and high-expressing (H) ET-1 genes and used these to develop mice with four levels of ET-1 expression: L/L ∼20%; L/+ ∼65%; +/+ wild-type 100%; and H/+ ∼350% (Hathaway et al., 2015). Young adult L/L and L/+ mice have dilated cardiomyopathy, hypertension, and increased plasma volumes and reduced ventricular stiffness. The L allele can be spatiotemporally switched to the H allele by cre-loxP recombination. Global or cardiomyocyte-specific switching expression from L to H normalized the abnormalities in the L/L mice. H/+ mice have decreased plasma volumes and significantly heavy stiff hearts. These authors concluded that ET-1 is critical for maintaining normal contractile function and for ensuring that the myocardium has sufficient collagen to prevent overstretching because even a modest decrease in ET-1 expression is sufficient to cause cardiac dysfunction (Hathaway et al., 2015). The epithelial sodium channel (ENaC) blocker amiloride normalized plasma volume and blood pressure but only partially corrected the cardiomyopathy in the L/L mice. It is speculated that the reduced ET-1 levels mediated hypertension via increased ENaC activity, most likely in the renal collecting duct (see section VI.B.10). Interestingly, H/+ mice displayed reduced blood pressure suggesting that the higher level of ET-1 mediates greater inhibition of ENaC and lower plasma volume. Varying the expression of ET-1 with this genetic modification will allow investigators to further understand and tease apart the consequences of low and high ET-1 expression.

### B. Endothelin Converting Enzyme-1/Endothelin A Receptor/Endothelin B Receptor

#### 1. Global Knockout of Endothelin Converting Enzyme.

An ECE-1 KO mouse was developed targeting the exon upstream of the extracellular zinc-binding domain exon (Yanagisawa et al., 1998b). Unfortunately, ECE-1^−/−^ mice were embryonic lethal [and begin dying around embryonic day (E)12.5], although ECE-1^+/−^ mice were healthy and fertile (Yanagisawa et al., 1998b). E12.5 embryos of ECE-1^−/−^ mice presented with congestion and dilation of the atria and peripheral vessels, pericardial effusion, and generalized edema. A small percentage of ECE-1^−/−^ mice survived to term; however, due to severe craniofacial and cardiovascular abnormalities, these mice died within 30 minutes of birth (Yanagisawa et al., 1998b). As will be discussed later in reference to ET_B_ receptor gene deletion models (sections V.B.3 and V.B.4), it is important to note that these animals also did not develop enteric nerves. ECE-1 is critical for converting both Big ET-1 and Big ET-3 into their 21-amino acid active forms (Yanagisawa et al., 1998b). The ECE-1^−/−^ phenotype was more severe and resulted in earlier death than the ET-1^−/−^ or ET_A_^−/−^ mice, suggesting that active ET-1 and ET-3, by binding to both ET_A_ and ET_B_, are necessary for normal development. Moreover, given ECE-1 has broad substrate specificity (Johnson et al., 1999), it is possible that cleavage of nonendothelin peptides is also critical for embryonic development.

ECE-2 is also expressed during development: E10.5 embryos express *ECE-2* mRNA in the mesenchyme and neural tube and later in neurons and the developing heart (Yanagisawa et al., 2000). Knockout of ECE-2 from the whole animal, again targeting the exon that encodes the extracellular zinc-binding domain, resulted in no overt phenotype (Yanagisawa et al., 2000). ECE-2^−/−^ mice were healthy, with normal growth rates and survival, and were fertile. Histologic evaluation of a number of tissues did not reveal any abnormalities, which suggested that perhaps ECE-1 was compensating for the deletion of ECE-2. To test this hypothesis, the ECE-2^−/−^ mutation was introduced into the ECE-1^−/−^ mouse. This ECE-1^−/−^/ECE-2^−/−^ mouse had a very similar phenotype to the ECE-1^−/−^ mouse; however, they did present with broader and more severe cardiac abnormalities. Two interpretations are suggested from these data: (1) ECE-1 can compensate for a lack of ECE-2, resulting in normal heart development; and (2) ECE-2, which is expressed in the endocardial cushion of the developing heart, may lead to local ET-1 production that may limit cardiac abnormalities when ECE-1 is absent, although ECE-2 cannot fully compensate for the loss of ECE-1.

#### 2. Global Knockout of Endothelin A Receptor in Mice.

To delineate the physiologic and developmental role of the ET_A_ receptor, a whole body ET_A_ mouse was created by deleting exons 5 and 6 from the ET_A_ gene (Clouthier et al., 1998). ET_A_^−/−^ mice were viable up to parturition but presented with severe craniofacial deformities (similar to ET-1^−/−^ mice) and died about 30 minutes after birth (Clouthier et al., 1998). Specifically, ET_A_^−/−^ mice presented with a maxilla-like structure (upper jaw) in place of the lower jaw (Ruest et al., 2004). Interestingly, a specific deletion of ET_A_ from the developing mandible after neural crest cell patterning was generated (ET_A_^flox/flox^ mice bred with a Dlx5/6-Cre mouse to induce ET_A_ deletion in the mandibular first arch on E9.5) and resulted in normal jaw development, suggesting that ET_A_ is not critical for mandible bone development (Ruest et al., 2005). However, these studies firmly established that ET_A_ signaling is necessary for early neural crest cell patterning and proper lower jaw development.

ET_A_^−/−^ mice have a number of neural crest-derived structure abnormalities, including lack of the malleus, incus, and tympanic ring of the middle ear; a number of nerves emanating from the ganglia have abnormal distal ends; and abnormal cardiac and outflow tract development (Clouthier et al., 1998). Remarkably, ET_A_^−/−^ mice had normal peripheral nervous system development, although many of the nerves failed to project to the distal aspects of the mandibular arches. The authors concluded that the absence of ET_A_ does not significantly affect cranial ganglia patterning (Clouthier et al., 1998). The most common vessel abnormality in the ET_A_^−/−^ mouse was an interruption of the aortic arch between the left common carotid artery and the left subclavian artery (Yanagisawa et al., 1998a). The mechanism(s) of this deformity involves inappropriate regression of arch arteries 4 and 6, and this is either because of a lack of proliferation or an increase in apoptosis of the mesenchymal cells of these arches (Yanagisawa et al., 1998a). From these studies, it is clear that ET_A_ is critical for neural crest development.

Normally during development, there are epithelial-mesenchymal interactions in the first pharyngeal arch that guide cephalic neural crest cells to ultimately develop into the jaws. From the ET-1 and ET_A_ null mice, it was evident that ET-1/ET_A_ is necessary for lower jaw (mandibular arch) development. Further evidence came from the activated ET-1/ET_A_ signaling mouse, which was made by knock in of an ET-1 gene into one allele of the ET_A_ gene (Sato et al., 2008). These mice, ET_A_^ET-1/+^, have constitutive ET_A_ activation. Although ET_A_^ET-1/+^ died perinatally with severe craniofacial defects, examination of the early embryos revealed a dorsal-to-ventral transformation, resulting in a mandibular arch instead of the proper maxillary arch (Sato et al., 2008). In essence, these mice had duplicate lower jaw structures instead of a proper upper and lower jaw. This study demonstrates that ET-1-dependent ET_A_ activation specifies maxillo-mandibular identity. In summary, ECE-1, ET-1, and ET_A_ are all critical for neural crest patterning and proper jaw identity and development and cardiovascular development. Although these whole body KO models are lethal, they have greatly expanded our understanding of the role of the ET-1 system in development.

#### 3. Global Knockout of Endothelin B Receptor in Mice.

The ET_B_ receptor was also a candidate gene for causing Hirschsprung disease. To test this hypothesis, the ET_B_ gene was disrupted in exon 3 by insertion of a neomycin resistance cassette (Hosoda et al., 1994). ET_B_^+/−^ mice appeared phenotypically normal and were mated to produce ET_B_^−/−^ mice. ET_B_^−/−^ mice were born and developed greater than 90% of their fur coat as white despite being on a hooded mouse background. Although healthy for the first 2 weeks of life, they became increasingly sick and died within about 4 weeks (Hosoda et al., 1994). These mice presented with many of the same abnormalities as the ET-3^−/−^ mice, including severe distension of the intestine consistent with megacolon. Moreover, histologic analyses revealed an absence of neurons from the colon. These results led to the hypothesis that the ET_B_ gene may be deleted or mutated in another naturally occurring mutated mouse, the piebald-lethal *s′/s′*. To confirm this, Southern blots for ET_B_ receptors were performed in wild-type and *s′/s′’* mice and revealed that the ET_B_ receptor was mutated in the lung, heart, and intestine of the *s′/s′* mice (Hosoda et al., 1994). Thus, deletion or mutation of ET_B_ can also lead to Hirschsprung disease. From these studies with ET_B_^−/−^, ET-3^−/−^, and the naturally occurring mutated *s′/s′* mice, it is clear that ET-3/ET_B_ signaling is critical for development of neural crest-derived cell lineages, including melanocytes and the enteric nervous system.

Given that ET_B_^−/−^ mice have a premature death while *s′/s′* mice live longer, these mice were interbred to produce ET_B_^-/^*^s′^* mice to test hypotheses about the role of ET_B_ receptors in adulthood. Adult ET_B_^-/^*^s′^*mice have increased circulating ET-1 compared with ET_B_^+/+^ mice and a significant increase in mean, systolic, and diastolic pressure compared with ET_B_^+/+^ or ET_B_^+/^*^s′^* mice (Ohuchi et al., 1999). These mice also lacked a pressor response to an ET_B_ receptor antagonist, BQ788, that was observed in ET_B_^+/+^ and ET_B_^+/^*^s’^* mice. However, ET_B_^-/^*^s’^* mice have normal cardiac sympathetic and vagal activities (Ohuchi et al., 1999). One of the curious findings in ET_B_^−/−^ mice was a reduction of the brain ET_A_ receptor (Davenport and Kuc, 2004) and the lung ET_A_ receptor (Kuc et al., 2006). The mechanism(s) responsible for ET_A_ downregulation have not been determined, but could be related to the chronic elevations in ET-1. Furthermore, the mechanism of the hypertension in this model is unclear, although it may be due to a lack of renal ET_B_ receptors.

The lethal phenotype of the deleted ET_B_ receptor can be rescued in the ET_B_^−/−^ mice by driving ET_B_ transgene expression with the D*β*H promoter in the enteric nervous system (Murakoshi et al., 2002). These ET_B_-deficient mice (ET_B_^−/−^ + D*β*H-ET_B_^+/+^) presented with a white coat, but were rescued from megacolon. Blood pressure measured in the rescued mice at 14–16 weeks of age was significantly elevated and increased further when animals were placed on a high-salt diet (Murakoshi et al., 2002; Quaschning et al., 2005). Carotid artery ligation (as a model of vascular remodeling) resulted in a significantly larger intimal area, ratio of intimal to medial area, and stenotic ratio compared with wild-type mice (Murakoshi et al., 2002). This was coupled with a significant decrease in carotid nitrite/nitrate (NOx) concentration in the ET_B_-deficient mice. Furthermore, ET_B_-deficient mice have reduced acetylcholine-mediated aortic relation but normal sodium nitroprusside-dependent relaxation whether on normal or high-salt diet, suggesting they have endothelium-dependent dysfunction (Murakoshi et al., 2002). Finally, ET_B_-deficient mice have reduced ET_A_-mediated constriction, which is hypothesized to be due to a decrease in aortic ET_A_ receptor expression (Murakoshi et al., 2002). These studies suggest that deletion of the ET_B_ receptor or loss of ET_B_ receptor signaling accelerates vascular remodeling, reduces NO production, and leads to endothelial dysfunction.

However, in rat models of diabetes, the protective role of ET_B_ receptors may depend on the type of vascular being studied. Although ET_B_ antagonism increased unwanted remodeling in peripheral vessels there were a number of technical limitations in their study such as relatively small number of animals in the control group and ET_B_ receptors measured indirectly rather than by ligand binding.

#### 4. Endothelin B Receptor-deficient *sl/sl* Rats.

Similar to the piebald-lethal mice described above, a natural mutation called spotting lethal (*sl*) has been described in Wistar-Imamichi rats (Ikadai et al., 1979). Rats homozygous for this mutation present with whitening of their hooded coats and die within a few weeks of birth from megacolon. Work by Gariepy et al. (1996) determined that this spontaneous mutation occurs in the ET_B_ gene and that megacolon could be prevented by insertion of the D*β*H-ET_B_ transgene (Gariepy et al., 1998). Like in the mice described above, the D*β*H-ET_B_ transgene is expressed in the nervous system where it rescues the homozygous rats (D*β*H-ET_B_:ET_B_^sl/sl^) from megacolon, yet functional ET_B_ receptors are lacking in all other cell/tissues. As well, D*β*H-ET_B_:ET_B_^sl/sl^ rats present with significantly higher circulating ET-1 concentrations that increase even more when these rats are fed a high-salt diet (8% NaCl) (Gariepy et al., 2000). These data suggest that functional ET_B_ receptors play a role in regulating circulating ET-1 levels.

Both acute and chronic studies determining the physiology of the ET-1 system in the control of blood pressure have used the D*β*H-ET_B_:ET_B_^sl/sl^ model. Acute intravenous injections of 1 nmol/kg ET-1 result in an initial decrease in mean arterial pressure followed by a prolonged pressor response in transgenic control rats (D*β*H-ET_B_:ET_B_^+/+^). In contrast, a prolonged 30 mmHg increase in mean arterial pressure without the initial transient hypotension was observed in D*β*H-ET_B_:ET_B_^sl/sl^ rats (Gariepy et al., 2000). Furthermore, cumulative dose response curves to both ET-1 and the ET_B_ agonist sarafotoxin 6C (S6c) were generated to evaluate the pressor responses in D*β*H-ET_B_:ET_B_^sl/sl^ and heterozygous D*β*H-ET_B_:ET_B_^sl/+^ rats (Pollock et al., 2000). Doses of 0.1 nmol/kg, 0.3 nmol/kg, and 1 nmol/kg of either ET-1 or S6c resulted in a significant increase in mean arterial pressure in both genotypes. However, the increases in pressure were significantly greater in the D*β*H-ET_B_:ET_B_^sl/sl^ rats (Pollock et al., 2000). The significant sarafotoxin S6c-dependent increase in blood pressure in both heterozygous and D*β*H-ET_B_:ET_B_^sl/sl^ rats could be prevented by an ET_B_ antagonist but not an ET_A_ antagonist, suggesting that ET_B_-dependent pressor responses still exist in the ET_B_-deficient animals (Pollock et al., 2000), presumably a result of transgene expression in neuronal tissue.

The influence of dietary sodium on chronic blood pressure control has been extensively studied in the D*β*H-ET_B_:ET_B_^sl/sl^ rats. While on a sodium-deficient diet, mean arterial pressure was similar between transgenic control and D*β*H-ET_B_:ET_B_^sl/sl^ rats; a 3-week diet of 8% NaCl resulted in a significant (>40 mmHg) increase in mean arterial pressure in the D*β*H-ET_B_:ET_B_^sl/sl^ rats (Gariepy et al., 2000). Thus, lacking functional ET_B_ receptors in all tissues except the nervous system results in a severe, salt-sensitive hypertension. Intervention with amiloride, a diuretic that blocks ENaC, prevented the high salt-dependent increase in blood pressure in the D*β*H-ET_B_:ET_B_^sl/sl^ rats (Gariepy et al., 2000). These findings led to the hypothesis that the lack of functional ET_B_ receptors, likely in the collecting duct, results in hyperactive ENaC, sodium retention, and an increase in blood pressure. Interestingly, renal transplant studies between transgenic controls and D*β*H-ET_B_:ET_B_^sl/sl^ rats suggest that extrarenal ET_B_ receptors limit salt-sensitive hypertension, perhaps through regulation of renal blood flow, changes in sympathetic tone (Ohkita et al., 2005), or skin sodium storage.

Intrarenal ET-1 and functional ET_B_ receptors are very important for regulating natriuresis and diuresis. Using acute infusion of an ET_B_ agonist into the rat renal medulla, Nakano et al. (2008) determined that activation of medullary ET_B_ receptors leads to natriuresis and diuresis via a mechanism that is NOS1 dependent. Female and male D*β*H-ET_B_:ET_B_^sl/sl^ rats on a normal salt diet have higher blood pressure than genetic controls, although still within the normotensive range (mean arterial pressure is approximately 130 mmHg) (Speed et al., 2015). Nonetheless, there is significantly higher inner medullary NOS activity in female D*β*H-ET_B_:ET_B_^sl/sl^ rats (Taylor et al., 2003) that may provide an explanation for the more robust ability of female rats to excrete salt and water (Kittikulsuth et al., 2011, 2013). As well, interstitial intramedullary infusion of ET-1 resulted in a significant natriuresis in female D*β*H-ET_B_:ET_B_^sl/sl^ rats only, and this was dependent on NOS1 (Nakano and Pollock, 2009). However, 3 week of high-salt diet (10% NaCl), resulted in a similar increase in pressure in male and female D*β*H-ET_B_:ET_B_^sl/sl^ rats when measured by telemetry (Speed et al., 2015). High-salt diet resulted in a reduction in NOS activity in male and female control and D*β*H-ET_B_:ET_B_^sl/sl^ rats (Taylor et al., 2003). Moreover, female D*β*H-ET_B_:ET_B_^sl/sl^ rats on a high-salt diet (8% NaCl) have significantly greater plasma concentrations of ET-1 compared with male D*β*H-ET_B_:ET_B_^sl/sl^ rats. In both female D*β*H-ET_B_:ET_B_^sl/sl^ rats, tempol (a superoxide dismutase mimetic) initially attenuated the salt-dependent increase in blood pressure but after 2 weeks of treatment was ineffective (Sullivan et al., 2006). These data suggest that reactive oxygen species (ROS) are critical for the early stages of the salt-dependent increases in blood pressure, and it is hypothesized that the female sex does not protect against hypertension in the D*β*H-ET_B_:ET_B_^sl/sl^ model similar to other models of hypertension due to increased circulating ET-1 and superoxide production (Sullivan et al., 2006). Taken together, these data suggest that sex differences exist in D*β*H-ET_B_:ET_B_^sl/sl^ rats and that these involve an interaction with dietary salt.

Deoxycorticosterone acetate (DOCA)-salt-induced hypertension is a common model to study mechanisms of salt-dependent blood pressure control. ET_A_, as well as ET_A_/ET_B_ receptor antagonists, can attenuate the development of hypertension in DOCA models (Allcock et al., 1998; Li et al., 1994). In D*β*H-ET_B_:ET_B_^sl/sl^ rats, DOCA and salt (1% NaCl in drinking water) resulted in a significant increase in systolic pressure within a week of DOCA implantation, whereas the transgenic and heterozygous controls presented with an increase in pressure after 4 weeks (Matsumura et al., 2000c). ET_A_ receptor antagonism suppressed DOCA-salt-induced hypertension in D*β*H-ET_B_:ET_B_^sl/sl^ rats and transgenic control rats (Matsumura et al., 2000b). After 4 weeks of DOCA-salt, there was marked renal and vascular pathology, including hypertrophy, but this was attenuated with ET_A_ antagonism (Matsumura et al., 2000a). These data suggest that functional ET_B_ receptors are protective against the development of DOCA-salt-dependent hypertension; however whether this is also true for female rats remains to be determined.

## VI. Phenotypes of Cell-Specific Genetic Modifications in the Endothelin System

### A. Cell-Specific Overexpression of Endothelin-1

In many pathologic states, circulating concentrations of ET-1 are elevated. For example, ET-1 is elevated in pulmonary hypertension (Stewart et al., 1991a), myocardial infarction (Stewart et al., 1991b), stroke (Franceschini et al., 2001; Volpe and Cosentino, 2000), abdominal aortic aneurysms (Flondell-Site et al., 2010), and chronic kidney disease patients (Lebel et al., 1994). To test mechanistic questions about the source of elevated ET-1 levels, a number of cell-specific overexpression ET-1 transgenic mice have been developed.

#### 1. Overexpression of Endothelin-1 in Endothelium.

To determine the effects of increasing endothelial ET-1 production on vascular physiology, a transgenic mouse overexpressing the human preproET-1 was generated using the promoter and enhancer regions of the endothelium-specific tyrosine kinase receptor (Tie-2) (Amiri et al., 2004). These mice were named TG or eET-1 mice and present with a sevenfold increase in circulating ET-1 compared with wild-type littermate controls; however, heart rate and blood pressure were similar to controls (Amiri et al., 2004). eET-1 mice displayed endothelial dysfunction, because vasodilation of mesenteric resistance arteries was significantly attenuated that was likely due to an increase in NADPH oxidase activity and ROS production (Amiri et al., 2004). Moreover, a DNA microarray performed with mesenteric arteries from eET-1 and control mice determined that eET-1 mice have increased expression of genes critical for lipid metabolism (Simeone et al., 2011). This finding led to the hypothesis that endothelial ET-1 may participate in the development of atherosclerosis.

To determine if increases in ET-1 in the endothelium promotes atherosclerosis, the eET-1 mouse was bred onto the atherosclerosis prone apolipoprotein E KO (Apoe^−/−^) mouse (Li et al., 2013). When placed on a chronic high-fat diet, eET-1, Apoe^−/−^, and Apoe^−/−^eET-1 mice all had an increase in the atherosclerotic lesion size of the descending thoracic aorta compared with wild-type control mice; however, Apoe^−/−^eET-1 mice had a significantly higher lesion size compared with eET-1 or Apoe^−/−^ mice. Further investigation revealed that an increase in endothelial ET-1 promotes atherosclerosis and abdominal aortic aneurysms by decreasing high-density lipoprotein, increasing oxidative stress, and increasing inflammatory cell infiltration (Li et al., 2013).

A second endothelial cell-specific overexpression of mouse ET-1 driven by the tyrosine-protein kinase receptor 1 promoter was also produced. These mice, named TET-1, have an increase in brain vasculature ET-1 (as determined by in situ hybridization and semiquantitative PCR) compared with nontransgenic littermates (Leung et al., 2004). Interestingly, these mice also have an elevated systolic and mean arterial blood pressure (Leung et al., 2004), but similar to the eET-1 mice (Amiri et al., 2004) have aortic and mesenteric artery endothelial dysfunction. Thus, there are two unique models that can be used to test mechanistic questions about elevated endothelial ET-1 production.

In chronic kidney disease patients, plasma ET-1 concentrations are elevated (Lebel et al., 1994). Because CKD patients are often anemic, erythropoietin is given to help achieve normal hematocrit levels (Lariviere and Lebel, 2003; Drueke et al., 2006; Unger et al., 2010). However, as a consequence, ET-1 levels also are further increased (Carlini et al., 1993) and thought to perhaps lead to elevated blood pressure in CKD patients (Barhoumi et al., 2014). Barhoumi et al. (2014) hypothesized that exercise may help prevent the increase in blood pressure and vascular pathologies associated with erythropoietin treatment in CKD and end stage renal disease (ESRD). ET-1 mice and controls were given erythropoietin and then either subjected to swimming exercise training or remained sedentary. Erythropoietin had no significant effect on blood pressure or vascular function in the wild-type mice (Barhoumi et al., 2014). In contrast, the eET-1 mice experienced significant increases in systolic pressure, vascular media/lumen ratio, NADPH oxidase activity, and ROS production. Interestingly, all of this was prevented with exercise training, although ET-1 levels remained elevated (Barhoumi et al., 2014). These data suggest that exercise may prevent ET-1-dependent adverse cardiovascular events in CKD and ESRD patients after erythropoietin treatment. Furthermore, the use of receptor antagonists for cardioprotection is ESRD patients should be explored especially because the risk of fluid retention would not be a serious problem.

#### 2. Overexpression of Endothelin-1 in Cardiomyocytes.

In the setting of congestive heart failure (CHF), circulating ET-1 is elevated (Stewart et al., 1992) along with increased myocardial expression of ET-1, ET_A_, and ET_B_ receptors (Picard et al., 1998; Pieske et al., 1999). To determine if this increase in the ET-1 axis is pathologic, a mouse was developed to conditionally overexpress ET-1 in the myocardium. This mouse was developed using the *α*-myosin heavy chain promoter-dependent cardiac-specific tetracycline-regulated gene expression system to drive human ET-1 expression (Yang et al., 2004). Once tetracycline was given to the adult animals to induce cardiac ET-1 overexpression, there was a 10-fold higher concentration of ET-1 in the heart, but no significant difference was detected in circulating ET-1 compared with control mice (Yang et al., 2004). These elevated cardiac ET-1 mice presented with severe heart inflammation, hypertrophy leading to dilated cardiomyopathy, CHF, and death within 5 weeks of induction. Moreover, treatment of these mice with the ET_A_/ET_B_ blocker LU420627 (but not the ET_A_ blocker, LU135252), inhibited leukocyte recruitment and preserved cardiac function, although this response was only transient and mice began to die at 8 weeks post-ET-1 overexpression (Yang et al., 2004). Thus, this study provides evidence that cardiac ET-1 and potentially the ET_B_ receptor are putative therapeutic targets in the setting of CHF.

#### 3. Overexpression of Endothelin-1 in Astrocytes.

Stroke patients have elevated plasma and cerebrospinal fluid concentrations of ET-1 (Volpe and Cosentino, 2000; Franceschini et al., 2001). Ischemia/reperfusion leads to blood-brain barrier injury, including an increase in vascular permeability and edema (Cole et al., 1991; Yang and Betz, 1994). Furthermore, astrocytes after hypoxia/reperfusion have elevated levels of ET-1 mRNA (Tsang et al., 2001; Lo et al., 2005); however, it is unclear whether this could be protective or pathologic. To answer this question, an astrocyte-specific ET-1 overexpression mouse was created (named GET-1 mice, using the glial fibrillary acidic protein promoter) (Lo et al., 2005). These mice develop normally and have a normal mean arterial blood pressure under baseline conditions; however, after middle cerebral artery occlusion-induced cerebral ischemia, GET-1 mice displayed a larger brain infarct size, increased brain swelling, and increased brain water content, although survival was similar to littermate controls (Lo et al., 2005). Middle cerebral artery occlusion-induced cerebral ischemia resulted in a threefold increase in ET-1 in the ipsilateral hemisphere in the control mice but a 25-fold increase in ET-1 in the GET-1 mice (Lo et al., 2005). Thus, increases in astrocyte ET-1 during ischemia/reperfusion leads to severe blood-brain barrier injury. Likewise, increases in astrocyte ET-1 production results in edema and vasospasm after subarachnoid hemorrhage; however, this can be prevented with ET_A_ and vasopressin V1a receptor antagonism in GET-1 mice (Yeung et al., 2013).

Chronic pain, for example inflammatory pain or neuropathic pain, can be caused by or often leads to tissue injury. Although, in animal models, exogenous ET-1 has been determined to induce pain peripherally (Piovezan et al., 2000) or inhibit pain if injected centrally (Yamamoto et al., 1994); the putative effect of ET-1 from astrocytes specifically is unclear. Centrally, astrocyte ET-1 has also been suggested to alleviate neuropathic pain (Hung et al., 2014). GET-1 and control mice were subjected to sciatic nerve ligation-induced neuropathic pain, and pain-related to allodynic and hyperalgesic behaviors were determined. An increase in astrocyte ET-1 leads to pain alleviation for over 21 days after the sciatic nerve ligation-induced neuropathic pain, and this was likely mediated by the ET_A_ receptor (Hung et al., 2014). Likewise, astrocyte ET-1, but not endothelial cell-derived ET-1 (TET-1 mice), can alleviate inflammatory pain (Hung et al., 2012). Taken together these studies suggest that increases in astrocyte ET-1 may be harmful in the setting of stroke but may alleviate chronic pain.

### B. Cell-Specific Deletion of the Endothelin System

#### 1. Neuron-specific Endothelin-1 Knockout Mice.

To generate a neuron-specific ET-1 KO mouse, ET-1 floxed alleles were deleted by Cre expression driven by the synapsin 1 promoter (Hasue et al., 2005). Neuron-specific ET-1 KO mice had a greater sensitivity to acute nociceptive stimuli, a greater degree of mechanical allodynia in spinal nerve ligation-induced NP, and reduced stress-induced analgesia (Hasue et al., 2005). These data suggest that neuronal ET-1 could also be involved in suppression of pain. Given the strong evidence that ET-1/ET_A_ receptors contribute to sensory nerve pain, the apparent contrast of central versus peripheral ET_A_ receptors in the pain pathway require further investigation.

#### 2. Cardiomyocyte Endothelin-1 Knockout.

Cardiomyocyte ET-1 KO mice were produced using an alpha-myosin heavy chain promoter to drive Cre expression (Shohet et al., 2004). These mice appear healthy into adulthood. In isolated cardiomyocytes, there was a significant 75% reduction in ET-1. To induce cardiac hypertrophy, littermate control and cardiomyocyte ET-1 KO mice were treated chronically with exogenous tri-iodothyronine (Shohet et al., 2004). The KO mice had a 57% reduction in cardiac hypertrophy compared with control mice, suggesting that cardiac ET-1 contributes to cardiac hypertrophy in his model (Shohet et al., 2004).

Although reduction of ET-1 was beneficial in the context of cardiac hypertrophy, the same may not be true in terms of the aging heart. Cardiomyocyte ET-1 KO mice at 2 months of age had normal left ventricular function compared with controls (Zhao et al., 2006); however, at 6 months of age, left ventricular systolic function had declined, and by 11 months many of the KO mice had died (Zhao et al., 2006). Aged KO mice had dilated heart chambers and increased cardiac fibrosis consistent with heart failure. Furthermore, if young KO mice were challenged with transverse aortic constriction, there was an accelerated development of heart failure and loss of cardiomyocytes via apoptosis (Zhao et al., 2006). Thus, from this study, it is evident that cardiac ET-1 is critical for cardiac function and cardiomyocyte survival in aging or stressed mice.

#### 3. Cardiomyocyte Endothelin A Receptor Knockout.

Cardiomyocyte ET_A_ KO mice were also developed using the alpha-myosin-heavy chain promoter to drive Cre (Kedzierski et al., 2003). These KO mice developed normally into adulthood and had normal cardiac size and function. Moreover, when challenged with angiotensin II or isoproterenol to induce cardiac stress, cardiomyocyte ET_A_ KO mice and littermate controls had similar levels of cardiac hypertrophy, and both genotypes had a similar increase in peak velocity and decrease in ejection time (Kedzierski et al., 2003). Thus, at least in young mice (6- to 7-week-old mice), cardiac function is normal in mice lacking cardiac ET_A_ receptors. Whether this is also true for aging mice remains to be determined.

Cold stress, such is experienced during cold winter months, is associated with oxidative stress and cardiac dysfunction including hypertrophy. To determine if ET_A_ receptors are involved in cardiac dysfunction during cold stress, cardiomyocyte ET_A_ KO mice and controls were exposed to ambient or 4°C temperatures for 2 and 5 weeks (Zhang et al., 2012). Cardiomyocyte ET_A_ KO mice were protected against cold stress-induced cardiac fibrosis and cardiac dysfunction. Moreover, control mice had an increase in ROS production, caspase-3 activity, and increased myocardial apoptosis after cold-stress that was absent in the cardiomyocyte ET_A_ KO mice (Zhang et al., 2012). These data suggest that an increase in ET_A_ activation induced by cold stress exposure leads to cardiac dysfunction.

#### 4. Smooth Muscle Endothelin A Receptor Knockout.

ET_A_ receptors are abundantly expressed throughout the vascular smooth muscle, where they lead to vasoconstriction. Deletion of ET_A_ receptors from the smooth muscle was developed using smooth muscle protein 22-Cre mice (Donato et al., 2014). These mice had a number of developmental abnormalities, similar to the whole body ET_A_ KO mice (above). There was decreased viability, which may be attributed to growth retardation, mandibular maldevelopment, cranial vascular malformations, abnormal thymus, pericardial cystic space, and thickened aortic arch vessels (Donato et al., 2014). However, some smooth muscle ET_A_ KO (SM-ET_A_KO) mice managed to survive into adulthood, and as adults, there were no differences in body, organ, or adipose mass or blood glucose. When challenged with ET-1 in the presence of ET_B_ receptor blockade, SM-ET_A_KO mice had blunted femoral artery vasoconstriction. Although baseline mean arterial pressure was similar between the genotypes, floxed control mice had a significant increase in mean arterial pressure in response to a bolus infusion of ET-1 that was absent in the SM-ET_A_KO mice (Donato et al., 2014). Thus from this study it is evident that smooth muscle ET_A_ receptors are critical for proper development, and contribute to the regulation of arterial pressure in adults.

#### 5. Smooth Muscle Endothelin-1 Knockout.

Although the endothelium is a major source of ET-1 production, smooth muscle cells also produce ET-1, and it is hypothesized that smooth muscle ET-1 (SM ET-1 KO) may be involved in the pathogenesis of PAH. To test this hypothesis, ET-1 has been deleted from the smooth muscle using sm22-Cre (Kim et al., 2015). Kim et al. (2015) did not comment on any developmental abnormalities in the SM ET-1KO mice. At baseline, floxed control and SM ET-1KO mice had similar right ventricular systolic pressure (RVSP); however, exposure to hypoxia (10% oxygen for 21–24 days) resulted in a significant increase in RVSP in both genotypes, albeit it was relatively attenuated in the SM ET-1KO mice (Kim et al., 2015). Circulating ET-1 levels were increased in a similar manner between the floxed and SM ET-1 KO mice exposed to hypoxia illustrating that most circulating ET-1 is from the endothelium and not the smooth muscle (Kim et al., 2015). Thus, the improved RVSP in response to hypoxia in the SM ET-1KO mice suggests that smooth muscle ET-1 plays a role in the pathogenesis of PAH.

#### 6. Endothelial Endothelin B Knockout.

Given that endothelial ET_B_ receptor activation stimulates NOS3 NO production, one might expect that endothelial cell ET_B_ deletion would increase blood pressure. To delete ET_B_ from the endothelium, a Tie2-Cre mouse was used leading to loxP recombination and deletion of exons 3 and 4 of the ET_B_ receptor (Bagnall et al., 2006). Endothelial cell ET_B_ KO mice (ECET_B_KO) had increased circulating ET-1 levels, again confirming the importance of the endothelial ET_B_ for ET-1 regulating circulating ET-1 concentrations (Kelland et al., 2010b). ECET_B_KO mice also had impaired aortic vasodilation to the ET_B_ agonist sarafotoxin 6c (S6c) and acetylcholine that was related to a decrease in NO bioavailability. Moreover, exposure to hypoxia leads to an exaggerated muscularization and remodeling of pulmonary arteries and a greater increase in systolic right ventricular pressure in ECET_B_KO mice (Kelland et al., 2010a). Although endothelial dysfunction was evident in the ECET_B_KO mice, they were normotensive and had similar blood pressures to floxed control mice on chronic high-salt diets (Bagnall et al., 2006). These data suggest that endothelial ET_B_ receptors mediate vasodilation and limit vascular remodeling but may not necessarily be involved in long-term blood pressure control. However, these mice were developed on a salt-sensitive background strain and so both control and ECET_B_KO mice became hypertensive on the high-salt diet. Additional studies on a salt-resistant strain of mouse are needed.

In humans, the portal vein expresses about 57%:43% ET_A_: ET_B_ receptors; however, in portal veins from cirrhotic livers, there was shift to 90%:10% (Ling et al., 2012). Thus, the ECET_B_KO mice may be an appropriate model to test mechanistic questions about portal hypertension and cirrhosis. The liver of the ECET_B_KO mouse had a reduced sinusoid diameter and red blood cell congestion in the intrahepatic branches of the portal vein (Ling et al., 2012). Taken together these data suggest that endothelial ET_B_ receptors tonically dilate the portal vein and that ET_A_ antagonism may be beneficial in cirrhotic patients.

#### 7. Endothelial Endothelin-1 Knockout.

To further evaluate the physiologic role of ET-1 in the endothelium, a vascular endothelial cell ET-1 KO mouse (VEETKO) was developed, again using Tie2-Cre to drive ET-1 deletion from the endothelium (Kisanuki et al., 2010). Deletion of ET-1 from the endothelium resulted in a 65–80% reduction in circulating ET-1 and ET-1 content in the major organs (Kisanuki et al., 2010). Yet, acute blood pressure responses to captopril, angiotensin II, phenylephrine, bradykinin, l-NAME, or ET-1 were similar to floxed controls, suggesting that the lacking endothelial ET-1 does not affect these paracrine/autocrine signaling systems (Kisanuki et al., 2010). Although it was initially determined that VEETKO mice have a significantly lower blood pressure than floxed controls (Kisanuki et al., 2010; Adiarto et al., 2012), others have reported that blood pressure is similar between these genotypes (Heiden et al., 2014). Thus, it remains controversial if basal blood pressure is altered by the lack of endothelial ET-1. However, ANGII infusion leads to a similar increase in blood pressure between VEETKO and floxed mice, but cardiac hypertrophy and fibrosis was reduced in the ANGII-infused VEETKO mice (Adiarto et al., 2012). Tissue growth factor, TGF*β*, collagen I and III were reduced in ANGII-infused VEETKO mice, suggesting that endothelial-derived ET-1 is a critical mediator in ANGII-dependent fibrosis (Adiarto et al., 2012). In contrast, loss of endothelial-derived ET-1 appears protective in a transaortic constriction model of cardiac hypertrophy, where VEETKO mice with transaortic constriction had reduced cardiac function (Heiden et al., 2014). The role of endothelial-derived ET-1 in cardiac hypertrophy likely depends on a variety of factors, including the level of ET receptor expression in each cell type.

ET-1 is reported to be increased in acute kidney injury (AKI) and may be part of the pathogenesis of the progression of a variety of acute as well as chronic kidney diseases (Wilhelm et al., 1999). To determine if elevated ET-1 in AKI is derived from the endothelium, ischemia/reperfusion injury experiments were performed in VEETKO and floxed control mice (Arfian et al., 2012). Although, ischemia/reperfusion injury resulted in increased oxidative stress, inflammation, and tubular and small renal artery injury, these pathologies were attenuated in the VEETKO mice (Arfian et al., 2012). Likewise, exposure to hypoxia or 2 weeks of high-salt diet (4% NaCl) leads to an increase in glomerular ET-1, oxidative stress, and kidney injury in floxed control mice, but this was attenuated in VEETKO mice (Heimlich et al., 2015). These data suggest that endothelial ET-1 is pathologic in states of increased oxidative stress including AKI.

#### 8. Intestinal Epithelium-specific Endothelin-2 Knockout.

Conditional intestinal epithelium-specific ET-2 KO mice (driven by Cre expression under the control of the villin promoter) were born without any observable abnormalities and had normal survival (Chang et al., 2013). These intestinal ET-2 KO mice had normal fat absorption and excretion of fats and carbohydrates, suggesting that unlike the global ET-2^−/−^ mice that suffered from internal starvation, intestinal ET-2 derived from epithelial cells is not essential under normal conditions (Chang et al., 2013). The specific cellular source of ET-2 that regulates metabolism will need further investigation.

#### 9. Neuron-specific Endothelin-2 Knockout.

Neuron-specific ET-2 KO mice were produced with Cre driven by the nestin promoter (Chang et al., 2013). These mice were born healthy, with no growth defects, and had a normal core body temperature unlike the ET-2^−/−^ mice. Thus, the body temperature defect displayed by ET-2^−/−^ mice is not due to ET-2 deletion from the nervous system (Chang et al., 2013), but the precise origin has yet to be determined.

#### 10. Collecting Duct Endothelin-1 Knockout.

The inner medullary collecting ducts have the highest density of ET_B_ receptors in the body and produce a large amount of ET-1. To determine the physiologic role of the collecting duct ET-1 system, a series of studies were performed by deleting various aspects of this system from the collecting duct. Mice with lox-P sites surrounding genes of ET-1, ET_A_, and ET_B_ receptors were bred with AQP2-Cre mice (Nelson et al., 1998). AQP2 is a water channel expressed in the principal cell of the collecting duct, testes, vas deferens, and epididymis (Nelson et al., 1998). Thus, breeding floxed mice with AQP2-Cre-positive dams leads to lines of principal cell, collecting duct-specific KO mice.

Deletion of ET-1 from the CD resulted in a salt-sensitive blood pressure phenotype. On a normal salt diet, CDET-1KO mice had significantly higher systolic blood pressure compared with floxed controls mice (Ahn et al., 2004). On a high-salt diet (2% sodium) CDET-1KO mice had an even greater increase in blood pressure, whereas floxed controls had no change, demonstrating a salt-sensitive phenotype. CDET-1KO mice had sodium retention, and this could be ameliorated with amiloride or furosemide. However, diuretic treatment only reduced the salt-sensitive blood pressure and did not affect the higher blood pressure while on a normal salt diet (Ahn et al., 2004). Plasma aldosterone, plasma renin activity, and plasma ET-1 concentrations were similar between the genotypes regardless of the salt in the diet, which was surprising considering the CDET-1KO mice are retaining sodium and it would be expected that the renin-angiotensin-aldosterone system would be suppressed in these mice. Interestingly, blood pressure is decreased in the CDET-1KO mice with angiotensin receptor blockade or mineralocorticoid receptor antagonism with spironolactone (Ge et al., 2008b). Thus, part of the hypertension in the CDET-1KO mouse is due to relative renin-angiotensin-aldosterone system overactivity, and these data suggest that CD ET-1 may regulate renal renin production (Ge et al., 2008b).

As expected, CDET-1KO mice had reduced ET-1 excretion, and although ET-1 excretion increased significantly in the floxed animals with high-salt diet, this was not observed in the CDET-1KO mice. These data suggest that the CD is a major source of urinary ET-1 and that high salt feeding significantly increases CD-derived ET-1 production and excretion (Ahn et al., 2004).

Given that ET-1 can stimulate CD NO production (Stricklett et al., 2006; Hyndman et al., 2012), it was tested whether CDET-1KO mice have an impaired inner medullary NO system. With both normal salt and high salt feeding, CDET-1KO mice have reduced inner medullary NOS1 and NOS3 activity and reduced urinary nitrite/nitrate excretion (Schneider et al., 2008). Furthermore, increasing renal perfusion pressure in anesthetized mice resulted in reduced nitrite/nitrate, sodium, and water excretion in CDET-1KO mice compared with controls (Schneider et al., 2008), further demonstrating that CD ET-1 promotes NO production and natriuresis and diuresis. Thus, the CD ET-1 system is critical for limiting increases in blood pressure under both normal and high salt feeding.

CD ET-1 also plays a role in the regulation of water excretion. Floxed control and CDET-1KO mice were challenged with ddAVP, the vasopressin receptor-2 agonist (Ge et al., 2005a). Chronic ddAVP infusion into CDET-1KO mice resulted in a greater increase in urine osmolality for the first 3 days of infusion compared with floxed controls. Moreover, freshly isolated CDs from CDET-1KO mice had elevated adenylyl cyclase 5/6 expression (Strait et al., 2007) and greater AVP-dependent cAMP production compared with CDs from floxed controls (Ge et al., 2005a). These data suggest that CD ET-1 promotes diuresis.

ET-1 can increase CD PGE_2_ production that can also lead to natriuresis and diuresis. Interestingly, CDET-1KO mice have elevated PGE_2_ excretion under both normal and high-salt diets compared with floxed controls (Ge et al., 2007), suggesting that PGE2 is increased to compensate for the lack of CD ET-1. However, blocking prostaglandins chronically with indomethacin resulted in a more concentrated urine from the CDET-1KO mice compared with littermate control, although there was a similar increase in blood pressure (Ge et al., 2007). These data suggest that the compensatory increase in CD PGE_2_ promotes diuresis in CD ET-1KO mice but does not affect blood pressure. It can be concluded that CD ET-1 is a key regulator for both sodium and water homeostasis, which is critical for homeostatic regulation of blood pressure.

#### 11. Collecting Duct Endothelin A Receptor Knockout.

The ET_A_ receptor was deleted from the CD, CDET_A_KO, and these mice had normal development and aging (Ge et al., 2005b). On a normal salt or 2% high sodium diet, CDET_A_KO mice and floxed control mice had similar blood pressure, sodium excretion, plasma creatinine, and plasma renin activity and concentration (Ge et al., 2005b). From this it was concluded that the CD ET_A_ receptor is not critical for regulation of sodium excretion and blood pressure control. However, CDET_A_KO mice had significantly higher plasma AVP concentration, whereas freshly isolated CDs from CDET_A_KO mice had reduced AVP-dependent cAMP production. Furthermore, CDET_A_KO mice have reduced concentrating ability when challenged with ddAVP (Ge et al., 2005b). Interestingly, ET_A_ receptor antagonists can produce fluid retention, most likely due to renal tubular ET_A_ receptors, and so together, these data suggest that CD ET_A_ receptor activation contributes to the urine concentrating mechanisms, although the signaling mechanisms are yet to be determined.

#### 12. Collecting Duct Endothelin B Receptor Knockout.

The CD has a very high density of ET_B_ receptors. Deletion of the ET_B_ receptor from the CD resulted in an increase in systolic and diastolic blood pressure that was further increased with high salt feeding (Ge et al., 2006). CDET_B_KO mice also had reduced plasma renin activity and reduced aldosterone excretion, which is consistent with having sodium retention and possibly an expanded plasma volume. Moreover, CDET_B_KO mice had a delayed natriuretic response to an acute sodium load. Although, CDET_B_KO mice have an elevated blood pressure, it is not as high as CDET-1KO mice, suggesting that the natriuretic effects of ET-1 may involve non-CD ET receptors (Ge et al., 2006). ET receptors are also expressed on the vasa recta and other tubular segments (see Kohan et al., 2011), thus it is possible that CD ET-1 can act in a paracrine manner to regulate sodium excretion.

#### 13. Collecting Duct Endothelin A/B Receptor Knockout.

To further determine the contribution of the CD ET receptors to fluid-electrolyte balance and blood pressure control, both receptors were genetically deleted from the CD. CDET_A/B_KO mice also display an elevated systolic pressure while on a normal salt diet that was greater than the CDET_B_KO mouse but similar to the CDET-1KO mouse (Ge et al., 2008a). With high salt diet, there was a significant increase in systolic pressure compared with floxed control mice, and this was similar to that observed in CDET-1KO mice but significantly higher than CDET_B_KO (∼15 mmHg) or CDET_A_KO (∼1 mmHg) mice (Ge et al., 2008a). Sodium excretion was blunted on the first 2 days of high salt diet feeding, suggesting sodium retention in the CDET_A/B_KO mouse compared with controls. From this study, it is evident that both ET_A_ and ET_B_ activation can lead to natriuresis/diuresis and that autocrine and paracrine actions of ET-1 are critical for fluid-electrolyte balance and blood pressure control.

In the CD, the epithelial sodium channel (ENaC) is a rate-limiting step in the reabsorption of sodium. Exogenous ET-1 decreases ENaC open probability and activity (Bugaj et al., 2008), demonstrating a direct tubular action of ET-1 on sodium homeostasis. To determine which CD ET receptors may be mediating this ET-1-dependent decrease in sodium reabsorption, ENaC activity in acutely isolated cortical CD was measured in the CDET_A_KO, CDET_B_KO, and CDET_A/B_KO mice. CDET_A_KO mice had similar, low ENaC activity that was not different from wild-type controls, and as the salt in the diet increased, there was a similar expected decrease in ENaC activity. In essence, CDET_A_KO mice and controls had the same, normal suppression of ENaC activity with an increase in salt intake (Bugaj et al., 2012). In contrast, normal salt fed CDET_B_KO and CDET_A/B_KO mice presented with a significantly greater ENaC activity compared with control and CDET_A_KO mice. Furthermore, increasing dietary sodium intake did not suppress ENaC activity in these mice, thus remaining inappropriately high. From this study, it is evident that CD ET_B_ receptor activation is critical for inhibiting ENaC activity and thus sodium reabsorption. Furthermore, this signaling cascade involves CD NOS1 activity, because CDNOS1KO mice fail to suppress ET-1-dependent ENaC activity (Hyndman et al., 2015).

#### 14. Cardiomyocyte, Smooth Muscle, Nephron, and Collecting Duct Endothelin A Receptor Knockout.

ET_A_ receptor blockade is proposed to be beneficial in a number of cardiovascular/renal diseases; however, there is often significant fluid retention reported with their use that can be a clinically significant problem. To further determine if renal epithelial ET_A_ receptors are critical for promoting diuresis, an inducible nephron ET_A_ KO mouse was developed using a Pax8-rtTA and LC-1 transgenic animals. Floxed ET_A_ mice were bred with Pax8-rtTA/LC-1 mice and deletion of nephron ET_A_ was initiated by doxycycline treatment in adulthood (Stuart et al., 2012). Nephron ET_A_ KO mice had a modest fluid retention while on a high-salt diet but no appreciable change in blood pressure or sodium excretion (Stuart et al., 2012). Thus, nephron ET_A_ receptors likely do not play a predominant role in the regulation of sodium homeostasis or blood pressure control and partially contribute to fluid retention seen with ET_A_ antagonists. Importantly, this study suggests a role for extra-nephron ET_A_ receptors mediating ET_A_ antagonist-dependent fluid retention.

To determine which of these potentially extra-nephron ET_A_ receptors may be contributing to the regulation of body fluids, a comparative study among the cardiomyocyte ET_A_ KO, SM-ET_A_ KO, nephron ET_A_KO, CDET_A_KO, and floxed ET_A_ control mice was performed (Stuart et al., 2013). All these mice were fed a high 3.5% sodium diet for 7 days followed by high-salt diet plus 2 weeks of ET_A_ antagonism (ambrisentan or atrasentan). ET_A_ antagonism resulted in a significant increase in body weight, total body water, and extracellular fluid in the floxed control and cardiomyocyte ET_A_ KO mice only. Nephron and CD ET_A_KO mice were protected to a similar degree from the ET_A_-antagonist-dependent fluid retention, suggesting it is antagonism of the CD ET_A_ receptor that leads to fluid retention. The SM-ET_A_KO mice also were partially protected from this fluid retention. These data suggest that ET_A_-antagonist induced fluid retention is due to CD ET_A_ receptors and SM-ET_A_ receptor, but not likely cardiomyocyte ET_A_ receptors (Stuart et al., 2013).

#### 15. Podocyte Endothelin A/B Receptor Knockout.

Podocytes have a key role in glomerular function: with endothelial cells of the glomerular capillary loop and the glomerular basement membrane they form a filtration barrier. In mice, podocytes express both receptors. Diabetic nephropathy was induced experimentally in mice with podocyte-specific double knockout of both receptors. This deletion resulted in less albuminuria and protection from glomerulosclerosis and podocyte loss (Lenoir et al., 2014). These results suggest that in mice at least, the ET_B_ receptor may play as important a role as does the ET_A_ receptor in diabetes, whereas previous studies have suggested the latter was the main subtype mediation the deleterious actions of ET-1 (Chandrashekar and Juncos, 2014).

## VII. Role of Endothelin in Human Pharmacology Deduced from Experimental Medicine Studies

### A. Clinical Pharmacology of Peptide Endothelin Receptor Agonists and Antagonists

ET-1 and ET-3 (along with various ET receptor antagonists) have been most widely used over the years to define their role and that of the ET_A_ and ET_B_ receptors in human physiology and disease (Haynes and Webb, 1994; Haynes et al., 1995a,b; MacIntyre et al., 2010). The ET_B_ receptor agonist S6c has been used in a number of clinical studies (Strachan et al., 1995, 2000; Ferro et al., 2002; Newby et al., 2002) and probably carries the same risks as those associated with other agonists.

In humans, brachial artery infusion of ET-1 causes a sustained, dose-dependent reduction in forearm blood flow (Clarke et al., 1989; Haynes et al., 1995a,b). Low doses of the ET_B_-selective agonists ET-3 and S6c also produce vasoconstriction when infused into the brachial artery in keeping with vascular ET_B_ receptors mediating at least part of the functional response to ET-1 in these vessels (Haynes et al., 1995a,b). ET_B_ receptor agonists can also produce transient forearm vasodilation that is NO mediated (Newby et al., 2002).

Both ET-l (Clarke et al., 1989) and S6c (Haynes et al., 1995a,b) cause a sustained constriction of human dorsal hand veins in vivo, suggesting that both vascular ET_A_ and ET_B_ receptors contribute to venoconstriction to ET-1 in humans. This is further supported by the capacity for the selective ET_A_ receptor antagonist BQ123 to attenuate the in vivo venoconstriction to ET-l but not S6c (Strachan et al., 1995). Thus, the part of the ET-1 response that is not inhibited by BQ123 is likely to be ET_B_ receptor mediated. In addition, venoconstriction to both ET-1 (Haynes and Webb, 1993) and S6c (Strachan et al., 1995) is markedly attenuated by endothelial ET_B_-mediated production of vasodilator substances, prostacyclin, and NO.

In humans, brachial artery administration of Big ET-1 leads to a dose-dependent forearm vasoconstriction that is completely blocked by the ECE inhibitor phosphoramidon, suggesting that the effects of the precursor are mediated through its conversion to the mature peptide by ECE (Haynes and Webb, 1994). The blockade of constriction to Big ET-l by phosphoramidon is unlikely to have been due to inhibition of ET receptor binding, because vasoconstriction to ET-1 was unaffected by phosphoramidon and because conversion to ET-1 and its C-terminal fragment was confirmed in plasma samples taken from the veins draining the infused forearm (Plumpton et al., 1995). As circulating blood exhibits little ECE activity (Watanabe et al., 1991), conversion of Big ET-1 in the forearm probably occurs via vascular ECE situated within the forearm blood vessels. The difference in potency between Big ET-1 and ET-1 and the ratio of C-terminal fragment to Big ET-1 in venous blood, both indicate that local ECE converts ∼10% of luminally presented Big ET-1 to ET-1, consistent with ∼10% conversion of exogenous Big ET-1 by cells expressing the ECE-1 gene (Xu et al., 1994). To date the therapeutic strategy of combined ECE/NEP remains and unproven concept (see section II.A.8), particularly as NEP inhibition alone is associated with vasoconstriction and has not proven effective when exploited clinically (Ferro et al., 1998). Further clinical studies are needed with inhibitors that are selective for ECE alone.

Such agonist studies have proven valuable in helping to define the site of production of ET-1 and its mediators of action. However, they may not adequately represent the effects of a hormone, the actions of which are largely autocrine/paracrine, and inhibition of the production, or actions of ET-1 may better define its physiologic and pathologic effects. In this respect, ET receptor antagonists have proven to be useful tools in defining the role of ET in health and disease. The distinction between selective and mixed antagonists is discussed in section IV. Highly selective peptide ET_A_ antagonists such BQ123 and the ET_B_-selective BQ788 have provided the clearest evidence for defining the role of the two receptors. In addition, a number of antagonists have been studied with a range of relative affinities for ET_A_ over the ET_B_ subtype or as well as mixed antagonists such as the orally active bosentan (Dhaun et al., 2007b).There now exist a number of ET receptor antagonists that have or are being explored in a variety of clinical conditions (see Table 1).

With respect to systemic hemodynamics in healthy man, selective ET_A_ receptor antagonism with BQ123 is associated with peripheral vasodilation (Verhaar et al., 1998) and a reduction in blood pressure (BP) (Spratt et al., 2001) and selective ET_B_ receptor antagonism (BQ788) with peripheral vasoconstriction (Verhaar et al., 1998) and a pressor response (Strachan et al., 1999). This suggests that endogenous ET-1 contributes to the maintenance of vascular tone and BP via the ET_A_ receptor and the balance of ET_B_ receptor function favors activation of the endothelial over the vascular smooth muscle ET_B_ receptor.

### B. Clinical Application of Nonpeptide Endothelin Receptor Antagonists

#### 1. Chronic Heart Failure.

Many of the early trials using ET receptor antagonists were carried out in patients with chronic heart failure (CHF; Table 5). These were based on compelling preclinical (Sakai et al., 1996; Kobayashi et al., 1999) and clinical data (Pieske et al., 1999) that showed an upregulation of the ET system in CHF. Furthermore, the high blood levels of ET-1, observed in both animal models and patients with CHF, correlated with poor functional state and lower survival (Motte et al., 2006). Although the initial acute dosing studies using both selective ET_A_ and mixed ET_A/B_ approaches were encouraging (Kiowski et al., 1995; Cowburn et al., 2005), these used hemodynamic end points, which are a poor surrogate of benefit on clinical outcomes. Longer term outcome studies have been disappointing in respect to both of these approaches.

To date, four large multicenter double-blind trials have been completed. Bosentan was evaluated in both the REACH-1 and ENABLE studies. The former was terminated early because of side effects (elevated liver enzymes) attributed to the high dose of drug used (up to 500 mg twice daily). Consequently, the latter study used a lower dose of drug (125 mg twice daily) with a longer follow-up period. No benefits of bosentan treatment were observed, with an excess of adverse events in the treatment arm. Similarly disappointing results were seen with the mixed antagonist enrasentan (∼100-fold ET_A_ selective) in the ENCOR study (Battistini et al., 2006), and the EARTH study, which used the borderline ET_A_-selective antagonist darusentan (∼130-fold ET_A_ selective) (Anand et al., 2004). Fluid retention leading to worsening heart failure was a common feature of treatment in these studies. Whether this was due to unnecessarily high doses of drug used or concomitant ET_B_ in addition to ET_A_ receptor blockade, and, indeed, whether this could be managed with an appropriate diuretic regimen would not become apparent until the trials a decade later in hypertension and chronic kidney disease (CKD).

Unfortunately, of these four trials, only the EARTH study has been published in more than abstract form (Mylona and Cleland, 1999; Abrahams, 2001; Teerlink, 2002). Until the full dataset from all of these studies is put into the public domain there cannot be a full independent analysis of the results from which patients may potentially benefit. Of the four studies described, it is clear that only one (EARTH) measured the critically important biomarker of significant ET_B_ receptor blockade, plasma ET-1 (MacIntyre et al., 2010). This rose in EARTH and other studies (Luscher et al., 2002; Anand et al., 2004), suggestive of significant ET_B_ receptor blockade. Therefore, it may well be that a truly ET_A_-selective approach has not yet been studied in patients with CHF, and there is no trial of ET_A_ versus ET_A/B_ receptor antagonism. Unfortunately, given the negative findings to date, it is unlikely that clinical trials will be conducted in the future. However, heart failure is a heterogeneous condition and diastolic heart failure and cor pulmonale are two conditions where the potential benefits of ET receptor blockade remain unstudied.

#### 2. Primary Hypertension.

Based on its vasoconstrictor and pressor effects Yanagisawa and colleagues first speculated that ET-1 might be involved in the pathogenesis of hypertension (Yanagisawa et al., 1988). Vascular production of ET-1 is increased in most salt-sensitive animal models of hypertension (Schiffrin, 2005), and the hypertensive effects of ET-1 appear to be at least partly dependent on salt, as chronic infusion of ET-1 only increases blood pressure (BP) when combined with a normal or high-sodium diet but is absent on a low-sodium diet (Mortensen et al., 1990; Mortensen and Fink, 1992; Pollock and Pollock, 2001; Schiffrin, 2005). Both selective ET_A_ receptor and mixed ET_A/B_ receptor antagonists effectively reduce blood pressure in models of hypertension that are accompanied by an increase in ET-1 (Moreau, 1998).

Interestingly, Ergul et al. (1996) reported that levels of immunoreactive plasma ET were increased in African-American patients (who usually present with salt-sensitive hypertension) despite being treated with a range of antihypertensive therapies. Although some studies have observed increased plasma ET-1 concentrations in hypertensive patients compared with healthy individuals, this is not always evident (Davenport et al., 1990; Saito et al., 1990; Schiffrin and Thibault, 1991; Cody et al., 1992). However, because ET-1 is mainly secreted abluminally and rapidly cleared from the circulation by ET_B_ receptors (Fukuroda et al., 1994b), plasma levels may not be an accurate representation of ET system activation. Local studies with ET receptor antagonists have shown that both selective and mixed antagonists increase forearm vasodilation and reduce BP in hypertensive patients to a greater extent than in healthy individuals (Cardillo et al., 1999). In addition, this response was greater with mixed antagonism than selective ET_A_ receptor blockade, showing the important contribution of ET_B_ receptor-mediated vasoconstriction in hypertension. However, this different response in hypertensive patients and healthy individuals is not always evident (Martin et al., 2002).

The first chronic dosing study in humans investigating the potential of an ET receptor blocker as an antihypertensive agent administered the mixed receptor antagonist bosentan as a single agent in ∼300 patients with mild to moderate primary hypertension for 4 weeks (Krum et al., 1998). A reduction in systolic (−8 mmHg) and diastolic (−6 mmHg) BP was achieved with a daily dose of 500 or 2000 mg bosentan that was similar to that achieved with an angiotensin converting enzyme inhibitor. In general, the drug was well tolerated.

Although use of a mixed ET receptor antagonist successfully lowered BP, selective ET_A_ receptor antagonists may be as effective. Darusentan, an ET receptor antagonist that has an ET_A_:ET_B_ receptor affinity of ∼130:1 (Rohmeiss et al., 1998) was administered to ∼400 patients with stage 2 primary hypertension (Nakov et al., 2002). Within 1 week of a daily administration of 10, 30, or 100 mg darusentan, a significant dose-dependent reduction in systolic BP (10 mg: −6 mmHg; 30 mg: −7 mmHg; 100 mg: −11 mmHg) and diastolic BP (10 mg: −4 mmHg; 30 mg: −5 mmHg; 100 mg: −8 mmHg) relative to placebo was observed. This decrease was maintained throughout the 6 weeks of the study. Flushing and peripheral edema were seen in the treatment arms only and in a dose-dependent manner. Although these studies show that both selective ET_A_ and mixed ET receptor antagonism can reduce BP, no studies have made a direct comparison of the different antagonists.

#### 4. Resistant Hypertension.

Resistant hypertension is hypertension that remains uncontrolled despite taking three or more antihypertensive agents of different classes (usually including a diuretic) at appropriate doses. Darusentan was administered as an add-on therapy in ∼100 of these patients in a randomized, double-blind, placebo-controlled trial. Darusentan was initially administered at 10 mg a day and titrated up to 100 mg over 8 weeks. Compared with placebo, BP showed a progressive decline with systolic BP −11.5 mmHg and diastolic BP −6.3 mmHg lower at the end of the study (Black et al., 2007). Two further clinical trials reported in patients with resistant hypertension both administering darusentan at 50, 100, and 300 mg over 14 weeks. In the first of these studies (in 379 patients), all doses resulted in similar and clinically relevant decreases in office systolic BP (>15 mmHg) that were greater than the placebo group, with more patients in the darusentan groups achieving BP goal (Weber et al., 2009). However, in the second study, a similar decrease in systolic BP was observed in the placebo (−14 mmHg) and darusentan (−15 mmHg) groups (Bakris et al., 2010). From these studies in resistant hypertension, two showed a large placebo effect in office blood pressure measurements that made interpreting and analyzing the results challenging (Black et al., 2007; Bakris et al., 2010). However, in both studies, results from ambulatory blood pressure monitoring showed a significant decrease in mean 24-hour systolic BP of ∼10 mmHg, which was greater than the placebo groups (∼2 mmHg). In these studies, ambulatory BP monitoring was also able to provide the additional information that both systolic and diastolic BP reductions with darusentan were maintained over the 24-hour period, resulting in an improvement in the diurnal variation in BP. The primacy of ambulatory BP measurement above office BP measurements, where both are available in these studies, has been noted (Webb, 2010). Unfortunately, after the failure of the second darusentan study to meet its primary end point of a reduction in office BP, further studies into the chronic administration of darusentan in resistant hypertension were terminated.

#### 5. Chronic Kidney Disease.

The management of CKD in the clinic focuses on BP and proteinuria reduction, both key parameters that limit CKD progression and reduce the associated cardiovascular risk. In addition, there are a number of other aspects of CKD where there may be a role for the ET system and so where ET receptor antagonism may offer beneficial effects—these include endothelial dysfunction, atherosclerosis, arterial stiffness, and mineral bone disease. Clinical trials with ET receptor antagonists using these parameters as end points in CKD are limited. Other potential clinical applications include ischemia/reperfusion, which is an important cause of acute kidney injury, associated with high mortality rates and the development of CKD. There is an unmet need to prevent renal injury. Experimental studies in rats (Barrera-Chimal et al., 2016) and dogs (Krause et al., 1997; Roux et al., 1999) suggest a major role for ET and potential for repurposing ET antagonists. Rhabdomyolysis and other causes of massive myoglobin release are often complicated by an acute ischemic renal failure. In a rat model of rhabdomyolysis, bosentan was able to partly block a number of deleterious parameters including the decrease in creatinine clearance, increase in proteinuria, and the tubular necrosis (Karam et al., 1995).

#### 6. Hypertension Associated with Chronic Kidney Disease.

In nondiabetic hypertensive CKD patients, the systemic vasodilation seen with acute ET_A_ receptor blockade (associated with a reduction in BP of ∼10 mmHg) (Dhaun et al., 2009a) is attenuated by concomitant ET_B_ receptor antagonism (Goddard et al., 2004b), suggesting that, at least in this disease state, vasoconstrictor ET_B_ receptor activity is less important than ET_B_ vasodilatory function. Acute ET_A_ receptor antagonism also improves renal hemodynamics with a fall in renal vascular resistance and an increase in renal blood flow (Dhaun et al., 2009a). These changes are accompanied by no measurable effect on glomerular filtration rate (GFR). There is a concomitant fall in the filtration fraction, suggesting that ET-1 induces an ET_A_ receptor-mediated vasoconstriction, preferentially affecting the efferent arterioles, although not excluding effects on mesangial cells and the filtration coefficient (Dhaun et al., 2009a).

In a similar nondiabetic proteinuric population, chronic dosing with a selective ET_A_ receptor antagonist also reduces BP, albeit to a lesser extent (Dhaun et al., 2011). In this study, a fall in systolic, diastolic, and mean BP of ∼4 mmHg was observed. Of note, in this trial, patients already had good BP control (∼125/75 mmHg) conforming to current CKD guidelines. Interestingly, in both these acute and chronic studies (Dhaun et al., 2009a, 2011), the majority of patients studied were already taking ACE inhibitors. Data from healthy subjects suggest a synergy between ET_A_ receptor antagonism and ACE inhibition that is not only dependent on an unblocked ET_B_ receptor but is also associated with a significant natriuresis (Goddard et al., 2004a). This is important clinically because patients with CKD are generally prescribed ACE inhibitors not only for BP control but also for their renoprotective effects. There are currently no studies using mixed ET_A/B_ antagonism. However, subgroup analysis of the DORADO study, which looked at the borderline selective antagonist darusentan in resistant hypertension and where the top doses may have led to a degree of ET_B_ receptor blockade, suggests that a combined ET_A/B_ blocking strategy may also be of benefit in lowering BP in nondiabetic CKD (Weber et al., 2009).

In diabetic nephropathy, only one study has shown a fall in BP. This used the ET_A_-selective antagonist atrasentan (ET_A_:ET_B_ receptor selectivity of ∼1200:1), and patients were dosed for 8 weeks. There was a fall in systolic BP of ∼8 mmHg and diastolic BP of ∼6 mmHg (Kohan et al., 2011a). Although the study of darusentan in resistant hypertension included a significant proportion of subjects with diabetes (∼40%), it is unclear how many had albuminuria and so coexistent nephropathy. Interestingly, dual blockade with BQ123 and BQ788 or selective ET_A_ antagonism improved endothelium-dependent vasodilatation in an experimental study in patients with type 2 diabetes and coronary artery disease but did not differ between the two treatments (Rafnsson et al., 2014).

#### 7. Proteinuria.

Proteinuria is one of the manifestations of glomerular hypertension and has its origin in physical destruction of the glomerular filtration barrier. Albuminuria is incrementally associated with increased cardiovascular risk in both individuals with pre-existing risk (such as hypertensive patients) (Ibsen et al., 2005) and in individuals with no known risk factors (Wang et al., 2005). This is true even in the presence of normal renal function (Freedman et al., 2005). Importantly, in patients with hypertension, reduction of albuminuria—at least with blockers of the renin-angiotensin system—confers cardiovascular protection (Ibsen et al., 2005).

Both acute (Goddard et al., 2004b; Dhaun et al., 2009a) and chronic (Dhaun et al., 2011) selective ET_A_ blockade have been shown to reduce proteinuria in patients with nondiabetic proteinuric CKD. In the acute studies, these effects were abolished by concomitant ET_B_ receptor antagonism (Goddard et al., 2004b). As for BP reduction, mixed ET_A/B_ antagonism has also been shown to reduce proteinuria in nondiabetic CKD (Weber et al., 2009). However, all these subjects had, at most, microalbuminuria compared with the chronic dosing study with the selective ET_A_ antagonist (Dhaun et al., 2011), which included not only a wide range of nondiabetic proteinuric renal diagnoses (membranous glomerulopathy, IgA nephropathy, focal segmental glomerulosclerosis, for example) but also varying proteinuria (0.3–8 g/day).

The largest studies looking at the efficacy of ET receptor antagonism in reducing proteinuria have been in patients with diabetic nephropathy. The first of these was a phase II study investigating the effects of 12 weeks of treatment with avosentan, a relatively ET_A_-selective antagonist (ET_A_:ET_B_ blockade ∼300:1), on albuminuria in 286 patients with diabetic nephropathy already receiving renin-angiotensin system blockade. Subjects had a creatinine clearance of ∼80 ml/min and albuminuria of ∼1500 mg/day (Wenzel et al., 2009). Avosentan, at all doses used, reduced albuminuria by 20–30%. Fluid retention was a problematic side effect, however, and was more apparent at the higher doses of avosentan. Based on their interpretation of the data, the pharmaceutical company launched a phase III trial (ASCEND) examining the effects of avosentan on renal disease progression or death in type 2 diabetic nephropathy (Mann et al., 2010). A total of 1392 patients were enrolled and avosentan reduced urine albumin-to-creatinine ratio (ACR) by 40–50% compared with the 10% reduction seen with placebo. However, the trial was terminated early because of greater serious adverse cardiovascular events in the avosentan groups, including a threefold increase in the incidence of congestive heart failure. It is likely that the doses of avosentan used were too high and so blocked the ET_B_ receptor as well as the ET_A_, an effect that would be predicted to promote fluid retention (Kohan et al., 2011b). This was also confirmed by a study in healthy volunteers that showed that increasing doses of avosentan were associated with sodium retention study (Smolander et al., 2009), and this was certainly the case at the dose used in the ASCEND study. Furthermore, the ASCEND trial involved patients with advanced kidney disease who may have been more likely to retain fluid.

The failure of the ASCEND trial underscored the importance of careful patient selection and ET antagonist dosing in CKD. A subsequent study evaluated the effects of doses of atrasentan (more ET_A_ selective than avosentan) or placebo given for 8 weeks on ACR in 89 subjects with diabetic nephropathy receiving stable doses of renin-angiotensin system inhibitors (Kohan et al., 2011a). Atrasentan reduced ACR by ∼40% compared with the 11% reduction seen with placebo. The only adverse event was mild to moderate peripheral edema, which was dose related.

As yet, it remains unclear as yet to what extent these effects on proteinuria are explained by BP reduction alone. In both the acute and chronic dosing studies in nondiabetic CKD using a selective ET_A_ receptor blocking approach, there was, as expected, a correlation between the changes in BP and proteinuria, with a greater fall in urinary protein seen in those patients with the greater fall in BP. However, both studies also had an active control that matched the reduction in BP seen with ET_A_ antagonism. Despite a similar fall in BP with both the active control and the ET_A_ antagonist, proteinuria fell to a greater extent with the latter supporting a BP-independent effect. Furthermore, a renal hemodynamic study after chronic selective ET_A_ antagonism suggests that falls in both GFR and filtration fraction are in part responsible for the reduction in proteinuria (Dhaun et al., 2011). This is similar to the effects seen with ACE inhibitors and so may translate to longer term renoprotection. In the atrasentan study in diabetic nephropathy the reduction in ACR was evident after 1 week of treatment and was associated with a fall in BP, suggesting that the initial antiproteinuric effect of atrasentan may be hemodynamic.

It is noteworthy that the problem with hyperkalemia associated with the use of ACE inhibitors, especially in those with CKD, is not an issue with ET receptor antagonists. Furthermore, the fluid retention (and anemia that results secondary to this) consistently demonstrated in clinical trials with both selective ET_A_ and mixed ET_A/B_ agents has been shown to be manageable (Weber et al., 2009) and to not affect the antiproteinuric benefits of these drugs (Kohan et al., 2015).

#### 8. Broader Cardiovascular Risk: Endothelial Dysfunction, Atherosclerosis, and Arterial Stiffness.

The endothelium is a crucial regulator of vascular tone (Endemann and Schiffrin, 2004) and its function is impaired in heart failure, hypertension, and CKD, with a shift toward reduced vasodilation, associated with a proinflammatory and prothrombotic state. Endothelial dysfunction is recognized to be a key early determinant in the progression to atherosclerosis with the development of active vascular microcalcifications (Irkle et al., 2015) and is now well established to be independently associated with increased cardiovascular risk (Lerman and Zeiher, 2005) and GFR loss (Perticone et al., 2004). Animal models show that ET-1 contributes to endothelial dysfunction (Amiri et al., 2004), and ET receptor antagonism, predominantly with selective ET_A_ antagonists, improves NO-mediated endothelial function (Barton et al., 1998; Best et al., 1999; Bauersachs et al., 2000). In addition to its effects on BP, ET-1 is proinflammatory (Dhaun et al., 2006) and is implicated in the development of atherosclerosis. Several animal models have shown benefit of both selective and mixed ET receptor antagonism in the development of atherosclerotic lesions (Kowala et al., 1995; Barton et al., 1998; Babaei et al., 2000; d'Uscio et al., 2002). Clinical data are limited but support a role for the ET_A_ receptor in coronary vascular tone and endothelial dysfunction in coronary artery disease (Halcox et al., 2001, 2007). Furthermore, endothelial dysfunction is a feature of aging, and in these subjects compared with younger ET-1 contributes to endothelial dysfunction (Westby et al., 2011). Also, in older subjects with early atherosclerosis long-term administration of a selective ET_A_ receptor antagonist improves coronary endothelial dysfunction (Reriani et al., 2010).

Arterial stiffness is linked to endothelial dysfunction (Oliver and Webb, 2003) and the two commonly coexist in patients at increased cardiovascular risk. A number of interventions that reduce arterial stiffness also improve endothelial function (Oliver and Webb, 2003). To date, there have been few studies addressing the relationship between these two markers of cardiovascular disease after treatment. However, both animal and human studies suggest that the endothelium is an important regulator of arterial stiffness. ET-1 increases arterial stiffness in animals (Amiri et al., 2004) and humans (Vuurmans et al., 2003). There are only two studies, one acute (Dhaun et al., 2009a) and one chronic (Dhaun et al., 2011), both using a selective ET_A_ receptor antagonist approach in nondiabetic CKD. These show a reduction in arterial stiffness with treatment, but the extent to which this is independent of BP is unclear. Interestingly, in the acute study (Dhaun et al., 2009a), the reduction in arterial stiffness seen with ET antagonism was greater in those taking dual ACE inhibitor/angiotensin receptor blocker treatment than in those receiving an ACE inhibitor alone (Dhaun et al., 2009b). Indeed, there was a similar effect for proteinuria in this study.

#### 9. Chronic Pain.

In 1990 a report was published that described a deep muscular pain associated with infusion of intra-arterial ET-1 that was exacerbated both by touch and muscle contractions (Dahlof et al., 1990). Since then there has been a significant amount of research dedicated to the contribution of the ET system to pain. This has been comprehensively reviewed recently by Smith et al. (2014) and will only be summarized briefly.

ET-1 and its receptors are widely distributed throughout the central and peripheral nervous system pain pathways. It is now recognized that not only does ET-1 cause pain directly (Namer et al., 2008) but it also sensitizes pain pathways to other painful stimuli such as capsaicin (Piovezan et al., 1998). The pain that ET-1 induces directly is mediated via mechanosensitive C-nerve fibers, but the neuronal firing pattern is distinct to that elicited by capsaicin and histamine (Smith et al., 2014), which give rise to short bursts of activity compared with longer lasting responses with ET-1. Most of the studies examining the role of ET-1 in specific types of pain are preclinical and cover a range of diseases including arthritis, cancer, diabetes, sickle cell disease, and various types of neuropathic pain (Smith et al., 2014). It appears that the ET_A_ receptor is consistently involved in pain transmission pathways, whereas the role of the ET_B_ receptor is less clear and varies between organs, the type of pain, and across species (Smith et al., 2014).

To date, there are only a few published clinical studies and these are limited to cancer and sickle cell disease. ET-1 is thought to be involved in a number of aspects of cancer pathophysiology including pain. This extends to many different cancers, including, breast, brain, pancreatic, prostatic, and colon. Clinical studies are largely restricted to castration-resistant prostate cancer. Here, addition of an ET receptor antagonist to standard therapy allows for a better pain profile in patients with bony involvement (Albany and Hahn, 2014).

Sickle cell disease is characterized by vasoocclusive events, which may occur in a wide range of vessels, ranging from the microvasculature to the muscular arteries. Cell dehydration is an important step in the formation of sickle cells, and ET has been suggested to mediate this action in vitro by activating Ca^2+-^gated K^+^ channels via ET_B_ receptors on mouse erythrocytes (Rivera et al., 1999). In vitro studies have shown that vascular endothelial cells exposed to sickled erythrocytes results in elevations in ET-1 transcripts and peptide, and it has been speculated that this increase may contribute to vasoconstriction (Phelan et al., 1995). In patients with sickle cell disease, plasma ET-1 concentration correlates with pain scores during crises (Schlenz et al., 2012). Phelan et al. (1995) showed that exposure of human endothelial cells to sickled sickle erythrocytes caused an increase in ET-1 transduction and peptide release, suggesting a cause for vascular occlusion. In addition, ET was found to activate Ca^2+^gated K^+^ channels via ET_B_ receptors in mouse erythrocytes and to cause cell dehydration, an important role in the pathogenesis of sickle cell disease (Rivera et al., 1999). At present, however, there are no studies using ET receptor antagonists in this condition.

#### 10. Subarachnoid Hemorrhage.

Cerebral vasospasm constitutes the only medically treatable cause of disability and death in patients suffering a subarachnoid hemorrhage (SAH). Although ET-1 does not normally contribute to cerebral vascular tone (Andresen et al., 2006), its synthesis increases and ET receptors are upregulated after cerebral ischemia, and this may contribute to vascular dysfunction and brain injury (Matsuo et al., 2001; Petrov and Rafols, 2001).

Although the preclinical data favored the use of selective ET_A_ receptor antagonists in this condition, the early clinical trials using mixed antagonists were inconclusive (Chow et al., 2002). Early data with the selective ET_A_ receptor antagonist clazosentan suggested that it prevented cerebral vasospasm after SAH, although the clinical outcome remained unchanged (Macdonald et al., 2007). This led on to several larger clinical studies of longer duration.

A meta-analysis and systematic review published in 2012, which included data from five studies incorporating 2601 patients, concluded that ET receptor antagonists do not affect the functional outcome after SAH, although they reduce vasospasm (Vergouwen et al., 2012). This was no different for selective or mixed antagonists. Furthermore, the use of these agents was associated with an increased incidence of complications, including fluid overload and anemia, both recognized side effects of this class of drug and poor prognostic indicators in SAH. At present, ET receptor antagonists do not have a role in the medical management of SAH, but the results of future studies, perhaps in more defined cohorts using drugs with an improved side effect profile, are awaited.

#### 11. Cancer.

Dysregulation of the ET system is now recognized to play an important role in the biology of several solid tumors, including bladder, breast, colon, lung, ovarian, and prostate cancers (Rosano et al., 2013a). ET-1 signaling is important in a number of tumorigenic processes, including disease initiation, angiogenesis, epithelial to mesenchymal transition, and metastasis. Expression profiles of ET receptors differ between cancer types, and these may be classified into three types (Irani et al., 2014): those that predominantly express ET_A_ receptors, such as breast, colon, and prostate; cancers that mainly express ET_B_ receptors, such as melanoma and several brain tumors (glioblastoma is one example); and those cancers that express both ET_A_ and ET_B_ receptors, such as bladder, lung, and ovary. Of note, there is a correlation between ET receptor expression and disease outcomes such as metastasis and, more importantly, patient survival (Rosano et al., 2013a,b). The mitogenic effects of ET-1 are mediated via ET_A_ receptors in several epithelial tumors (Grant et al., 2003) and by ET_B_ in nonepithelial tumors (such as melanoma) (Lahav et al., 2004). ET-1, through its actions on ET_B_, is also mitogenic for lymphatic endothelial cells, and so ET_B_ receptor expression in cancer is associated with lymphatic spread and ET_B_ receptor blockade impairs lymphangiogenesis (Spinella et al., 2010). Similarly, ET_A_ receptor overexpression is associated with tumor metastasis (Mai et al., 2006). Thus, clinical trials of ET receptor antagonists in cancer have focused on both selective ET_A_ and mixed ET_A/B_ blocking approaches.

Despite the promising results of ET blocking strategies, used in cell lines and animal models of cancer, the clinical trials have been less rewarding. The two largest studies have been in advanced prostate cancer using the selective ET_A_ antagonists atrasentan (809 patients) and Zibotentan (Astra Zeneca) (312 patients) (Carducci et al., 2007; James et al., 2010). A trial in metastatic melanoma using the mixed ET_A/B_ antagonist bosentan was also disappointing (Kefford et al., 2007). A similarly negative study was recently reported using zibotentan in advanced ovarian cancer (Cognetti et al., 2013).

Given the lack of specific biomarkers that might predict clinical response to treatment with an ET receptor antagonist, either as monotherapy or in addition to standard treatment, it currently remains unclear if there are specific subgroups of patients that might benefit. The issue of whether a selective ET_A_ or mixed ET_A/B_ blocking approach is better is also unresolved, although this is likely to vary between cancer type. Furthermore, given the significant crosstalk between the ET system and other pathways involved in cancer biology, such as vascular endothelial growth factor and the epidermal growth factor receptor (Rosano et al., 2013a,b), it might be that the role of ET receptor antagonists is in combination with agents targeting these pathways. Finally, stage of disease may be an important factor. Many of the clinical trials have been conducted in late-stage, often metastatic, disease and it is currently unclear whether specific cancers are more dependent on ET signaling in the early disease trajectory, which may then be a better target.

## VII. Concluding Highlights and Perspectives

Considerable progress has been made during the last two decades in characterizing the pharmacology of the ET signaling pathway. This has benefited from the development of the key tool compounds described in this review, with remarkably highly selective ET_A_ and ET_B_ antagonists and selective ET_B_ agonists together with radiolabeled analogs to accurately and confidently delineate the ET system in humans and animal models. A surprising omission has been that no ET_A_ agonist has been reported and remains a mystery. The use of ET_B_ agonists such as IRL1620 in chemotherapy and neuroprotection is a particularly intriguing field that warrants further investigation (Briyal et al., 2014). New strategies to block the unwanted actions of ET are emerging via small molecule epigenetic modulators as well as the appearance of the first biologic—a monoclonal to the ET_B_ receptor—although GPCRs remain a challenging target for the development of pharmacologically useful monoclonal antibodies. Increasing our understanding of regulating the ET system via microRNAs (Grant et al., 2013) may also yield new druggable targets in the future.

Two ET antagonist (bosentan, ambrisentan) are firmly established in the clinic and have proved successful for the treatment of PAH, with the next generation antagonist with improved efficacy (macitentan) being approved for clinical use, although it remains unclear to what extent ET antagonist modify disease progression. Surprisingly, PAH remains the only clinical condition approved for ET receptor antagonists. The use of ET antagonist in heart failure has not translated from predictions arising from animal studies, although the reasons for lack of efficacy are still unclear because the results of certain clinical trials have not been fully published. However, clinical trials continue to test the repurposing of existing antagonists with the most likely clinical indication as outlined here probably targeting the kidney, including hypertension associated with CKD and diabetic nephropathy associated with proteinuria, as well as new potential indications for chronic pain and SAH. Perhaps the greatest potential for a paradigm shift is GPCR-mediated targeting of the ET system in cancer. The second strategy to block the unwanted actions of ET via ECE inhibition despite 20 years of research has not yet translated into a clinical application. Further studies are needed to understand more fully the synthesis and metabolism of ETs and how current and future putative ECE inhibitors also impact or alter other peptide transmitter systems.

Key questions remain. What is the precise molecular mechanism for long-lasting responses to ET-1, particularly in the human vasculature? What is the physiologic/pathophysiological role of ET3, and will differences emerge particularly in the CNS between ET-1 and ET-3? Do homo- or heterodimers of ET receptors exist in native tissue, and can they be exploited pharmacologically? Perhaps the greatest revolution in pharmacology is the discovery of biased ligands. Can these be exploited in the ET field? For example, a *β*-arrestin biased antagonist to block oncogenic pathways in ovarian cancer but spare the beneficial G*α* signaling, which is tumor suppressive.

Currently, we cannot yet distinguish pharmacologically between an ET_A_ receptor expressed on different cell types such as a myocyte versus a smooth muscle. However, many mechanistic hypotheses about the physiologic and pathophysiological role(s) of the ET system have been tested with the new technological tools to knockout, knock in, or alter the levels of gene expression globally and in specific cell types. We have highlighted the noteworthy studies and conclusions from over 100 publications, reporting a remarkable 28 different genetic modifications of the ET system. In addition, we provide insight for potential areas of interest for future research.

Global KO of ECE-1, ET-1, and ET_A_ are all embryonic lethal and found to be critical for neural crest patterning, proper jaw identity and development, and cardiovascular development, thus greatly expanding our understanding of the role of the ET-1 system in development. Global KO of ET-3 and ET_B_ led to the demise of pups by 3–4 weeks of age. This has led to the discovery that ET-3 and ET_B_ receptors are vital for the development of vagal neural crest-derived enteric neurons and the trunk neural crest-derived epidermal melanocytes. These observations resulted in ET-3/ET_B_ being identified as a causative pathway in the development of intestinal megacolon or Hirschsprung disease. Rescuing this lethal megacolon phenotype by insertion of a functional ET_B_ transgene directed to expression in neuronal tissue allows the intestine to develop normal yet dysfunctional ET_B_ receptor expression in all nonneuronal tissues. The rescued ET_B_ receptor-deficient mouse and rat established the critical role of ET_B_ signaling in salt-sensitive hypertension.

A large body of work with a range of genetic modifications with the ET system in the kidney and endothelium found that collecting duct-specific ET-1, ET_B_, and ET_A/B_ KO mice all show a salt-sensitive hypertensive phenotype. A common side effect of pharmacological ETA antagonists is edema. Genetic models of cell-specific ET_A_ KO in the CD, whole nephron, cardiomyocyte, or smooth muscle pointed toward the collecting duct and smooth muscle ET_A_ receptors being responsible for the fluid retention. CD ET_A_ receptor activation also contributes to the urine concentrating mechanisms. ET_A_ and ET_A/B_ antagonists are clinical therapeutic agents for PAH in clinical trials for diabetic nephropathy, thus the elucidation of the signaling mechanisms of CD and/or smooth muscle ET_A_ receptor activation would be warranted to study in the future.

Generally, overexpression of ET-1 or ET-2 peptide results in increased inflammation and fibrosis in many tissues, especially the heart. Using a novel hypomorphic low expressing ET-1 gene and hypermorphic high expressing ET-1 gene, it was found that ET-1 is vital for maintaining normal contractile function and for ensuring that the myocardium has sufficient collagen to prevent overstretching; even a modest decrease in ET-1 expression is sufficient to cause cardiac dysfunction. Cardiomyocyte-specific ET-1 KO studies showed that cardiac ET-1 is critical for cardiac function and cardiomyocyte survival in aging or stressed mice. Proinflammatory and/or profibrotic diseases, such as cardiac hypertrophy or acute kidney injury, were attenuated in endothelial cell ET-1 KO mice. Thus, selectively antagonizing endothelial-derived ET-1 would be advantageous to block inflammation and fibrosis in the heart and kidney.

Pain is one of the most prevalent aliments in many patient populations. Studies with astrocyte-specific overexpression of ET-1 suggest that increases in astrocyte ET-1 may be harmful in the setting of stroke but may alleviate chronic pain. Given the strong evidence that ET-1/ET_A_ receptors contribute to sensory nerve pain, the apparent contrast of central versus peripheral ET_A_ receptors in the pain pathway requires further investigation to determine the viability of ET receptor antagonists and the cell specificity of the ET system in the many different paradigms of pain.

In perspective, the use of over 28 different genetic modification models of the ET system has provided clear determinations of the physiologic significance of the ET system in development as well as in vascular function, fluid-electrolyte homeostasis, cardiac function, and neuronal function in a variety of cell types. These models have proven to be invaluable to distinguish the physiologic from the pathophysiological aspects of the ET system. Newer genetic modification tools that manipulate the level of expression of the ET system in various cell types will further our understanding of molecular mechanisms for therapeutic advantage. Studies on cell-specific modification in the ET system in mice provide the proof of principle, for example collecting duct ET_A_ receptors are responsible for the unwanted side effect of blocking ET_A_ receptors to cause fluid retention. The challenge for future in ET pharmacology will be to discover biased agonists and antagonists that can selective activate or block ET receptors expressed on different cell types.

## Abbreviations

A192621: (2R,3R,4S)-4-(1,3-Benzodioxol-5-yl)-1-{2-[(2,6-diethylphenyl)amino]-2-oxoethyl}-2-(4-propoxyphenyl)-3-pyrrolidinecarboxylic acid
ACE: angiotensin converting enzyme
ACR: albumin-to-creatinine ratio
AKI: acute kidney injury
AMP: cyclic adenosine monophosphate
ANGII: angiotensin II
ANP: atrial natriuretic peptide
AQP2: Aquaporin 2
AVP: arginine vasopressin
Big ET-1: big endothelin-1
Big ET-2: big endothelin-2
Big ET-3: big endothelin-3
BP: blood pressure
BQ123: cyclo-[D-Asp-L-Pro-D-Val-L-Leu-D-Trp-]
BQ3020: [Ala11,15]Ac-ET-1(6–21)
BQ3020 ([Ala^11,15^]Ac-ET-1_(6–21)_); BQ788 (D-Norleucine: N-(((2R,6S)-2,6-dimethyl-1-piperidinyl)carbonyl)-4-methyl-L-leucyl-1-(methoxycarbonyl)-D-tryptophyl-)
CD: collecting duct
CHF: chronic heart failure
CKD: chronic kidney disease
CNS: central nervous system
CPP: cell penetrating peptide
C terminus: carboxy terminus
darusentan: LU135252, (2S)-2-[(4,6-Dimethoxy-2-pyrimidinyl)oxy]-3-methoxy-3,3-diphenylpropanoic acid
ddAVP: desmopressin
DOCA: deoxycorticosterone acetate
D*β*H: dopamine-*β*-hydroxylase
E: embryonic day
EC: endothelial cell
ECE-1: converting enzyme-1
ECE-2: converting enzyme-2
EDNRB: ET_B_ gene
ENaC: epithelial sodium channel
ESRD: end stage renal disease
ET-1: endothelin-1
ET-2: endothelin-2
ET-3: endothelin-3
ET_A_: endothelin A receptor
ET_B_: endothelin B receptor
FR139317: *N*-[(hexahydro-1-azepinyl)carbonyl]L-Leu(1-Me)D-Trp-3(2-pyridyl)-D-Ala
GFR: glomerular filtration rate
GPCR: G-protein coupled receptor
H: high expressing
IRL1620: Suc-(Glu9, Ala11,15)-ET-1(8–21)
IRL-2500: (2S)-2-({(2R)-3-(4-Biphenylyl)-2-[(3,5-dimethylbenzoyl)(methyl)amino]propanoyl}amino)-3-(1H-indol-3-yl)propanoic acid
*K*_D_: equilibrium dissociation constant
*K*_i_: inhibition constant
KO: knockout
L: low expressing
l-NAME: LU420627, (S)-3-[2-(3,4-dimethoxyphenyl)ethoxy]-2-(4,6-dimethylpyrimidin-2-yloxy)-3,3-diphenylpropionic acid; *N^ω^* -nitro-l-arginine methyl ester
NEP: neutral endopeptidase or neprilysin
NOS: nitric oxide synthase
NOx: nitrite and nitrate
N terminus: amino terminus or NH_2_ terminus
PAH: pulmonary arterial hypertension
PD156707 (Sodium: (2Z)-2-(1,3-benzodioxol-5-yl)-4-(4-methoxyphenyl)-4-oxo-3-(3,4,5-trimethoxybenzyl)-2-butenoate)
PET: positron emission tomography
PGE_2_: prostaglandin E2
preproET: pre-proendothelin
RO 46-2005 (4-tert-butyl-N-[6-(2-hydroxyethoxy)-5-(3-methoxyphenoxy)pyrimidin-4-yl]benzenesulfonamide); RO468443: N-{6-[(2R)-2,3-Dihydroxypropoxy]-5-(2-methoxyphenoxy)-2-(4-methoxyphenyl)-4-pyrimidinyl}-4-(2-methyl-2-propanyl)benzenesulfonamide
ROS: reactive oxygen species
RVSP: right ventricular systolic pressure
SAH: subarachnoid hemorrhage
SB209670: (1S,2R,3S)-1-(1,3-Benzodioxol-5-yl)-3-[2-(carboxymethoxy)-4-methoxyphenyl]-5-propoxy-2-indanecarboxylic acid
SM: smooth muscle
TGF: tissue growth factor
VEETKO: vascular endothelial cell ET-1 knockout mouse
VIC: vasoactive intestinal contractor
